# Supplementary material for: Nucleotide Composition of Ultra-Conserved Elements Shows Excess of GpC and Depletion of GG and CC Dinucleotides
Source: Genes (Basel). 2022 Nov 7;13(11):2053. doi: 10.3390/genes13112053 (PMC9690913; doi:10.3390/genes13112053)
Supplement: Supplementary file 1 [file genes-13-02053-s001.zip › Figure S1.pdf]

FIGURE S1. Characterization of ten UCNEs inside human FTO gene (alpha-ketoglutarate dependent dioxygenase).

Figure S1a. Screenshot of NCBI genomic viewer of FTO gene on chromosome 16 (Build-38). Positions of ten UCNEs inside extra-large introns of FTO gene are shown by red arrows. Their nucleotide sequences are shown beneath the scheme. Their UCNEbase identifiers are the following: IRXB_Mateo, IRXB_Molly, IRXB_Napoleon, IRXB_Oberon, IRXB_Othell, IRXB_Pablo, IRXB_Pandora, IRXB_Penelope, IRXB_Poseidon, IRXB_Roxane. Their positions in the Fasta-formated file are based on the Build37 version of the reference human genome.

FTO gene

NCBI genome viewer

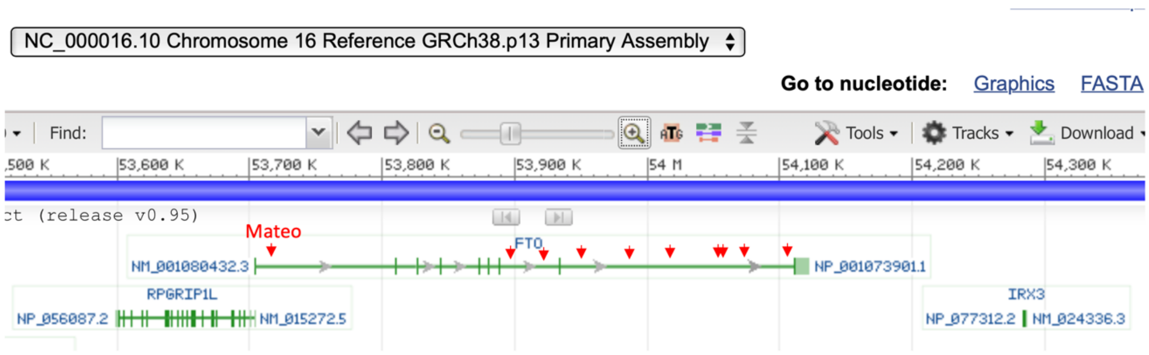


```
>IRXB_Mateo id=15047 pos=chr16:53756950-53757285 (FTO-1)
AAGTTACTTTAGTGTAACTGCTGCTGTCACCTTTGCCCTTAGCTAATCAA
ATAGTTTACCTTGAGGGAAGCATTTATCTCCCACTTTCATTATTATTATT
TTTGTAGCTGTAAAGATATATATTTTATGTCTGCAGAAGGCTGTAACAGT
GAAACGAGGTATTGATCTGCTGTATTTAGCAATTTCTTTCCACCTTGCCA
TCAATAGCATGTCAGCATCTGTCTGTACCGTGGTTGACCTCACAAATAGC
TCTTTATGCTCCCATTGGATTCAAATGATATAGTATGCTGAAAACTAAAT
CAATGAATGATTATAAAGTTTTTAGTATTATTTCAT
>IRXB_Molly id=15052 pos=chr16:53929295-53929573
CGAAAGCACAAAGGACTGTGTTTATGATTCTCTTTGAAGTGTTTTTGCAA
CTGGATAAGGAAAATTTTAGAAACCGTCTGATAGTGTTCAGGGTAAATAC
TTAAACCCCTTTAGAGAATTTCACACACAGATACTGTGCAGCAGAAAAAA
GGGATACTTATGTTTCTTAAAGCTGCTACAGCCTACAGCTTTGTACTTAA
AGACAGAGGTGTTGAATAGAATTCTACTGAGGATACATCCAGTGACAGTA
TCTCCAGGAACATCAGAGCAAACCAGTGG
>IRXB_Napoleon id=15056 pos=chr16:53957123-53957516
AGTAGTTAATGTACTGTATGTGAAAGATTTAAGACTGGTTGGAAAGCTCT
GCTTCTGTTTTATTAGGCTGCTATTGAAAAGCCAACAGCATAACTTCGAC
TAGGTGCTTTGAGATTTCTTAATGGGCTGTCAGGGGCAAGACACCAGGGC
TGTGCTATACTAGCTCTTTCCTGTACATTAAAACAACAGGGGCCAAACGA
TCACATACAGAAGTATGAATTACTTGATGCTTTCTCTGTTCCATATGGCC
AGCCATGTCTTTGGCAATTAATCATGTACAAAATCAGTCGAGTGCTCTCT
GAAGCATTGAAGTCAACATTTAGTTGTACCTGTGCCAGTAGGGGCAGTGG
```

```
GGTTGCAGGCGTAAAATAGCAGGATGAGGGGCTGCTGGCGCCAA
>IRXB_Oberon id=15058 pos=chr16:53980805-53981054
TTGCTTCATTTCCAGTTTCACCATCAATTACGCCATGTGTTTCATCCAGT
TGGTGCTACCCTCCCAGTGTTAATTATAACCTACAAGACTGTTACAGTTA
ATTTTATGGCAAGCATATTGTCTCTTCAAGGCTCCAGAGCAGCTCATAAA
TTATCTTGAAGGTAAAATGTAACTTGGGGAACTAGTTATGAAAATTCCAT
CCATCTCGCTTAGGAGCTGCAGCTGCTACTGTCAGCTGCCTTGCTGCTGC

>IRXB_Othell id=15069 pos=chr16:54020764-54020989
AAAGACCAACTCAAATTCTGTCTGCTGTAGCTTTGATCCTTTTCATTTAA
TTCTAAGTACACTTGTTATTATAGTAACTGTTGTTTGTATGTTATTTTAG
CTTTTTACTTAAGTAGCTATTTAGCAATTTAAATGAAAATGTAATTAGGT
TACATCTGTTTTACATGCATGTAATACCACGCAGATGTAAACTTCTATTA
AAGCCATATTTTCTTTTTGGCAAGAA
>IRXB_Pablo id=15074 pos=chr16:54056490-54056818
TGTTCATTACTGCTCAAGTGACGGCTTGTCAATAGCAGTGCTTTTTAGTT
TGTTCTAGTCAGCACTTGCTGTGCATTTTGTTGGACCACTGGGAAATAAA
TACAACAAGATTATACATTTGGAATACCACAAGAGTTCACAAAAAAAACC
ACAAATGATAAATTATACACTTGGTAGAAAGTGACATGCTATCTTGTGCT
ATTAACAACCAAAGATAATTGCAACGAATTGTAATTGCAGTTTGCAACTT
ACAGTACAGTGTGTCCAACATCAATCTTAATAGTGAACAAAGCAATTTAT
ATTAAAACTTAATTCACTTGGTAACTTTC
>IRXB_Pandora id=15083 pos=chr16:54093096-54093465
ATTTACCTTGCAGCCGAGTGAAAAATGATCAGAATATTAGACTCATCTAG
CCCACATTAAGGAGCCATTGCATTTCCCGACAATTGTATGCTATCTCAAT
ATGAAAACAGAGCAATTTCAACTCATATCAGGTTATGATCGGCAATTACA
GTTGCATAATTCACCTAGTCATCTCTTTGCAGGGCCTTTATTTTCCCTCT
TGGTGACATTCCTGAGAAAGTGCCTGATAAAAATGTCATATATCATCCCA
GCTTTAGAACTCGTGAGGGGGTAATCTGCTATGCCTTGCTGAGCCCATAT
TCATCACTGGATTTTTGACTTGGAGGGGTCATGGCACTCATAATTTCCTG
TTTGATATTGTCCTCACCAG
>IRXB_Penelope id=15084 pos=chr16:54093563-54093797
ATGCTGGAGTTGGCCGTTTCGAAGATAATTAGATTTGATGTCAACATTTC
TTTATCACCTGCATCCTTTTGTGCATCTGTGTGTCAAGTTGTTCTTTCCG
GATTTATTAGCACCCTCAGAGCTACTCTCTATCTGTCAGCTTGTGTGCTG
TTTGTGTTGACAGTTGTAAAGTTAATTACTAGTACTAATGAGCATCGGGC
TTTTGGTGGACATGGCGTTTTGGACATTTAAAACT
>IRXB_Poseidon id=15090 pos=chr16:54101268-54101623
CTAAATTAAGCTAGCTATTGCTGGAGAGTGGATAAGCGGCCATTATGAAT
TATCCTTAATTATAGCGATTTCCTAAACTAATAAACCTGCTAAATCTTTA
TTGCAAAGGTTAGTTTACGCAGTGCATATAAAATCCGCATGTGCTCCGCA
TCAGCCACCAGCTGTCTGACAGTGTGTTGCTATTGCAGTGAGTGTTCACA
TAAATTTTATGTTGGTTATATTAACAGTCCGTCCATATCACTACCCACAG
ATGCCAGTAGACATAAAAATTGCTCTCTAGTGAGCCTAAATATCCCTAGA
AGCTAGTTGTGTCTAATGTTCCATGTAGTATTTGAACGTGAAAGCACCAG
ATTGGA
>IRXB_Roxane id=15096 pos=chr16:54144134-54144343
AGCTTATCAGTGCAAACAGTTTAATATTTATGCTAAGAGGATTGTCAAAA
GCAGCTTCTGTTGCTTTAATTCTTGTTTTAAATAAATAATGAGAACATTT
AAACACATTACTCTTCTTGGGGCCCCGGGGTCAGCTAATCTTATTATTTA
TGAAGTGATGTGCTACATAATAGTACTTAGTGCATGTTAACAGACGCTAT
TATCAGGGCC
```

Figure S1b. Pairwise BLAST alignments of HUMAN vs CHIKEN UCNE-Mateo inside FTO gene.
(IRXB_Mateo id=15047 pos=chr16:53756950-537572 (h37.p13))
Human UCNE element is highlighted in green. Flanking regions are not highlighted

```
Human  2906     ttttttaaaagagtTATGTCTGAACCACCTA---TGTGTCCCGATTCATTTTGCCATTTC  2962
                |||| |||||||   |  |||  ||| ||||   | ||  | | |||||||||||  || 
Chick  4511822  TTTTATAAAAGAACGACATCTATACCTCCTAGAATTTGCTCAGTTTCATTTTGCCCCTTT  4511763

Human  2963     ATCTActgctcttcctcctgtttttatttaagACTATGAAGTTACTTTAGTGTaactgct  3022
                 ||| |||||| |||  |   |||||||||||||| | ||||||||| ||||||||||||
Chick  4511762  GTCTGCTGCTCATCCCACCACTTTTATTTAAGACTGTAAAGTTACTTCAGTGTAACTGCT  4511703

Human  3023     gctgtcaccttTGCCCTTAGCTAATCAAATAGTTTACCTTGAGGGAAGCATTTATCTCCc  3082
                | ||||||||||||||| ||||||||||||||||||||||||||||| ||||||||||||
Chick  4511702  GTTGTCACCTTTGCCCTCAGCTAATCAAATAGTTTACCTTGAGGGAAACATTTATCTCCC  4511643

Human  3083     actttcattattattatttttgtagctgtaaagatatatattttatgtcTGCAGAAGGCT  3142
                |||||||||||||||||||| |||||||||||||||||||||||||||||||||||||||
Chick  4511642  ACTTTCATTATTATTATTTTCGTAGCTGTAAAGATATATATTTTATGTCTGCAGAAGGCT  4511583

Human  3143     GTAACAGTGAAACGAGGTATTGATCTGCTGTATTTAGCAATTTCTTTCCACCTTGCCATC  3202
                ||||| ||||||||||||||||||||||||||||| ||||||||||||||||||||||||
Chick  4511582  GTAACGGTGAAACGAGGTATTGATCTGCTGTATTTGGCAATTTCTTTCCACCTTGCCATC  4511523

Human  3203     AATAGCATGTCAGCATCTGTCTGTACCGTGGTTGACCTCACAAATAGCTCTTTATGCTCC  3262
                 ||| ||||||||| ||||||||||| ||||||||||||||||||||||||||||| |||
Chick  4511522  GATATCATGTCAGCCTCTGTCTGTACTGTGGTTGACCTCACAAATAGCTCTTTATGTTCC  4511463

Human  3263     CATTGGATTCAAATGATATAGTATGCTGAAAACTAAATCAATGAATGATTATAAAGTTTT  3322
                |||||||||||||||||||| ||| || ||||||||||||||||||||||||||||||||
Chick  4511462  CATTGGATTCAAATGATATACTATTCTCAAAACTAAATCAATGAATGATTATAAAGTTTT  4511403

Human  3323     TAGTATTATTTCATGACCTGGAGGCAACTGGTACT  3357
                |||||||||||||| | |   ||||||||||||||
Chick  4511402  TAGTATTATTTCATAAGCCCCAGGCAACTGGTACT  4511368
```

Figure S1c. Pairwise BLAST alignments of HUMAN vs CHIKEN FTO mRNAs.
Human coding sequence of FTO mRNA (NM_001363894.1) is highlighted in yellow.
The position of starting ATG codon is 223; while position of the stop codon is 1803.
Chicken FTO mRNA is NM_001185147.1 (CDS 81..1604),
Start codons ATG are shown in bold font.

Alignment statistics for match #1

Score	Expect	Identities	Gaps	Strand
427 bits(473)	4e-122	487/654(74%)	10/654(1%)	Plus/Plus

```
Human  807   CAGAGCAGCATACAACGTAACTTTGCTGAATTTCATGGATCCTCAGAAAATGCCATACCT  866
             |||| || | || ||| | |||||  | ||||  |||||||| |   ||||||||||| |
Chick  683   CAGAACATCTTATAACTTGACTTTATTAAATTATATGGATCCACTACAAATGCCATACTT  742

Human  867   GAAAGAGGAACCTTATTTTGGCATGGGGAAAATGGCAGTGAGCTGGCATCATGATGAAAA  926
             |||| | || ||||||||||  |||||||| ||||||||||| |||||||||||||| ||
Chick  743   GAAACAAGAGCCTTATTTTGAAATGGGGAACATGGCAGTGAGTTGGCATCATGATGAGAA  802

Human  927   TCTGGTGGACAGGTCAGCGGTGGCAGTGTACAGTTATAGCTGTGAAGGCCCTGAAGAGGA  986
             |||||| || |||||| | || || |||||||| || |||||||||||  |   ||  ||
Chick  803   TCTGGTTGAGAGGTCAACAGTTGCTGTGTACAGCTACAGCTGTGAAGGTTCATCAGCTGA  862

Human  987   AAGTGAGGATGACTCTCATCTCGAAGGCAGGGATCCTGATATTTGGCATGTTGGTTTTAA  1046
             |   |    ||| |   | ||  | || || || || | | |||||||||| || || ||
Chick  863   AGAAGCTACTGATTGGAACCTGAAGGGAAGAGACCCAGCTGTTTGGCATGTAGGCTTGAA  922

Human  1047  GATCTCATGGGACATAGAGACACCTGGTTTGGCGATACCCCTTCACCAAGGAGACTGCTA  1106
             | |  | |||||||||||||||||||| || || ||||| ||||||||||| |||  |||
Chick  923   GGTAGCGTGGGACATAGAGACACCTGGATTAGCAATACCACTTCACCAAGGCGACCTCTA  982

Human  1107  TTTCATGCTTGATGATCTCAATGCCACCCACCAACACTGTGTTTTGGCCGGTTCACAACC  1166
```

```
                 || ||||||||||||||||||    || ||||| ||||||||| |||| || |     |||
Chick  983    CTTGATGCTTGATGATCTCAATATGACACACCAGCACTGTGTTCTGGCTGGCTTTTCACC  1042

Human  1167   TCGGTTTAGTTCCACCCACCGAGTGGCAGAGTGCTCAACAGGAACCTTGGATTATATTTT  1226
              |||||| || ||||||||| |||||||||| || |||| |||||| ||||| || || ||
Chick  1043   TCGGTTCAGCTCCACCCACAGAGTGGCAGATTGTTCAAGAGGAACATTGGAATACATATT  1102

Human  1227   ACAACGCTGTCAGTTGGCTCTGCAGAATGT-----CTGTGACGATGTGGACAATGATGAT  1281
                  |  ||| |  |||| || |||||| |      |||    | |  |  |||      | |
Chick  1103   TGGGCAATGTGAACTGGCACTCCAGAATTTGCAAACTGATTCTAATTCAACA-----GCT  1157

Human  1282   GTCTCTTTGAAATCCTTTGAGCCTGCAGTTTTGAAACAAGGAGAAGAAATTCATAATGAG  1341
               | ||| |||||||   | ||   |||||||| | || |||    |||||||| |||||||||
Chick  1158   TTATCTCTGAAATCACTGGAAACTGCAGTTATAAAGCAAATGGAAGAAATACATAATGAG  1217

Human  1342   GTCGAGTTTGAGTGGCTGAGGCAGTTTTGGTTTCAAGGCAATCGATACAGAAAGTGCACT  1401
              || || ||||||||||| ||||||||||||||||||| | || ||     || ||||||
Chick  1218   GTTGAATTTGAGTGGCTTAGGCAGTTTTGGTTTCAAGGCGAGCGGTATTTGAAATGCACT  1277

Human  1402   GACTGGTGGTGTCAACCCATGGCTCAACTGGAAGCACTGTGGAAGAAGATGGAG  1455
              |||||||||    | | | || |||||| ||||||||| | | ||||   || ||||||
Chick  1278   GACTGGTGGCTTAAGCCTATGGCTAAACTGGAAGAATTTTGGAGAAAAATGGAG  1331
```

Range 2: 75 to 543GraphicsNext MatchPrevious MatchFirst Match

Alignment statistics for match #2

Score	Expect	Identities	Gaps	Strand
150 bits(166)	4e-39	326/480(68%)	16/480(3%)	Plus/Plus

```
Human  217   GGCAGCATGAAGCGCACCCCGACTGCCGAGGAACGAGAGCGCGAAGCTAAGAAACTGAGG  276
             |||||||||||| | |     ||   ||  | ||| || |||    |||    ||||||    | |
Chick  75    GGCAGCATGAAGAGGA----GA--GCAGGGGAGCGGGAGAAGGAACTGAAGAAAAAAAAG  128

Human  277   CTTCTTGAAGAGAGCTTGAAGACACTTGGCTCCCTTATCTGACCCCCAAAGATGATGAATTC  336
             |||||||||||||||| |||         || || || ||||| ||      |||| ||| ||
Chick  129   CTTCTTGAAGAGCTTGGAGAGGGCAAACTTCCATACCTGACACCAGCTGATGCTGATTT-  187

Human  337   TATCAGCAGTGGCAGCTG---AAATATCCTAAACTAATTCTCCGAGAAGCCAG-CAGTGT  392
               ||| || | ||||   |     | |||| | || |||||| || || ||| | |    |   ||
Chick  188   --TCATCATTTGCAGAAGACCAGATATTCCAAGCTAATTTTCAGA-AAGTCGGATACAGT  244

Human  393   ATCTGAGGAGCTCCATAAAGAGGTTCAAGAAGCCTTTCTCACACTGCACAAGCATGGCTG  452
             | |||| ||||||||| |     ||| ||||| | |||| | ||  ||     || ||||| ||
Chick  245   ACCTGAAGAGCTCCATCAGATGGTACAAGATGGCTTTTTGACCTTGAGGAAACATGGTTG  304

Human  453   CTTATTTCGGGACCTGGTTAGGATCCAAGGCAAAGATCTGCTCACTCCGGTATCTCGCAT  512
               || ||||   || || || |||||| |||| |||||| |    | || || || ||||| ||
Chick  305   TTTTTTTCAAGATCTTGTAAGGATCAAAGGAAAAGATTTTTTTACCCCAGTGTCTCGTAT  364

Human  513   CCTCATTGGTAATCCAGGCTGCACCTACAAGTACCTGAACACCAGGCTCTTTACGGTCCC  572
               | ||||| || ||||| ||||| ||||||||| ||||||| ||   | ||||| || ||
Chick  365   ATTAATTGGAAAGCCAGGATGCACTTACAAGTACTTGAACACAAGATTATTTACAGTTCC  424

Human  573   CTGGCC-AGTGAAAGGGTCTAATATAAAACACACCGAGGCTGAAATAGCCGCTGCTTGTG  631
               ||||| | || | || | | | |||||| |    |     || |||||    | |||||||
Chick  425   TTGGCCTACTG-AGGGTTGTGAAATAAAATATTGCAGTCCTCAAATACATGATGCTTGTA  483

Human  632   AGACCTTCCTCAAGCTCAATGACTACCTGCAGATAGAAACCATCCAGGCTTTGGAAGAAC  691
             |   | ||   |||| || ||||||||| |||| || ||| |   || |||| ||   ||| ||
Chick  484   AAGCATTAATCAAACTTAATGACTACTTGCATATTGAAGCAGTCAAGGCATTACAAGGAC  543
```

Range 3: 1497 to 1564GraphicsNext MatchPrevious MatchFirst Match

Alignment statistics for match #3

Score	Expect	Identities	Gaps	Strand
38.3 bits(41)	3e-05	49/68(72%)	0/68(0%)	Plus/Plus

```
Human  1684   GATCAGAAGCCAGAATGTCGGCCATACTGGGAAAAGGATGATGCTTCGATGCCTCTGCCG  1743
              || |||||||||||||| |   || | ||||   ||| || ||    |    |||||||||||
Chick  1497   GAGCAGAAGCCAGAATGCCATCCCTTCTGGACAAATGAAGAAAGTAACATGCCTCTGCCA  1556
```

```
Human  1744  TTTGACCT  1751
              |||| ||
Chick  1557  CTTGATCT  1564
```

Range 4: 1347 to 1398[Graphics](#)Next MatchPrevious Match[First Match](#)

Alignment statistics for match #4

Score	Expect	Identities	Gaps	Strand
31.9 bits(34)	0.001	38/52(73%)	0/52(0%)	Plus/Plus

```
Human  1534  GTGCTTCATGAAGTTAAAAGAGAGGGGCTCCCCGTGGAACAAAGGAATGAAA  1585
             || || || ||||||  || | ||| ||   | |  ||||||||||||||||
Chick  1347  GTCCTCCAAGAAGTTCGAAAAAAGGAGCAAACTGAAGAACAAAGGAATGAAA  1398
```
