# Supplementary material for: Nucleotide Composition of Ultra-Conserved Elements Shows Excess of GpC and Depletion of GG and CC Dinucleotides
Source: Genes (Basel). 2022 Nov 7;13(11):2053. doi: 10.3390/genes13112053 (PMC9690913; doi:10.3390/genes13112053)

Figure S2. Number of alternative alleles with the frequencies up to 2% inside the 4271 UCNE sequences among 2504 individuals from five regions. Individuals are represented in five groups depending on their ethnicity according to their classification in the 1000 Genomes Database. AFR represents African populations (navy blue), AMR – Americans populations (red), EAS - East Asian (purple), EUR – Europeans (green), and SAS – South Asia (yellow). Each individual is represented by a colored bar, which position along horizontal axis corresponds to the total number of alternative alleles inside UCNEs in this person.

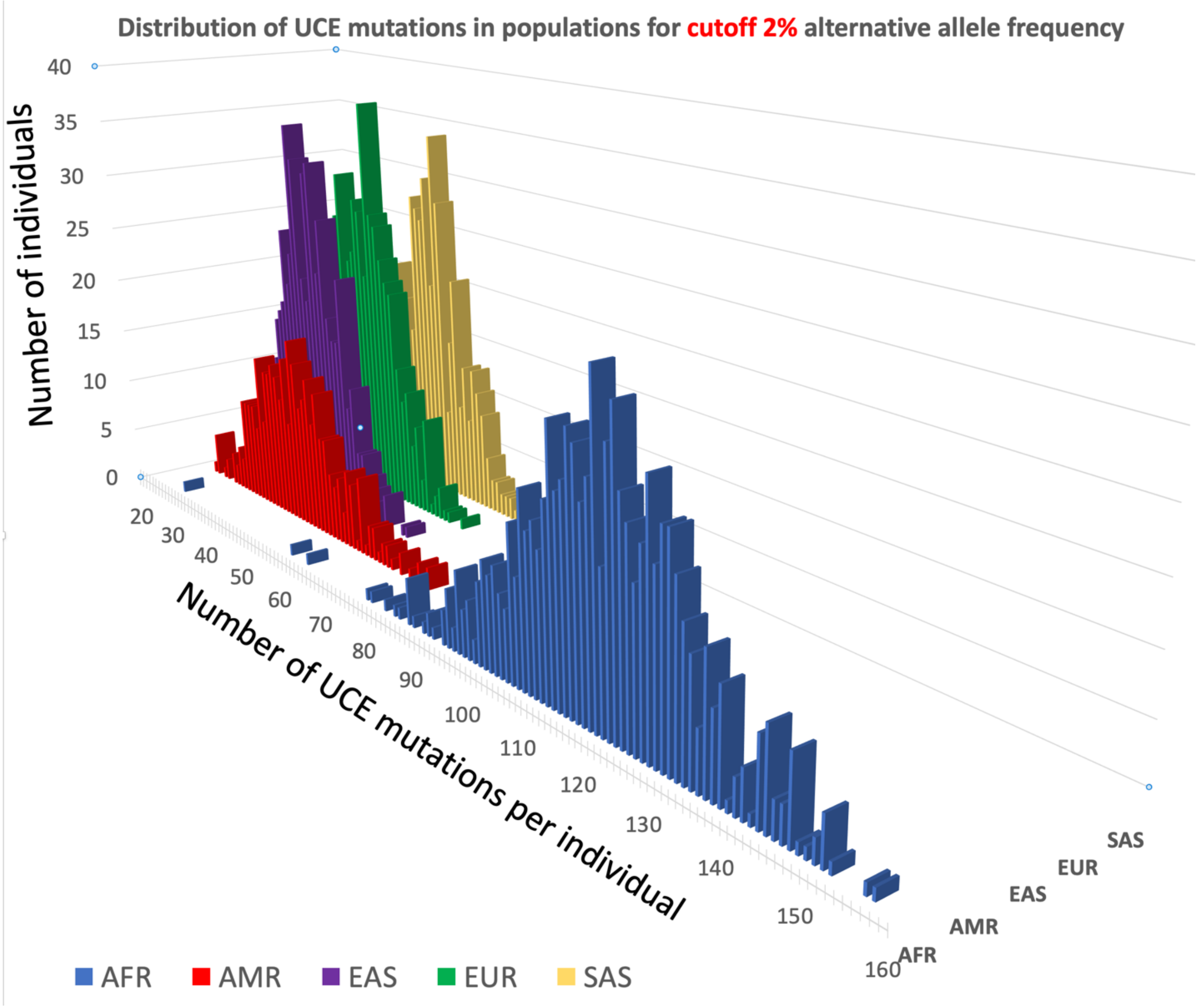

Supplement: Supplementary file 1 [file genes-13-02053-s001.zip › Figure S2.pdf]
