# Supplementary material for: Nucleotide Composition of Ultra-Conserved Elements Shows Excess of GpC and Depletion of GG and CC Dinucleotides
Source: Genes (Basel). 2022 Nov 7;13(11):2053. doi: 10.3390/genes13112053 (PMC9690913; doi:10.3390/genes13112053)
Supplement: Supplementary file 1 [file genes-13-02053-s001.zip › Table S1.pdf]

Supplementary Table S1.

Table S1. Distribution of SNPs by their alternative allele frequencies inside UCN **whole genome.** SNPs are divided into one hundred bins by their alternative allel shown in column one. Columns 2 and 4 show the number of SNPs in the corresp inside the whole genome and UCNEs respectively. Columns 3 and 5 show the rel frequencies of SNPs in the bins by dividing the number of SNPs in the bin by the analyzed SNPs in the whole genome and UCNEs respectively.

Alternative allele frequency frequency	WHOLE GENOME		ULTRA CONSERVEI
	**Number of SNPs inside whole genome**	**Relative frequency (%) of SNPs inside whole genome**	**Number of SNPs inside UCEs**
0	68430653	84.438	28787
1	2709034	3.343	632
2	1249017	1.541	280
3	761505	0.940	154
4	536314	0.662	93
5	411115	0.507	69
6	334473	0.413	78
7	287678	0.355	59
8	258931	0.320	52
9	231334	0.285	39
10	213325	0.263	31
11	193665	0.239	40
12	187021	0.231	35
13	170933	0.211	24
14	159373	0.197	30
15	148977	0.184	17
16	143358	0.177	26
17	138137	0.170	18
18	127266	0.157	20
19	124591	0.154	22
20	121181	0.150	11
21	115206	0.142	18
22	109608	0.135	17
23	105165	0.130	14
24	102292	0.126	15
25	100997	0.125	9

26	96182	0.119	13
27	91455	0.113	10
28	89618	0.111	10
29	86269	0.106	10
30	83876	0.103	6
31	82189	0.101	10
32	80153	0.099	8
33	77799	0.096	12
34	74583	0.092	4
35	73796	0.091	12
36	70952	0.088	13
37	71864	0.089	6
38	68089	0.084	11
39	66173	0.082	8
40	64815	0.080	10
41	62487	0.077	11
42	60880	0.075	6
43	60071	0.074	4
44	59409	0.073	8
45	58713	0.072	4
46	58015	0.072	5
47	55234	0.068	4
48	53698	0.066	4
49	52092	0.064	10
50	51571	0.064	10
51	50934	0.063	3
52	50371	0.062	5
53	49384	0.061	5
54	48306	0.060	5
55	46749	0.058	6
56	45103	0.056	5
57	46595	0.057	8
58	45664	0.056	8
59	43559	0.054	6
60	43936	0.054	7
61	41682	0.051	2
62	40851	0.050	4
63	40460	0.050	8
64	39923	0.049	7
65	39292	0.048	3
66	38521	0.048	4
67	38202	0.047	1
68	38035	0.047	5
69	37972	0.047	3
70	36353	0.045	3

71	36213	0.045	4
72	34718	0.043	7
73	33854	0.042	3
74	33556	0.041	0
75	34607	0.043	3
76	33635	0.042	3
77	31792	0.039	6
78	32434	0.040	4
79	31378	0.039	4
80	31589	0.039	4
81	29781	0.037	6
82	30140	0.037	4
83	28891	0.036	5
84	28460	0.035	2
85	28535	0.035	4
86	27017	0.033	7
87	27895	0.034	8
88	26204	0.032	6
89	25613	0.032	3
90	25633	0.032	5
91	25054	0.031	2
92	25257	0.031	3
93	25467	0.031	4
94	26302	0.032	3
95	28613	0.035	6
96	32384	0.040	5
97	38774	0.048	4
98	49644	0.061	12
99	72866	0.090	21
100	22942	0.028	1

NEs and the
le frequencies
ponding bin
lative
total number of

D ELEMENTS ONLY
**Relative frequency (%) of SNPs inside UCEs**
92.724
2.036
0.902
0.496
0.300
0.222
0.251
0.190
0.167
0.126
0.100
0.129
0.113
0.077
0.097
0.055
0.084
0.058
0.064
0.071
0.035
0.058
0.055
0.045
0.048
0.029

0.042
0.032
0.032
0.032
0.019
0.032
0.026
0.039
0.013
0.039
0.042
0.019
0.035
0.026
0.032
0.035
0.019
0.013
0.026
0.013
0.016
0.013
0.013
0.032
0.032
0.010
0.016
0.016
0.016
0.019
0.016
0.026
0.026
0.019
0.023
0.006
0.013
0.026
0.023
0.010
0.013
0.003
0.016
0.010
0.010

0.013
0.023
0.010
0.000
0.010
0.010
0.019
0.013
0.013
0.013
0.019
0.013
0.016
0.006
0.013
0.023
0.026
0.019
0.010
0.016
0.006
0.010
0.013
0.010
0.019
0.016
0.013
0.039
0.068
0.003
