# Supplementary material for: Nucleotide Composition of Ultra-Conserved Elements Shows Excess of GpC and Depletion of GG and CC Dinucleotides
Source: Genes (Basel). 2022 Nov 7;13(11):2053. doi: 10.3390/genes13112053 (PMC9690913; doi:10.3390/genes13112053)
Supplement: Supplementary file 1 [file genes-13-02053-s001.zip › Table S2.pdf]

Supplementary Table S2
Column 1: Identifiers of individuals from 1000 Genomes database
Column 2: Population identifiers from 1000 Genomes database
Column 3: Region identifiers from 1000 Genomes
Column 4: Total counts of alternative alleles inside 4271 UCNE for the corresponding individual

HG00096	GBR	EUR	349
HG00097	GBR	EUR	366
HG00099	GBR	EUR	373
HG00100	GBR	EUR	360
HG00101	GBR	EUR	360
HG00102	GBR	EUR	348
HG00103	GBR	EUR	350
HG00105	GBR	EUR	347
HG00106	GBR	EUR	339
HG00107	GBR	EUR	337
HG00108	GBR	EUR	366
HG00109	GBR	EUR	357
HG00110	GBR	EUR	337
HG00111	GBR	EUR	350
HG00112	GBR	EUR	344
HG00113	GBR	EUR	344
HG00114	GBR	EUR	331
HG00115	GBR	EUR	318
HG00116	GBR	EUR	352
HG00117	GBR	EUR	339
HG00118	GBR	EUR	335
HG00119	GBR	EUR	355
HG00120	GBR	EUR	340
HG00121	GBR	EUR	380
HG00122	GBR	EUR	339
HG00123	GBR	EUR	360
HG00125	GBR	EUR	347
HG00126	GBR	EUR	356
HG00127	GBR	EUR	360
HG00128	GBR	EUR	340
HG00129	GBR	EUR	358
HG00130	GBR	EUR	376
HG00131	GBR	EUR	348
HG00132	GBR	EUR	342
HG00133	GBR	EUR	363
HG00136	GBR	EUR	347
HG00137	GBR	EUR	345
HG00138	GBR	EUR	341
HG00139	GBR	EUR	363
HG00140	GBR	EUR	371
HG00141	GBR	EUR	380
HG00142	GBR	EUR	329
HG00143	GBR	EUR	351

HG00145	GBR	EUR	326
HG00146	GBR	EUR	354
HG00148	GBR	EUR	326
HG00149	GBR	EUR	368
HG00150	GBR	EUR	341
HG00151	GBR	EUR	362
HG00154	GBR	EUR	349
HG00155	GBR	EUR	346
HG00157	GBR	EUR	337
HG00158	GBR	EUR	331
HG00159	GBR	EUR	368
HG00160	GBR	EUR	343
HG00171	FIN	EUR	345
HG00173	FIN	EUR	363
HG00174	FIN	EUR	343
HG00176	FIN	EUR	357
HG00177	FIN	EUR	316
HG00178	FIN	EUR	372
HG00179	FIN	EUR	299
HG00180	FIN	EUR	336
HG00181	FIN	EUR	338
HG00182	FIN	EUR	336
HG00183	FIN	EUR	323
HG00185	FIN	EUR	329
HG00186	FIN	EUR	309
HG00187	FIN	EUR	379
HG00188	FIN	EUR	356
HG00189	FIN	EUR	320
HG00190	FIN	EUR	339
HG00231	GBR	EUR	354
HG00232	GBR	EUR	375
HG00233	GBR	EUR	355
HG00234	GBR	EUR	373
HG00235	GBR	EUR	362
HG00236	GBR	EUR	361
HG00237	GBR	EUR	351
HG00238	GBR	EUR	383
HG00239	GBR	EUR	353
HG00240	GBR	EUR	371
HG00242	GBR	EUR	364
HG00243	GBR	EUR	336
HG00244	GBR	EUR	351
HG00245	GBR	EUR	356
HG00246	GBR	EUR	360
HG00250	GBR	EUR	374
HG00251	GBR	EUR	350
HG00252	GBR	EUR	326
HG00253	GBR	EUR	357
HG00254	GBR	EUR	346
HG00255	GBR	EUR	386

HG00256	GBR	EUR	339
HG00257	GBR	EUR	348
HG00258	GBR	EUR	346
HG00259	GBR	EUR	340
HG00260	GBR	EUR	353
HG00261	GBR	EUR	347
HG00262	GBR	EUR	355
HG00263	GBR	EUR	312
HG00264	GBR	EUR	353
HG00265	GBR	EUR	355
HG00266	FIN	EUR	370
HG00267	FIN	EUR	348
HG00268	FIN	EUR	327
HG00269	FIN	EUR	323
HG00271	FIN	EUR	340
HG00272	FIN	EUR	344
HG00273	FIN	EUR	384
HG00274	FIN	EUR	308
HG00275	FIN	EUR	354
HG00276	FIN	EUR	345
HG00277	FIN	EUR	328
HG00278	FIN	EUR	343
HG00280	FIN	EUR	372
HG00281	FIN	EUR	386
HG00282	FIN	EUR	346
HG00284	FIN	EUR	353
HG00285	FIN	EUR	344
HG00288	FIN	EUR	357
HG00290	FIN	EUR	341
HG00304	FIN	EUR	371
HG00306	FIN	EUR	350
HG00308	FIN	EUR	311
HG00309	FIN	EUR	351
HG00310	FIN	EUR	358
HG00311	FIN	EUR	326
HG00313	FIN	EUR	332
HG00315	FIN	EUR	353
HG00318	FIN	EUR	340
HG00319	FIN	EUR	353
HG00320	FIN	EUR	340
HG00321	FIN	EUR	353
HG00323	FIN	EUR	316
HG00324	FIN	EUR	338
HG00325	FIN	EUR	363
HG00326	FIN	EUR	339
HG00327	FIN	EUR	336
HG00328	FIN	EUR	388
HG00329	FIN	EUR	332
HG00330	FIN	EUR	345
HG00331	FIN	EUR	357

HG00332	FIN	EUR	332
HG00334	FIN	EUR	348
HG00335	FIN	EUR	355
HG00336	FIN	EUR	381
HG00337	FIN	EUR	338
HG00338	FIN	EUR	332
HG00339	FIN	EUR	358
HG00341	FIN	EUR	367
HG00342	FIN	EUR	349
HG00343	FIN	EUR	343
HG00344	FIN	EUR	351
HG00345	FIN	EUR	333
HG00346	FIN	EUR	352
HG00349	FIN	EUR	375
HG00350	FIN	EUR	356
HG00351	FIN	EUR	346
HG00353	FIN	EUR	331
HG00355	FIN	EUR	338
HG00356	FIN	EUR	337
HG00357	FIN	EUR	349
HG00358	FIN	EUR	363
HG00360	FIN	EUR	343
HG00361	FIN	EUR	332
HG00362	FIN	EUR	339
HG00364	FIN	EUR	374
HG00365	FIN	EUR	323
HG00366	FIN	EUR	331
HG00367	FIN	EUR	374
HG00368	FIN	EUR	330
HG00369	FIN	EUR	363
HG00371	FIN	EUR	362
HG00372	FIN	EUR	349
HG00373	FIN	EUR	361
HG00375	FIN	EUR	335
HG00376	FIN	EUR	358
HG00378	FIN	EUR	371
HG00379	FIN	EUR	381
HG00380	FIN	EUR	326
HG00381	FIN	EUR	367
HG00382	FIN	EUR	357
HG00383	FIN	EUR	344
HG00384	FIN	EUR	363
HG00403	CHS	EAS	360
HG00404	CHS	EAS	382
HG00406	CHS	EAS	390
HG00407	CHS	EAS	364
HG00409	CHS	EAS	359
HG00410	CHS	EAS	369
HG00419	CHS	EAS	383
HG00421	CHS	EAS	378

HG00422	CHS	EAS	402
HG00428	CHS	EAS	358
HG00436	CHS	EAS	384
HG00437	CHS	EAS	390
HG00442	CHS	EAS	356
HG00443	CHS	EAS	383
HG00445	CHS	EAS	390
HG00446	CHS	EAS	389
HG00448	CHS	EAS	394
HG00449	CHS	EAS	385
HG00451	CHS	EAS	348
HG00452	CHS	EAS	405
HG00457	CHS	EAS	398
HG00458	CHS	EAS	363
HG00463	CHS	EAS	369
HG00464	CHS	EAS	382
HG00472	CHS	EAS	398
HG00473	CHS	EAS	369
HG00475	CHS	EAS	391
HG00476	CHS	EAS	362
HG00478	CHS	EAS	375
HG00479	CHS	EAS	373
HG00500	CHS	EAS	350
HG00513	CHS	EAS	390
HG00524	CHS	EAS	385
HG00525	CHS	EAS	379
HG00530	CHS	EAS	376
HG00531	CHS	EAS	390
HG00533	CHS	EAS	338
HG00534	CHS	EAS	365
HG00536	CHS	EAS	375
HG00537	CHS	EAS	370
HG00542	CHS	EAS	372
HG00543	CHS	EAS	382
HG00551	PUR	AMR	372
HG00553	PUR	AMR	369
HG00554	PUR	AMR	373
HG00556	CHS	EAS	398
HG00557	CHS	EAS	374
HG00559	CHS	EAS	353
HG00560	CHS	EAS	356
HG00565	CHS	EAS	352
HG00566	CHS	EAS	356
HG00580	CHS	EAS	397
HG00581	CHS	EAS	371
HG00583	CHS	EAS	355
HG00584	CHS	EAS	374
HG00589	CHS	EAS	372
HG00590	CHS	EAS	360
HG00592	CHS	EAS	363

HG00593	CHS	EAS	371
HG00595	CHS	EAS	393
HG00596	CHS	EAS	370
HG00598	CHS	EAS	372
HG00599	CHS	EAS	380
HG00607	CHS	EAS	353
HG00608	CHS	EAS	390
HG00610	CHS	EAS	364
HG00611	CHS	EAS	357
HG00613	CHS	EAS	380
HG00614	CHS	EAS	373
HG00619	CHS	EAS	328
HG00620	CHS	EAS	348
HG00622	CHS	EAS	363
HG00623	CHS	EAS	355
HG00625	CHS	EAS	349
HG00626	CHS	EAS	398
HG00628	CHS	EAS	355
HG00629	CHS	EAS	383
HG00631	CHS	EAS	389
HG00632	CHS	EAS	352
HG00634	CHS	EAS	357
HG00637	PUR	AMR	356
HG00638	PUR	AMR	389
HG00640	PUR	AMR	369
HG00641	PUR	AMR	404
HG00650	CHS	EAS	352
HG00651	CHS	EAS	365
HG00653	CHS	EAS	396
HG00654	CHS	EAS	407
HG00656	CHS	EAS	357
HG00657	CHS	EAS	356
HG00662	CHS	EAS	401
HG00663	CHS	EAS	397
HG00671	CHS	EAS	368
HG00672	CHS	EAS	375
HG00674	CHS	EAS	391
HG00675	CHS	EAS	363
HG00683	CHS	EAS	356
HG00684	CHS	EAS	356
HG00689	CHS	EAS	365
HG00690	CHS	EAS	339
HG00692	CHS	EAS	395
HG00693	CHS	EAS	370
HG00698	CHS	EAS	375
HG00699	CHS	EAS	358
HG00701	CHS	EAS	389
HG00704	CHS	EAS	363
HG00705	CHS	EAS	368
HG00707	CHS	EAS	365

HG00708	CHS	EAS	421
HG00717	CHS	EAS	364
HG00728	CHS	EAS	373
HG00729	CHS	EAS	369
HG00731	PUR	AMR	358
HG00732	PUR	AMR	359
HG00734	PUR	AMR	365
HG00736	PUR	AMR	367
HG00737	PUR	AMR	355
HG00739	PUR	AMR	408
HG00740	PUR	AMR	364
HG00742	PUR	AMR	344
HG00743	PUR	AMR	363
HG00759	CDX	EAS	361
HG00766	CDX	EAS	345
HG00844	CDX	EAS	355
HG00851	CDX	EAS	374
HG00864	CDX	EAS	363
HG00867	CDX	EAS	352
HG00879	CDX	EAS	334
HG00881	CDX	EAS	399
HG00956	CDX	EAS	371
HG00978	CDX	EAS	357
HG00982	CDX	EAS	353
HG01028	CDX	EAS	354
HG01029	CDX	EAS	366
HG01031	CDX	EAS	373
HG01046	CDX	EAS	374
HG01047	PUR	AMR	368
HG01048	PUR	AMR	390
HG01049	PUR	AMR	364
HG01051	PUR	AMR	364
HG01052	PUR	AMR	365
HG01054	PUR	AMR	381
HG01055	PUR	AMR	384
HG01058	PUR	AMR	390
HG01060	PUR	AMR	366
HG01061	PUR	AMR	383
HG01063	PUR	AMR	400
HG01064	PUR	AMR	392
HG01066	PUR	AMR	343
HG01067	PUR	AMR	354
HG01069	PUR	AMR	388
HG01070	PUR	AMR	381
HG01072	PUR	AMR	355
HG01073	PUR	AMR	357
HG01075	PUR	AMR	372
HG01077	PUR	AMR	405
HG01079	PUR	AMR	399
HG01080	PUR	AMR	375

HG01082	PUR	AMR	394
HG01083	PUR	AMR	349
HG01085	PUR	AMR	369
HG01086	PUR	AMR	387
HG01088	PUR	AMR	356
HG01089	PUR	AMR	387
HG01092	PUR	AMR	390
HG01094	PUR	AMR	369
HG01095	PUR	AMR	381
HG01097	PUR	AMR	383
HG01098	PUR	AMR	368
HG01101	PUR	AMR	339
HG01102	PUR	AMR	404
HG01104	PUR	AMR	395
HG01105	PUR	AMR	362
HG01107	PUR	AMR	391
HG01108	PUR	AMR	428
HG01110	PUR	AMR	395
HG01111	PUR	AMR	386
HG01112	CLM	AMR	370
HG01113	CLM	AMR	356
HG01119	CLM	AMR	385
HG01121	CLM	AMR	348
HG01122	CLM	AMR	339
HG01124	CLM	AMR	344
HG01125	CLM	AMR	388
HG01130	CLM	AMR	369
HG01131	CLM	AMR	377
HG01133	CLM	AMR	378
HG01134	CLM	AMR	373
HG01136	CLM	AMR	378
HG01137	CLM	AMR	347
HG01139	CLM	AMR	380
HG01140	CLM	AMR	349
HG01142	CLM	AMR	403
HG01148	CLM	AMR	362
HG01149	CLM	AMR	378
HG01161	PUR	AMR	375
HG01162	PUR	AMR	413
HG01164	PUR	AMR	389
HG01167	PUR	AMR	403
HG01168	PUR	AMR	393
HG01170	PUR	AMR	369
HG01171	PUR	AMR	377
HG01173	PUR	AMR	348
HG01174	PUR	AMR	377
HG01176	PUR	AMR	374
HG01177	PUR	AMR	369
HG01182	PUR	AMR	349
HG01183	PUR	AMR	371

HG01187	PUR	AMR	377
HG01188	PUR	AMR	387
HG01190	PUR	AMR	413
HG01191	PUR	AMR	378
HG01197	PUR	AMR	387
HG01198	PUR	AMR	369
HG01200	PUR	AMR	339
HG01204	PUR	AMR	364
HG01205	PUR	AMR	364
HG01241	PUR	AMR	475
HG01242	PUR	AMR	402
HG01247	PUR	AMR	395
HG01248	PUR	AMR	368
HG01250	CLM	AMR	385
HG01251	CLM	AMR	363
HG01253	CLM	AMR	343
HG01254	CLM	AMR	370
HG01256	CLM	AMR	324
HG01257	CLM	AMR	338
HG01259	CLM	AMR	349
HG01260	CLM	AMR	394
HG01269	CLM	AMR	342
HG01271	CLM	AMR	372
HG01272	CLM	AMR	375
HG01275	CLM	AMR	359
HG01277	CLM	AMR	346
HG01280	CLM	AMR	377
HG01281	CLM	AMR	387
HG01284	CLM	AMR	391
HG01286	PUR	AMR	380
HG01302	PUR	AMR	345
HG01303	PUR	AMR	370
HG01305	PUR	AMR	412
HG01308	PUR	AMR	392
HG01311	PUR	AMR	371
HG01312	PUR	AMR	395
HG01323	PUR	AMR	347
HG01325	PUR	AMR	402
HG01326	PUR	AMR	362
HG01334	GBR	EUR	374
HG01341	CLM	AMR	362
HG01342	CLM	AMR	430
HG01344	CLM	AMR	373
HG01345	CLM	AMR	363
HG01348	CLM	AMR	363
HG01350	CLM	AMR	357
HG01351	CLM	AMR	354
HG01353	CLM	AMR	348
HG01354	CLM	AMR	344
HG01356	CLM	AMR	392

HG01357	CLM	AMR	386
HG01359	CLM	AMR	338
HG01360	CLM	AMR	321
HG01362	CLM	AMR	372
HG01363	CLM	AMR	382
HG01365	CLM	AMR	347
HG01366	CLM	AMR	387
HG01369	CLM	AMR	359
HG01372	CLM	AMR	365
HG01374	CLM	AMR	345
HG01375	CLM	AMR	377
HG01377	CLM	AMR	365
HG01378	CLM	AMR	363
HG01383	CLM	AMR	345
HG01384	CLM	AMR	363
HG01389	CLM	AMR	377
HG01390	CLM	AMR	387
HG01392	PUR	AMR	356
HG01393	PUR	AMR	362
HG01395	PUR	AMR	382
HG01396	PUR	AMR	355
HG01398	PUR	AMR	384
HG01402	PUR	AMR	385
HG01403	PUR	AMR	371
HG01405	PUR	AMR	393
HG01412	PUR	AMR	410
HG01413	PUR	AMR	376
HG01414	PUR	AMR	366
HG01431	CLM	AMR	363
HG01432	CLM	AMR	322
HG01435	CLM	AMR	361
HG01437	CLM	AMR	346
HG01438	CLM	AMR	362
HG01440	CLM	AMR	362
HG01441	CLM	AMR	350
HG01443	CLM	AMR	355
HG01444	CLM	AMR	386
HG01447	CLM	AMR	383
HG01455	CLM	AMR	381
HG01456	CLM	AMR	357
HG01459	CLM	AMR	375
HG01461	CLM	AMR	390
HG01462	CLM	AMR	406
HG01464	CLM	AMR	379
HG01465	CLM	AMR	347
HG01468	CLM	AMR	324
HG01474	CLM	AMR	362
HG01479	CLM	AMR	373
HG01485	CLM	AMR	412
HG01486	CLM	AMR	368

HG01488	CLM	AMR	417
HG01489	CLM	AMR	361
HG01491	CLM	AMR	374
HG01492	CLM	AMR	337
HG01494	CLM	AMR	367
HG01495	CLM	AMR	365
HG01497	CLM	AMR	361
HG01498	CLM	AMR	333
HG01500	IBS	EUR	382
HG01501	IBS	EUR	366
HG01503	IBS	EUR	355
HG01504	IBS	EUR	363
HG01506	IBS	EUR	375
HG01507	IBS	EUR	325
HG01509	IBS	EUR	376
HG01510	IBS	EUR	343
HG01512	IBS	EUR	323
HG01513	IBS	EUR	341
HG01515	IBS	EUR	310
HG01516	IBS	EUR	360
HG01518	IBS	EUR	366
HG01519	IBS	EUR	354
HG01521	IBS	EUR	356
HG01522	IBS	EUR	346
HG01524	IBS	EUR	355
HG01525	IBS	EUR	352
HG01527	IBS	EUR	344
HG01528	IBS	EUR	341
HG01530	IBS	EUR	346
HG01531	IBS	EUR	340
HG01536	IBS	EUR	337
HG01537	IBS	EUR	370
HG01550	CLM	AMR	365
HG01551	CLM	AMR	396
HG01556	CLM	AMR	364
HG01565	PEL	AMR	366
HG01566	PEL	AMR	354
HG01571	PEL	AMR	372
HG01572	PEL	AMR	370
HG01577	PEL	AMR	391
HG01578	PEL	AMR	364
HG01583	PJL	SAS	378
HG01586	PJL	SAS	352
HG01589	PJL	SAS	390
HG01593	PJL	SAS	372
HG01595	KHV	EAS	348
HG01596	KHV	EAS	375
HG01597	KHV	EAS	358
HG01598	KHV	EAS	364
HG01599	KHV	EAS	375

HG01600	KHV	EAS	345
HG01602	IBS	EUR	367
HG01603	IBS	EUR	346
HG01605	IBS	EUR	361
HG01606	IBS	EUR	333
HG01607	IBS	EUR	388
HG01608	IBS	EUR	360
HG01610	IBS	EUR	339
HG01612	IBS	EUR	365
HG01613	IBS	EUR	341
HG01615	IBS	EUR	349
HG01617	IBS	EUR	369
HG01618	IBS	EUR	343
HG01619	IBS	EUR	345
HG01620	IBS	EUR	390
HG01623	IBS	EUR	351
HG01624	IBS	EUR	342
HG01625	IBS	EUR	356
HG01626	IBS	EUR	343
HG01628	IBS	EUR	336
HG01630	IBS	EUR	384
HG01631	IBS	EUR	363
HG01632	IBS	EUR	322
HG01668	IBS	EUR	345
HG01669	IBS	EUR	349
HG01670	IBS	EUR	329
HG01672	IBS	EUR	381
HG01673	IBS	EUR	370
HG01675	IBS	EUR	340
HG01676	IBS	EUR	365
HG01678	IBS	EUR	378
HG01679	IBS	EUR	336
HG01680	IBS	EUR	361
HG01682	IBS	EUR	335
HG01684	IBS	EUR	359
HG01685	IBS	EUR	350
HG01686	IBS	EUR	353
HG01694	IBS	EUR	362
HG01695	IBS	EUR	352
HG01697	IBS	EUR	352
HG01699	IBS	EUR	368
HG01700	IBS	EUR	357
HG01702	IBS	EUR	369
HG01704	IBS	EUR	334
HG01705	IBS	EUR	349
HG01707	IBS	EUR	341
HG01708	IBS	EUR	372
HG01709	IBS	EUR	338
HG01710	IBS	EUR	349
HG01746	IBS	EUR	366

HG01747	IBS	EUR	357
HG01756	IBS	EUR	331
HG01757	IBS	EUR	365
HG01761	IBS	EUR	358
HG01762	IBS	EUR	329
HG01765	IBS	EUR	365
HG01766	IBS	EUR	362
HG01767	IBS	EUR	366
HG01768	IBS	EUR	356
HG01770	IBS	EUR	364
HG01771	IBS	EUR	376
HG01773	IBS	EUR	375
HG01775	IBS	EUR	316
HG01776	IBS	EUR	363
HG01777	IBS	EUR	324
HG01779	IBS	EUR	352
HG01781	IBS	EUR	370
HG01783	IBS	EUR	366
HG01784	IBS	EUR	363
HG01785	IBS	EUR	343
HG01786	IBS	EUR	367
HG01789	GBR	EUR	356
HG01790	GBR	EUR	320
HG01791	GBR	EUR	342
HG01794	CDX	EAS	361
HG01795	CDX	EAS	386
HG01796	CDX	EAS	385
HG01797	CDX	EAS	352
HG01798	CDX	EAS	375
HG01799	CDX	EAS	372
HG01800	CDX	EAS	367
HG01801	CDX	EAS	375
HG01802	CDX	EAS	404
HG01804	CDX	EAS	406
HG01805	CDX	EAS	377
HG01806	CDX	EAS	368
HG01807	CDX	EAS	331
HG01808	CDX	EAS	344
HG01809	CDX	EAS	376
HG01810	CDX	EAS	366
HG01811	CDX	EAS	378
HG01812	CDX	EAS	382
HG01813	CDX	EAS	405
HG01815	CDX	EAS	364
HG01816	CDX	EAS	362
HG01817	CDX	EAS	368
HG01840	KHV	EAS	386
HG01841	KHV	EAS	361
HG01842	KHV	EAS	356
HG01843	KHV	EAS	380

HG01844	KHV	EAS	405
HG01845	KHV	EAS	378
HG01846	KHV	EAS	341
HG01847	KHV	EAS	360
HG01848	KHV	EAS	412
HG01849	KHV	EAS	371
HG01850	KHV	EAS	380
HG01851	KHV	EAS	377
HG01852	KHV	EAS	368
HG01853	KHV	EAS	372
HG01855	KHV	EAS	396
HG01857	KHV	EAS	365
HG01858	KHV	EAS	366
HG01859	KHV	EAS	385
HG01860	KHV	EAS	353
HG01861	KHV	EAS	355
HG01862	KHV	EAS	388
HG01863	KHV	EAS	397
HG01864	KHV	EAS	366
HG01865	KHV	EAS	396
HG01866	KHV	EAS	351
HG01867	KHV	EAS	371
HG01868	KHV	EAS	371
HG01869	KHV	EAS	376
HG01870	KHV	EAS	399
HG01871	KHV	EAS	377
HG01872	KHV	EAS	372
HG01873	KHV	EAS	393
HG01874	KHV	EAS	361
HG01878	KHV	EAS	384
HG01879	ACB	AFR	438
HG01880	ACB	AFR	374
HG01882	ACB	AFR	477
HG01883	ACB	AFR	459
HG01885	ACB	AFR	526
HG01886	ACB	AFR	468
HG01889	ACB	AFR	456
HG01890	ACB	AFR	476
HG01892	PEL	AMR	399
HG01893	PEL	AMR	395
HG01894	ACB	AFR	463
HG01896	ACB	AFR	475
HG01912	ACB	AFR	516
HG01914	ACB	AFR	462
HG01915	ACB	AFR	507
HG01917	PEL	AMR	366
HG01918	PEL	AMR	386
HG01920	PEL	AMR	339
HG01921	PEL	AMR	348
HG01923	PEL	AMR	370

HG01924	PEL	AMR	404
HG01926	PEL	AMR	399
HG01927	PEL	AMR	342
HG01932	PEL	AMR	340
HG01933	PEL	AMR	379
HG01935	PEL	AMR	348
HG01936	PEL	AMR	376
HG01938	PEL	AMR	347
HG01939	PEL	AMR	363
HG01941	PEL	AMR	372
HG01942	PEL	AMR	379
HG01944	PEL	AMR	353
HG01945	PEL	AMR	356
HG01947	PEL	AMR	382
HG01948	PEL	AMR	415
HG01950	PEL	AMR	350
HG01951	PEL	AMR	366
HG01953	PEL	AMR	359
HG01954	PEL	AMR	378
HG01956	ACB	AFR	445
HG01958	ACB	AFR	477
HG01961	PEL	AMR	360
HG01965	PEL	AMR	387
HG01967	PEL	AMR	393
HG01968	PEL	AMR	388
HG01970	PEL	AMR	358
HG01971	PEL	AMR	409
HG01973	PEL	AMR	370
HG01974	PEL	AMR	366
HG01976	PEL	AMR	339
HG01977	PEL	AMR	393
HG01979	PEL	AMR	370
HG01980	PEL	AMR	396
HG01982	PEL	AMR	380
HG01985	ACB	AFR	496
HG01986	ACB	AFR	460
HG01988	ACB	AFR	424
HG01989	ACB	AFR	447
HG01990	ACB	AFR	456
HG01991	PEL	AMR	354
HG01992	PEL	AMR	351
HG01997	PEL	AMR	370
HG02002	PEL	AMR	360
HG02003	PEL	AMR	368
HG02006	PEL	AMR	415
HG02008	PEL	AMR	342
HG02009	ACB	AFR	466
HG02010	ACB	AFR	444
HG02012	ACB	AFR	476
HG02013	ACB	AFR	467

HG02014	ACB	AFR	489
HG02016	KHV	EAS	375
HG02017	KHV	EAS	363
HG02019	KHV	EAS	366
HG02020	KHV	EAS	361
HG02023	KHV	EAS	372
HG02025	KHV	EAS	356
HG02026	KHV	EAS	376
HG02028	KHV	EAS	349
HG02029	KHV	EAS	351
HG02031	KHV	EAS	390
HG02032	KHV	EAS	386
HG02035	KHV	EAS	340
HG02040	KHV	EAS	371
HG02047	KHV	EAS	370
HG02048	KHV	EAS	378
HG02049	KHV	EAS	382
HG02050	KHV	EAS	415
HG02051	ACB	AFR	474
HG02052	ACB	AFR	501
HG02053	ACB	AFR	437
HG02054	ACB	AFR	449
HG02057	KHV	EAS	382
HG02058	KHV	EAS	365
HG02060	KHV	EAS	403
HG02061	KHV	EAS	364
HG02064	KHV	EAS	391
HG02067	KHV	EAS	383
HG02069	KHV	EAS	379
HG02070	KHV	EAS	399
HG02072	KHV	EAS	395
HG02073	KHV	EAS	392
HG02075	KHV	EAS	361
HG02076	KHV	EAS	381
HG02078	KHV	EAS	362
HG02079	KHV	EAS	396
HG02081	KHV	EAS	362
HG02082	KHV	EAS	365
HG02084	KHV	EAS	375
HG02085	KHV	EAS	402
HG02086	KHV	EAS	343
HG02087	KHV	EAS	393
HG02088	KHV	EAS	366
HG02089	PEL	AMR	409
HG02090	PEL	AMR	370
HG02095	ACB	AFR	499
HG02102	PEL	AMR	392
HG02104	PEL	AMR	379
HG02105	PEL	AMR	375
HG02107	ACB	AFR	488

HG02108	ACB	AFR	454
HG02111	ACB	AFR	464
HG02113	KHV	EAS	404
HG02116	KHV	EAS	382
HG02121	KHV	EAS	376
HG02122	KHV	EAS	373
HG02127	KHV	EAS	380
HG02128	KHV	EAS	384
HG02130	KHV	EAS	373
HG02131	KHV	EAS	366
HG02133	KHV	EAS	398
HG02134	KHV	EAS	397
HG02136	KHV	EAS	369
HG02137	KHV	EAS	417
HG02138	KHV	EAS	385
HG02139	KHV	EAS	390
HG02140	KHV	EAS	369
HG02141	KHV	EAS	394
HG02142	KHV	EAS	360
HG02143	ACB	AFR	464
HG02144	ACB	AFR	476
HG02146	PEL	AMR	359
HG02147	PEL	AMR	361
HG02150	PEL	AMR	372
HG02151	CDX	EAS	365
HG02152	CDX	EAS	386
HG02153	CDX	EAS	369
HG02154	CDX	EAS	362
HG02155	CDX	EAS	355
HG02156	CDX	EAS	362
HG02164	CDX	EAS	359
HG02165	CDX	EAS	394
HG02166	CDX	EAS	396
HG02178	CDX	EAS	390
HG02179	CDX	EAS	364
HG02180	CDX	EAS	369
HG02181	CDX	EAS	392
HG02182	CDX	EAS	355
HG02184	CDX	EAS	379
HG02185	CDX	EAS	389
HG02186	CDX	EAS	380
HG02187	CDX	EAS	346
HG02188	CDX	EAS	371
HG02190	CDX	EAS	345
HG02215	GBR	EUR	354
HG02219	IBS	EUR	325
HG02220	IBS	EUR	360
HG02221	IBS	EUR	370
HG02223	IBS	EUR	347
HG02224	IBS	EUR	318

HG02230	IBS	EUR	314
HG02231	IBS	EUR	361
HG02232	IBS	EUR	371
HG02233	IBS	EUR	360
HG02235	IBS	EUR	368
HG02236	IBS	EUR	376
HG02238	IBS	EUR	416
HG02239	IBS	EUR	378
HG02250	CDX	EAS	361
HG02252	PEL	AMR	344
HG02253	PEL	AMR	385
HG02255	ACB	AFR	476
HG02256	ACB	AFR	435
HG02259	PEL	AMR	383
HG02260	PEL	AMR	345
HG02262	PEL	AMR	348
HG02265	PEL	AMR	390
HG02266	PEL	AMR	357
HG02271	PEL	AMR	398
HG02272	PEL	AMR	373
HG02274	PEL	AMR	392
HG02275	PEL	AMR	353
HG02277	PEL	AMR	348
HG02278	PEL	AMR	367
HG02281	ACB	AFR	472
HG02282	ACB	AFR	473
HG02283	ACB	AFR	441
HG02284	ACB	AFR	458
HG02285	PEL	AMR	382
HG02286	PEL	AMR	360
HG02291	PEL	AMR	386
HG02292	PEL	AMR	351
HG02298	PEL	AMR	356
HG02299	PEL	AMR	354
HG02301	PEL	AMR	373
HG02304	PEL	AMR	360
HG02307	ACB	AFR	461
HG02308	ACB	AFR	515
HG02309	ACB	AFR	476
HG02312	PEL	AMR	369
HG02314	ACB	AFR	476
HG02315	ACB	AFR	463
HG02317	ACB	AFR	500
HG02318	ACB	AFR	457
HG02322	ACB	AFR	428
HG02323	ACB	AFR	461
HG02325	ACB	AFR	484
HG02330	ACB	AFR	443
HG02332	ACB	AFR	512
HG02334	ACB	AFR	422

HG02337	ACB	AFR	468
HG02339	ACB	AFR	470
HG02343	ACB	AFR	463
HG02345	PEL	AMR	366
HG02348	PEL	AMR	383
HG02351	CDX	EAS	368
HG02353	CDX	EAS	362
HG02355	CDX	EAS	359
HG02356	CDX	EAS	376
HG02360	CDX	EAS	360
HG02364	CDX	EAS	371
HG02367	CDX	EAS	345
HG02371	CDX	EAS	385
HG02373	CDX	EAS	372
HG02374	CDX	EAS	403
HG02375	CDX	EAS	381
HG02379	CDX	EAS	375
HG02380	CDX	EAS	390
HG02382	CDX	EAS	394
HG02383	CDX	EAS	378
HG02384	CDX	EAS	347
HG02385	CDX	EAS	355
HG02386	CDX	EAS	400
HG02389	CDX	EAS	395
HG02390	CDX	EAS	367
HG02391	CDX	EAS	371
HG02392	CDX	EAS	371
HG02394	CDX	EAS	397
HG02395	CDX	EAS	373
HG02396	CDX	EAS	356
HG02397	CDX	EAS	360
HG02398	CDX	EAS	376
HG02399	CDX	EAS	399
HG02401	CDX	EAS	357
HG02402	CDX	EAS	385
HG02406	CDX	EAS	377
HG02407	CDX	EAS	358
HG02408	CDX	EAS	405
HG02409	CDX	EAS	349
HG02410	CDX	EAS	374
HG02419	ACB	AFR	473
HG02420	ACB	AFR	424
HG02425	PEL	AMR	356
HG02427	ACB	AFR	484
HG02429	ACB	AFR	463
HG02433	ACB	AFR	487
HG02439	ACB	AFR	494
HG02442	ACB	AFR	460
HG02445	ACB	AFR	476
HG02449	ACB	AFR	445

HG02450	ACB	AFR	439
HG02455	ACB	AFR	441
HG02461	GWD	AFR	504
HG02462	GWD	AFR	470
HG02464	GWD	AFR	489
HG02465	GWD	AFR	492
HG02470	ACB	AFR	477
HG02471	ACB	AFR	453
HG02476	ACB	AFR	476
HG02477	ACB	AFR	439
HG02479	ACB	AFR	459
HG02481	ACB	AFR	491
HG02484	ACB	AFR	437
HG02485	ACB	AFR	445
HG02489	ACB	AFR	453
HG02490	PJL	SAS	324
HG02491	PJL	SAS	375
HG02493	PJL	SAS	332
HG02494	PJL	SAS	344
HG02496	ACB	AFR	465
HG02497	ACB	AFR	453
HG02501	ACB	AFR	454
HG02502	ACB	AFR	474
HG02505	ACB	AFR	455
HG02508	ACB	AFR	454
HG02511	ACB	AFR	471
HG02512	KHV	EAS	366
HG02513	KHV	EAS	376
HG02521	KHV	EAS	364
HG02522	KHV	EAS	378
HG02536	ACB	AFR	451
HG02537	ACB	AFR	498
HG02541	ACB	AFR	455
HG02545	ACB	AFR	465
HG02546	ACB	AFR	506
HG02549	ACB	AFR	497
HG02554	ACB	AFR	437
HG02555	ACB	AFR	456
HG02557	ACB	AFR	466
HG02558	ACB	AFR	469
HG02561	GWD	AFR	469
HG02562	GWD	AFR	483
HG02568	GWD	AFR	486
HG02570	GWD	AFR	529
HG02571	GWD	AFR	488
HG02573	GWD	AFR	462
HG02574	GWD	AFR	483
HG02577	ACB	AFR	474
HG02580	ACB	AFR	428
HG02582	GWD	AFR	469

HG02583	GWD	AFR	487
HG02585	GWD	AFR	514
HG02586	GWD	AFR	478
HG02588	GWD	AFR	502
HG02589	GWD	AFR	496
HG02594	GWD	AFR	504
HG02595	GWD	AFR	436
HG02597	PJL	SAS	341
HG02600	PJL	SAS	361
HG02601	PJL	SAS	376
HG02603	PJL	SAS	364
HG02604	PJL	SAS	367
HG02610	GWD	AFR	522
HG02611	GWD	AFR	484
HG02613	GWD	AFR	485
HG02614	GWD	AFR	473
HG02620	GWD	AFR	518
HG02621	GWD	AFR	482
HG02623	GWD	AFR	452
HG02624	GWD	AFR	501
HG02628	GWD	AFR	471
HG02629	GWD	AFR	485
HG02634	GWD	AFR	461
HG02635	GWD	AFR	509
HG02642	GWD	AFR	475
HG02643	GWD	AFR	465
HG02645	GWD	AFR	509
HG02646	GWD	AFR	506
HG02648	PJL	SAS	369
HG02649	PJL	SAS	319
HG02651	PJL	SAS	364
HG02652	PJL	SAS	357
HG02654	PJL	SAS	378
HG02655	PJL	SAS	341
HG02657	PJL	SAS	371
HG02658	PJL	SAS	376
HG02660	PJL	SAS	366
HG02661	PJL	SAS	359
HG02666	GWD	AFR	475
HG02667	GWD	AFR	449
HG02675	GWD	AFR	496
HG02676	GWD	AFR	506
HG02678	GWD	AFR	455
HG02679	GWD	AFR	476
HG02681	PJL	SAS	356
HG02682	PJL	SAS	399
HG02684	PJL	SAS	342
HG02685	PJL	SAS	355
HG02687	PJL	SAS	359
HG02688	PJL	SAS	334

HG02690	PJL	SAS	345
HG02691	PJL	SAS	347
HG02694	PJL	SAS	358
HG02696	PJL	SAS	349
HG02697	PJL	SAS	365
HG02699	PJL	SAS	339
HG02700	PJL	SAS	333
HG02702	GWD	AFR	467
HG02703	GWD	AFR	473
HG02715	GWD	AFR	484
HG02716	GWD	AFR	470
HG02721	GWD	AFR	456
HG02722	GWD	AFR	471
HG02724	PJL	SAS	359
HG02725	PJL	SAS	366
HG02727	PJL	SAS	357
HG02728	PJL	SAS	349
HG02731	PJL	SAS	338
HG02733	PJL	SAS	367
HG02734	PJL	SAS	373
HG02736	PJL	SAS	372
HG02737	PJL	SAS	371
HG02756	GWD	AFR	486
HG02757	GWD	AFR	476
HG02759	GWD	AFR	473
HG02760	GWD	AFR	489
HG02763	GWD	AFR	434
HG02768	GWD	AFR	470
HG02769	GWD	AFR	482
HG02771	GWD	AFR	470
HG02772	GWD	AFR	461
HG02774	PJL	SAS	334
HG02775	PJL	SAS	375
HG02778	PJL	SAS	399
HG02780	PJL	SAS	361
HG02783	PJL	SAS	383
HG02784	PJL	SAS	380
HG02786	PJL	SAS	350
HG02787	PJL	SAS	349
HG02789	PJL	SAS	357
HG02790	PJL	SAS	376
HG02792	PJL	SAS	344
HG02793	PJL	SAS	389
HG02798	GWD	AFR	441
HG02799	GWD	AFR	509
HG02804	GWD	AFR	513
HG02805	GWD	AFR	462
HG02807	GWD	AFR	437
HG02808	GWD	AFR	482
HG02810	GWD	AFR	448

HG02811	GWD	AFR	473
HG02813	GWD	AFR	477
HG02814	GWD	AFR	491
HG02816	GWD	AFR	462
HG02817	GWD	AFR	440
HG02819	GWD	AFR	497
HG02820	GWD	AFR	471
HG02836	GWD	AFR	472
HG02837	GWD	AFR	460
HG02839	GWD	AFR	446
HG02840	GWD	AFR	478
HG02851	GWD	AFR	447
HG02852	GWD	AFR	474
HG02854	GWD	AFR	486
HG02855	GWD	AFR	524
HG02860	GWD	AFR	447
HG02861	GWD	AFR	514
HG02870	GWD	AFR	458
HG02878	GWD	AFR	487
HG02879	GWD	AFR	496
HG02881	GWD	AFR	456
HG02882	GWD	AFR	493
HG02884	GWD	AFR	485
HG02885	GWD	AFR	470
HG02887	GWD	AFR	488
HG02888	GWD	AFR	461
HG02890	GWD	AFR	475
HG02891	GWD	AFR	452
HG02895	GWD	AFR	472
HG02896	GWD	AFR	453
HG02922	ESN	AFR	492
HG02923	ESN	AFR	514
HG02938	ESN	AFR	488
HG02941	ESN	AFR	457
HG02943	ESN	AFR	474
HG02944	ESN	AFR	486
HG02946	ESN	AFR	454
HG02947	ESN	AFR	451
HG02952	ESN	AFR	478
HG02953	ESN	AFR	501
HG02968	ESN	AFR	475
HG02970	ESN	AFR	459
HG02971	ESN	AFR	464
HG02973	ESN	AFR	439
HG02974	ESN	AFR	484
HG02976	ESN	AFR	481
HG02977	ESN	AFR	498
HG02979	ESN	AFR	498
HG02981	ESN	AFR	434
HG02982	GWD	AFR	494

HG02983	GWD	AFR	463
HG03006	BEB	SAS	356
HG03007	BEB	SAS	344
HG03009	BEB	SAS	368
HG03012	BEB	SAS	357
HG03015	PJL	SAS	355
HG03016	PJL	SAS	351
HG03018	PJL	SAS	354
HG03019	PJL	SAS	386
HG03021	PJL	SAS	351
HG03022	PJL	SAS	346
HG03024	GWD	AFR	502
HG03025	GWD	AFR	452
HG03027	GWD	AFR	518
HG03028	GWD	AFR	466
HG03039	GWD	AFR	511
HG03040	GWD	AFR	500
HG03045	GWD	AFR	483
HG03046	GWD	AFR	498
HG03048	GWD	AFR	449
HG03049	GWD	AFR	467
HG03052	MSL	AFR	449
HG03054	MSL	AFR	491
HG03055	MSL	AFR	522
HG03057	MSL	AFR	499
HG03058	MSL	AFR	486
HG03060	MSL	AFR	473
HG03061	MSL	AFR	454
HG03063	MSL	AFR	470
HG03064	MSL	AFR	531
HG03066	MSL	AFR	518
HG03069	MSL	AFR	511
HG03072	MSL	AFR	513
HG03073	MSL	AFR	434
HG03074	MSL	AFR	484
HG03077	MSL	AFR	487
HG03078	MSL	AFR	479
HG03079	MSL	AFR	483
HG03081	MSL	AFR	483
HG03082	MSL	AFR	463
HG03084	MSL	AFR	498
HG03085	MSL	AFR	473
HG03086	MSL	AFR	488
HG03088	MSL	AFR	462
HG03091	MSL	AFR	477
HG03095	MSL	AFR	465
HG03096	MSL	AFR	491
HG03097	MSL	AFR	476
HG03099	ESN	AFR	473
HG03100	ESN	AFR	471

HG03103	ESN	AFR	493
HG03105	ESN	AFR	480
HG03108	ESN	AFR	461
HG03109	ESN	AFR	488
HG03111	ESN	AFR	463
HG03112	ESN	AFR	496
HG03114	ESN	AFR	483
HG03115	ESN	AFR	454
HG03117	ESN	AFR	484
HG03118	ESN	AFR	467
HG03120	ESN	AFR	510
HG03121	ESN	AFR	446
HG03123	ESN	AFR	497
HG03124	ESN	AFR	493
HG03126	ESN	AFR	481
HG03127	ESN	AFR	481
HG03129	ESN	AFR	465
HG03130	ESN	AFR	451
HG03132	ESN	AFR	460
HG03133	ESN	AFR	481
HG03135	ESN	AFR	434
HG03136	ESN	AFR	470
HG03139	ESN	AFR	452
HG03157	ESN	AFR	506
HG03159	ESN	AFR	461
HG03160	ESN	AFR	513
HG03162	ESN	AFR	483
HG03163	ESN	AFR	462
HG03166	ESN	AFR	491
HG03168	ESN	AFR	441
HG03169	ESN	AFR	473
HG03172	ESN	AFR	463
HG03175	ESN	AFR	508
HG03189	ESN	AFR	423
HG03190	ESN	AFR	450
HG03193	ESN	AFR	482
HG03195	ESN	AFR	497
HG03196	ESN	AFR	485
HG03198	ESN	AFR	494
HG03199	ESN	AFR	519
HG03202	ESN	AFR	489
HG03209	MSL	AFR	484
HG03212	MSL	AFR	497
HG03224	MSL	AFR	454
HG03225	MSL	AFR	501
HG03228	PJL	SAS	375
HG03229	PJL	SAS	357
HG03234	PJL	SAS	372
HG03235	PJL	SAS	335
HG03237	PJL	SAS	354

HG03238	PJL	SAS	374
HG03240	GWD	AFR	482
HG03241	GWD	AFR	509
HG03246	GWD	AFR	508
HG03247	GWD	AFR	444
HG03258	GWD	AFR	472
HG03259	GWD	AFR	461
HG03265	ESN	AFR	480
HG03267	ESN	AFR	484
HG03268	ESN	AFR	483
HG03270	ESN	AFR	497
HG03271	ESN	AFR	486
HG03279	ESN	AFR	484
HG03280	ESN	AFR	470
HG03291	ESN	AFR	498
HG03294	ESN	AFR	483
HG03295	ESN	AFR	468
HG03297	ESN	AFR	472
HG03298	ESN	AFR	452
HG03300	ESN	AFR	502
HG03301	ESN	AFR	445
HG03303	ESN	AFR	472
HG03304	ESN	AFR	485
HG03311	ESN	AFR	472
HG03313	ESN	AFR	516
HG03342	ESN	AFR	496
HG03343	ESN	AFR	481
HG03351	ESN	AFR	482
HG03352	ESN	AFR	465
HG03354	ESN	AFR	450
HG03363	ESN	AFR	513
HG03366	ESN	AFR	452
HG03367	ESN	AFR	490
HG03369	ESN	AFR	483
HG03370	ESN	AFR	472
HG03372	ESN	AFR	459
HG03376	MSL	AFR	455
HG03378	MSL	AFR	458
HG03380	MSL	AFR	484
HG03382	MSL	AFR	451
HG03385	MSL	AFR	484
HG03388	MSL	AFR	455
HG03391	MSL	AFR	465
HG03394	MSL	AFR	446
HG03397	MSL	AFR	466
HG03401	MSL	AFR	499
HG03410	MSL	AFR	454
HG03419	MSL	AFR	454
HG03428	MSL	AFR	490
HG03432	MSL	AFR	472

HG03433	MSL	AFR	509
HG03436	MSL	AFR	461
HG03437	MSL	AFR	478
HG03439	MSL	AFR	452
HG03442	MSL	AFR	499
HG03445	MSL	AFR	489
HG03446	MSL	AFR	484
HG03449	MSL	AFR	450
HG03451	MSL	AFR	497
HG03452	MSL	AFR	501
HG03455	MSL	AFR	473
HG03457	MSL	AFR	518
HG03458	MSL	AFR	457
HG03460	MSL	AFR	460
HG03461	MSL	AFR	502
HG03464	MSL	AFR	511
HG03469	MSL	AFR	490
HG03470	MSL	AFR	489
HG03472	MSL	AFR	462
HG03473	MSL	AFR	507
HG03476	MSL	AFR	441
HG03478	MSL	AFR	489
HG03479	MSL	AFR	476
HG03484	MSL	AFR	441
HG03485	MSL	AFR	470
HG03488	PJL	SAS	345
HG03490	PJL	SAS	355
HG03491	PJL	SAS	360
HG03499	ESN	AFR	513
HG03511	ESN	AFR	491
HG03514	ESN	AFR	491
HG03515	ESN	AFR	485
HG03517	ESN	AFR	455
HG03518	ESN	AFR	477
HG03520	ESN	AFR	496
HG03521	ESN	AFR	438
HG03538	GWD	AFR	494
HG03539	GWD	AFR	459
HG03547	MSL	AFR	458
HG03548	MSL	AFR	476
HG03556	MSL	AFR	490
HG03557	MSL	AFR	474
HG03558	MSL	AFR	502
HG03559	MSL	AFR	493
HG03563	MSL	AFR	457
HG03565	MSL	AFR	487
HG03567	MSL	AFR	486
HG03571	MSL	AFR	485
HG03572	MSL	AFR	484
HG03575	MSL	AFR	490

HG03577	MSL	AFR	511
HG03578	MSL	AFR	456
HG03583	MSL	AFR	462
HG03585	BEB	SAS	341
HG03589	BEB	SAS	345
HG03593	BEB	SAS	380
HG03594	BEB	SAS	370
HG03595	BEB	SAS	356
HG03598	BEB	SAS	320
HG03600	BEB	SAS	350
HG03603	BEB	SAS	358
HG03604	BEB	SAS	371
HG03607	BEB	SAS	342
HG03611	BEB	SAS	358
HG03615	BEB	SAS	360
HG03616	BEB	SAS	362
HG03619	PJL	SAS	333
HG03624	PJL	SAS	348
HG03625	PJL	SAS	340
HG03629	PJL	SAS	374
HG03631	PJL	SAS	399
HG03634	PJL	SAS	335
HG03636	PJL	SAS	363
HG03640	PJL	SAS	363
HG03642	STU	SAS	369
HG03643	STU	SAS	328
HG03644	STU	SAS	317
HG03645	STU	SAS	334
HG03646	STU	SAS	352
HG03649	PJL	SAS	363
HG03652	PJL	SAS	385
HG03653	PJL	SAS	362
HG03660	PJL	SAS	332
HG03663	PJL	SAS	385
HG03667	PJL	SAS	348
HG03668	PJL	SAS	350
HG03672	STU	SAS	338
HG03673	STU	SAS	341
HG03679	STU	SAS	360
HG03680	STU	SAS	358
HG03681	STU	SAS	362
HG03684	STU	SAS	369
HG03685	STU	SAS	370
HG03686	STU	SAS	382
HG03687	STU	SAS	319
HG03689	STU	SAS	354
HG03690	STU	SAS	318
HG03691	STU	SAS	333
HG03692	STU	SAS	348
HG03693	STU	SAS	366

HG03694	STU	SAS	351
HG03695	STU	SAS	368
HG03696	STU	SAS	363
HG03697	STU	SAS	331
HG03698	STU	SAS	324
HG03702	PJL	SAS	340
HG03703	PJL	SAS	357
HG03705	PJL	SAS	336
HG03706	PJL	SAS	349
HG03708	PJL	SAS	359
HG03709	PJL	SAS	379
HG03711	STU	SAS	400
HG03713	ITU	SAS	366
HG03714	ITU	SAS	381
HG03716	ITU	SAS	372
HG03717	ITU	SAS	362
HG03718	ITU	SAS	308
HG03720	ITU	SAS	339
HG03722	ITU	SAS	335
HG03727	ITU	SAS	369
HG03729	ITU	SAS	377
HG03730	ITU	SAS	343
HG03731	ITU	SAS	377
HG03733	STU	SAS	337
HG03736	STU	SAS	353
HG03738	STU	SAS	325
HG03740	STU	SAS	339
HG03741	STU	SAS	340
HG03742	ITU	SAS	354
HG03743	STU	SAS	367
HG03744	STU	SAS	344
HG03745	STU	SAS	367
HG03746	STU	SAS	340
HG03750	STU	SAS	391
HG03752	STU	SAS	351
HG03753	STU	SAS	351
HG03754	STU	SAS	394
HG03755	STU	SAS	346
HG03756	STU	SAS	358
HG03757	STU	SAS	369
HG03760	STU	SAS	330
HG03762	PJL	SAS	338
HG03765	PJL	SAS	351
HG03767	PJL	SAS	366
HG03770	ITU	SAS	374
HG03771	ITU	SAS	365
HG03772	ITU	SAS	356
HG03773	ITU	SAS	380
HG03774	ITU	SAS	361
HG03775	ITU	SAS	358

HG03777	ITU	SAS	337
HG03778	ITU	SAS	347
HG03779	ITU	SAS	367
HG03780	ITU	SAS	336
HG03781	ITU	SAS	360
HG03782	ITU	SAS	386
HG03784	ITU	SAS	356
HG03785	ITU	SAS	320
HG03786	ITU	SAS	362
HG03787	ITU	SAS	366
HG03788	ITU	SAS	368
HG03789	ITU	SAS	346
HG03790	ITU	SAS	362
HG03792	ITU	SAS	368
HG03793	BEB	SAS	361
HG03796	BEB	SAS	370
HG03800	BEB	SAS	390
HG03802	BEB	SAS	340
HG03803	BEB	SAS	385
HG03805	BEB	SAS	323
HG03808	BEB	SAS	318
HG03809	BEB	SAS	351
HG03812	BEB	SAS	342
HG03814	BEB	SAS	371
HG03815	BEB	SAS	368
HG03817	BEB	SAS	344
HG03821	BEB	SAS	369
HG03823	BEB	SAS	357
HG03824	BEB	SAS	385
HG03826	BEB	SAS	392
HG03829	BEB	SAS	362
HG03830	BEB	SAS	352
HG03832	BEB	SAS	369
HG03833	BEB	SAS	374
HG03836	STU	SAS	366
HG03837	STU	SAS	358
HG03838	STU	SAS	364
HG03844	STU	SAS	333
HG03846	STU	SAS	344
HG03848	STU	SAS	327
HG03849	STU	SAS	351
HG03850	STU	SAS	369
HG03851	STU	SAS	320
HG03854	STU	SAS	365
HG03856	STU	SAS	339
HG03857	STU	SAS	391
HG03858	STU	SAS	341
HG03861	ITU	SAS	392
HG03862	ITU	SAS	343
HG03863	ITU	SAS	395

HG03864	ITU	SAS	345
HG03866	ITU	SAS	372
HG03867	ITU	SAS	365
HG03868	ITU	SAS	365
HG03869	ITU	SAS	347
HG03870	ITU	SAS	350
HG03871	ITU	SAS	358
HG03872	ITU	SAS	393
HG03873	ITU	SAS	364
HG03874	ITU	SAS	345
HG03875	ITU	SAS	343
HG03882	ITU	SAS	386
HG03884	STU	SAS	332
HG03885	STU	SAS	341
HG03886	STU	SAS	355
HG03887	STU	SAS	348
HG03888	STU	SAS	379
HG03890	STU	SAS	342
HG03894	STU	SAS	363
HG03895	STU	SAS	334
HG03896	STU	SAS	378
HG03897	STU	SAS	375
HG03898	STU	SAS	344
HG03899	STU	SAS	308
HG03900	STU	SAS	392
HG03902	BEB	SAS	345
HG03905	BEB	SAS	370
HG03907	BEB	SAS	383
HG03908	BEB	SAS	388
HG03910	BEB	SAS	337
HG03911	BEB	SAS	348
HG03913	BEB	SAS	369
HG03914	BEB	SAS	330
HG03916	BEB	SAS	346
HG03917	BEB	SAS	311
HG03919	BEB	SAS	362
HG03920	BEB	SAS	353
HG03922	BEB	SAS	354
HG03925	BEB	SAS	359
HG03926	BEB	SAS	339
HG03928	BEB	SAS	339
HG03931	BEB	SAS	358
HG03934	BEB	SAS	362
HG03937	BEB	SAS	352
HG03940	BEB	SAS	352
HG03941	BEB	SAS	328
HG03943	STU	SAS	376
HG03944	STU	SAS	361
HG03945	STU	SAS	383
HG03947	STU	SAS	353

HG03949	STU	SAS	406
HG03950	STU	SAS	360
HG03951	STU	SAS	356
HG03953	STU	SAS	385
HG03955	STU	SAS	365
HG03960	ITU	SAS	358
HG03963	ITU	SAS	352
HG03965	ITU	SAS	362
HG03967	ITU	SAS	360
HG03968	ITU	SAS	359
HG03969	ITU	SAS	371
HG03971	ITU	SAS	332
HG03973	ITU	SAS	352
HG03974	ITU	SAS	356
HG03976	ITU	SAS	342
HG03977	ITU	SAS	328
HG03978	ITU	SAS	366
HG03985	STU	SAS	367
HG03986	STU	SAS	389
HG03989	STU	SAS	343
HG03990	STU	SAS	353
HG03991	STU	SAS	389
HG03995	STU	SAS	348
HG03998	STU	SAS	352
HG03999	STU	SAS	368
HG04001	ITU	SAS	329
HG04002	ITU	SAS	339
HG04003	STU	SAS	340
HG04006	STU	SAS	378
HG04014	ITU	SAS	337
HG04015	ITU	SAS	381
HG04017	ITU	SAS	350
HG04018	ITU	SAS	347
HG04019	ITU	SAS	372
HG04020	ITU	SAS	360
HG04022	ITU	SAS	347
HG04023	ITU	SAS	354
HG04025	ITU	SAS	360
HG04026	ITU	SAS	391
HG04029	STU	SAS	365
HG04033	STU	SAS	361
HG04035	STU	SAS	360
HG04038	STU	SAS	384
HG04039	STU	SAS	348
HG04042	STU	SAS	354
HG04047	STU	SAS	351
HG04054	ITU	SAS	367
HG04056	ITU	SAS	368
HG04059	ITU	SAS	352
HG04060	ITU	SAS	373

HG04061	ITU	SAS	354
HG04062	ITU	SAS	353
HG04063	ITU	SAS	355
HG04070	ITU	SAS	369
HG04075	STU	SAS	348
HG04076	ITU	SAS	373
HG04080	ITU	SAS	343
HG04090	ITU	SAS	363
HG04093	ITU	SAS	369
HG04094	ITU	SAS	348
HG04096	ITU	SAS	387
HG04098	ITU	SAS	360
HG04099	STU	SAS	372
HG04100	STU	SAS	368
HG04106	STU	SAS	317
HG04107	STU	SAS	369
HG04118	ITU	SAS	389
HG04131	BEB	SAS	361
HG04134	BEB	SAS	361
HG04140	BEB	SAS	380
HG04141	BEB	SAS	375
HG04144	BEB	SAS	377
HG04146	BEB	SAS	371
HG04152	BEB	SAS	379
HG04153	BEB	SAS	339
HG04155	BEB	SAS	354
HG04156	BEB	SAS	370
HG04158	BEB	SAS	337
HG04159	BEB	SAS	353
HG04161	BEB	SAS	360
HG04162	BEB	SAS	329
HG04164	BEB	SAS	379
HG04171	BEB	SAS	335
HG04173	BEB	SAS	338
HG04176	BEB	SAS	373
HG04177	BEB	SAS	344
HG04180	BEB	SAS	341
HG04182	BEB	SAS	324
HG04183	BEB	SAS	389
HG04185	BEB	SAS	364
HG04186	BEB	SAS	358
HG04188	BEB	SAS	386
HG04189	BEB	SAS	324
HG04194	BEB	SAS	375
HG04195	BEB	SAS	343
HG04198	ITU	SAS	376
HG04200	ITU	SAS	335
HG04202	ITU	SAS	342
HG04206	ITU	SAS	355
HG04209	ITU	SAS	344

HG04210	STU	SAS	349
HG04211	ITU	SAS	362
HG04212	ITU	SAS	368
HG04214	ITU	SAS	399
HG04216	ITU	SAS	360
HG04219	ITU	SAS	347
HG04222	ITU	SAS	340
HG04225	ITU	SAS	354
HG04227	STU	SAS	356
HG04229	STU	SAS	341
HG04235	ITU	SAS	339
HG04238	ITU	SAS	352
HG04239	ITU	SAS	372
NA06984	CEU	EUR	343
NA06985	CEU	EUR	351
NA06986	CEU	EUR	349
NA06989	CEU	EUR	342
NA06994	CEU	EUR	300
NA07000	CEU	EUR	377
NA07037	CEU	EUR	352
NA07048	CEU	EUR	360
NA07051	CEU	EUR	345
NA07056	CEU	EUR	390
NA07347	CEU	EUR	402
NA07357	CEU	EUR	347
NA10847	CEU	EUR	359
NA10851	CEU	EUR	355
NA11829	CEU	EUR	333
NA11830	CEU	EUR	343
NA11831	CEU	EUR	370
NA11832	CEU	EUR	334
NA11840	CEU	EUR	344
NA11843	CEU	EUR	373
NA11881	CEU	EUR	333
NA11892	CEU	EUR	374
NA11893	CEU	EUR	394
NA11894	CEU	EUR	335
NA11918	CEU	EUR	340
NA11919	CEU	EUR	353
NA11920	CEU	EUR	316
NA11930	CEU	EUR	345
NA11931	CEU	EUR	379
NA11932	CEU	EUR	348
NA11933	CEU	EUR	348
NA11992	CEU	EUR	348
NA11994	CEU	EUR	370
NA11995	CEU	EUR	352
NA12003	CEU	EUR	337
NA12004	CEU	EUR	349
NA12005	CEU	EUR	352

NA12006	CEU	EUR	368
NA12043	CEU	EUR	334
NA12044	CEU	EUR	335
NA12045	CEU	EUR	389
NA12046	CEU	EUR	356
NA12058	CEU	EUR	352
NA12144	CEU	EUR	343
NA12154	CEU	EUR	368
NA12155	CEU	EUR	369
NA12156	CEU	EUR	348
NA12234	CEU	EUR	369
NA12249	CEU	EUR	356
NA12272	CEU	EUR	336
NA12273	CEU	EUR	336
NA12275	CEU	EUR	354
NA12282	CEU	EUR	344
NA12283	CEU	EUR	363
NA12286	CEU	EUR	341
NA12287	CEU	EUR	337
NA12340	CEU	EUR	352
NA12341	CEU	EUR	336
NA12342	CEU	EUR	377
NA12347	CEU	EUR	337
NA12348	CEU	EUR	355
NA12383	CEU	EUR	357
NA12399	CEU	EUR	332
NA12400	CEU	EUR	285
NA12413	CEU	EUR	381
NA12414	CEU	EUR	364
NA12489	CEU	EUR	373
NA12546	CEU	EUR	366
NA12716	CEU	EUR	369
NA12717	CEU	EUR	345
NA12718	CEU	EUR	373
NA12748	CEU	EUR	371
NA12749	CEU	EUR	368
NA12750	CEU	EUR	354
NA12751	CEU	EUR	354
NA12760	CEU	EUR	358
NA12761	CEU	EUR	359
NA12762	CEU	EUR	371
NA12763	CEU	EUR	355
NA12775	CEU	EUR	365
NA12776	CEU	EUR	378
NA12777	CEU	EUR	381
NA12778	CEU	EUR	345
NA12812	CEU	EUR	370
NA12813	CEU	EUR	354
NA12814	CEU	EUR	371
NA12815	CEU	EUR	360

NA12827	CEU	EUR	341
NA12828	CEU	EUR	341
NA12829	CEU	EUR	352
NA12830	CEU	EUR	354
NA12842	CEU	EUR	358
NA12843	CEU	EUR	348
NA12872	CEU	EUR	335
NA12873	CEU	EUR	368
NA12874	CEU	EUR	365
NA12878	CEU	EUR	337
NA12889	CEU	EUR	347
NA12890	CEU	EUR	347
NA18486	YRI	AFR	439
NA18488	YRI	AFR	494
NA18489	YRI	AFR	465
NA18498	YRI	AFR	472
NA18499	YRI	AFR	484
NA18501	YRI	AFR	484
NA18502	YRI	AFR	473
NA18504	YRI	AFR	492
NA18505	YRI	AFR	483
NA18507	YRI	AFR	481
NA18508	YRI	AFR	498
NA18510	YRI	AFR	450
NA18511	YRI	AFR	466
NA18516	YRI	AFR	442
NA18517	YRI	AFR	463
NA18519	YRI	AFR	482
NA18520	YRI	AFR	487
NA18522	YRI	AFR	448
NA18523	YRI	AFR	459
NA18525	CHB	EAS	409
NA18526	CHB	EAS	378
NA18528	CHB	EAS	404
NA18530	CHB	EAS	334
NA18531	CHB	EAS	377
NA18532	CHB	EAS	340
NA18533	CHB	EAS	385
NA18534	CHB	EAS	376
NA18535	CHB	EAS	392
NA18536	CHB	EAS	378
NA18537	CHB	EAS	362
NA18538	CHB	EAS	359
NA18539	CHB	EAS	373
NA18541	CHB	EAS	359
NA18542	CHB	EAS	372
NA18543	CHB	EAS	396
NA18544	CHB	EAS	455
NA18545	CHB	EAS	356
NA18546	CHB	EAS	352

NA18547	CHB	EAS	393
NA18548	CHB	EAS	361
NA18549	CHB	EAS	383
NA18550	CHB	EAS	360
NA18552	CHB	EAS	363
NA18553	CHB	EAS	393
NA18555	CHB	EAS	390
NA18557	CHB	EAS	386
NA18558	CHB	EAS	354
NA18559	CHB	EAS	379
NA18560	CHB	EAS	361
NA18561	CHB	EAS	341
NA18562	CHB	EAS	386
NA18563	CHB	EAS	380
NA18564	CHB	EAS	378
NA18565	CHB	EAS	408
NA18566	CHB	EAS	381
NA18567	CHB	EAS	381
NA18570	CHB	EAS	389
NA18571	CHB	EAS	396
NA18572	CHB	EAS	408
NA18573	CHB	EAS	377
NA18574	CHB	EAS	401
NA18577	CHB	EAS	382
NA18579	CHB	EAS	374
NA18582	CHB	EAS	392
NA18591	CHB	EAS	345
NA18592	CHB	EAS	371
NA18593	CHB	EAS	401
NA18595	CHB	EAS	401
NA18596	CHB	EAS	379
NA18597	CHB	EAS	402
NA18599	CHB	EAS	372
NA18602	CHB	EAS	376
NA18603	CHB	EAS	388
NA18605	CHB	EAS	383
NA18606	CHB	EAS	366
NA18608	CHB	EAS	396
NA18609	CHB	EAS	373
NA18610	CHB	EAS	373
NA18611	CHB	EAS	385
NA18612	CHB	EAS	414
NA18613	CHB	EAS	375
NA18614	CHB	EAS	380
NA18615	CHB	EAS	381
NA18616	CHB	EAS	368
NA18617	CHB	EAS	391
NA18618	CHB	EAS	357
NA18619	CHB	EAS	370
NA18620	CHB	EAS	350

NA18621	CHB	EAS	380
NA18622	CHB	EAS	394
NA18623	CHB	EAS	354
NA18624	CHB	EAS	408
NA18625	CHB	EAS	374
NA18626	CHB	EAS	346
NA18627	CHB	EAS	356
NA18628	CHB	EAS	366
NA18629	CHB	EAS	380
NA18630	CHB	EAS	336
NA18631	CHB	EAS	375
NA18632	CHB	EAS	364
NA18633	CHB	EAS	379
NA18634	CHB	EAS	399
NA18635	CHB	EAS	410
NA18636	CHB	EAS	378
NA18637	CHB	EAS	388
NA18638	CHB	EAS	374
NA18639	CHB	EAS	369
NA18640	CHB	EAS	397
NA18641	CHB	EAS	394
NA18642	CHB	EAS	365
NA18643	CHB	EAS	409
NA18644	CHB	EAS	356
NA18645	CHB	EAS	330
NA18646	CHB	EAS	384
NA18647	CHB	EAS	344
NA18648	CHB	EAS	365
NA18740	CHB	EAS	375
NA18745	CHB	EAS	371
NA18747	CHB	EAS	368
NA18748	CHB	EAS	373
NA18749	CHB	EAS	380
NA18757	CHB	EAS	369
NA18853	YRI	AFR	420
NA18856	YRI	AFR	451
NA18858	YRI	AFR	473
NA18861	YRI	AFR	519
NA18864	YRI	AFR	454
NA18865	YRI	AFR	458
NA18867	YRI	AFR	472
NA18868	YRI	AFR	515
NA18870	YRI	AFR	472
NA18871	YRI	AFR	458
NA18873	YRI	AFR	473
NA18874	YRI	AFR	456
NA18876	YRI	AFR	492
NA18877	YRI	AFR	459
NA18878	YRI	AFR	453
NA18879	YRI	AFR	479

NA18881	YRI	AFR	478
NA18907	YRI	AFR	485
NA18908	YRI	AFR	474
NA18909	YRI	AFR	500
NA18910	YRI	AFR	484
NA18912	YRI	AFR	497
NA18915	YRI	AFR	446
NA18916	YRI	AFR	490
NA18917	YRI	AFR	497
NA18923	YRI	AFR	536
NA18924	YRI	AFR	450
NA18933	YRI	AFR	467
NA18934	YRI	AFR	507
NA18939	JPT	EAS	375
NA18940	JPT	EAS	377
NA18941	JPT	EAS	371
NA18942	JPT	EAS	343
NA18943	JPT	EAS	363
NA18944	JPT	EAS	369
NA18945	JPT	EAS	403
NA18946	JPT	EAS	383
NA18947	JPT	EAS	330
NA18948	JPT	EAS	370
NA18949	JPT	EAS	381
NA18950	JPT	EAS	395
NA18951	JPT	EAS	368
NA18952	JPT	EAS	356
NA18953	JPT	EAS	374
NA18954	JPT	EAS	348
NA18956	JPT	EAS	352
NA18957	JPT	EAS	371
NA18959	JPT	EAS	370
NA18960	JPT	EAS	317
NA18961	JPT	EAS	359
NA18962	JPT	EAS	371
NA18963	JPT	EAS	363
NA18964	JPT	EAS	371
NA18965	JPT	EAS	392
NA18966	JPT	EAS	361
NA18967	JPT	EAS	379
NA18968	JPT	EAS	363
NA18969	JPT	EAS	343
NA18970	JPT	EAS	360
NA18971	JPT	EAS	371
NA18972	JPT	EAS	388
NA18973	JPT	EAS	367
NA18974	JPT	EAS	366
NA18975	JPT	EAS	377
NA18976	JPT	EAS	360
NA18977	JPT	EAS	369

NA18978	JPT	EAS	348
NA18979	JPT	EAS	369
NA18980	JPT	EAS	372
NA18981	JPT	EAS	362
NA18982	JPT	EAS	373
NA18983	JPT	EAS	392
NA18984	JPT	EAS	387
NA18985	JPT	EAS	369
NA18986	JPT	EAS	355
NA18987	JPT	EAS	388
NA18988	JPT	EAS	415
NA18989	JPT	EAS	421
NA18990	JPT	EAS	345
NA18991	JPT	EAS	380
NA18992	JPT	EAS	370
NA18993	JPT	EAS	400
NA18994	JPT	EAS	351
NA18995	JPT	EAS	372
NA18997	JPT	EAS	380
NA18998	JPT	EAS	392
NA18999	JPT	EAS	355
NA19000	JPT	EAS	414
NA19001	JPT	EAS	356
NA19002	JPT	EAS	375
NA19003	JPT	EAS	405
NA19004	JPT	EAS	377
NA19005	JPT	EAS	375
NA19006	JPT	EAS	382
NA19007	JPT	EAS	331
NA19009	JPT	EAS	365
NA19010	JPT	EAS	377
NA19011	JPT	EAS	363
NA19012	JPT	EAS	373
NA19017	LWK	AFR	448
NA19019	LWK	AFR	474
NA19020	LWK	AFR	477
NA19023	LWK	AFR	503
NA19024	LWK	AFR	437
NA19025	LWK	AFR	465
NA19026	LWK	AFR	453
NA19027	LWK	AFR	484
NA19028	LWK	AFR	477
NA19030	LWK	AFR	473
NA19031	LWK	AFR	454
NA19035	LWK	AFR	490
NA19036	LWK	AFR	509
NA19037	LWK	AFR	493
NA19038	LWK	AFR	500
NA19041	LWK	AFR	509
NA19042	LWK	AFR	459

NA19043	LWK	AFR	485
NA19054	JPT	EAS	353
NA19055	JPT	EAS	331
NA19056	JPT	EAS	355
NA19057	JPT	EAS	388
NA19058	JPT	EAS	376
NA19059	JPT	EAS	357
NA19060	JPT	EAS	338
NA19062	JPT	EAS	361
NA19063	JPT	EAS	351
NA19064	JPT	EAS	385
NA19065	JPT	EAS	382
NA19066	JPT	EAS	357
NA19067	JPT	EAS	380
NA19068	JPT	EAS	391
NA19070	JPT	EAS	359
NA19072	JPT	EAS	365
NA19074	JPT	EAS	356
NA19075	JPT	EAS	356
NA19076	JPT	EAS	364
NA19077	JPT	EAS	344
NA19078	JPT	EAS	376
NA19079	JPT	EAS	369
NA19080	JPT	EAS	357
NA19081	JPT	EAS	349
NA19082	JPT	EAS	372
NA19083	JPT	EAS	382
NA19084	JPT	EAS	367
NA19085	JPT	EAS	379
NA19086	JPT	EAS	400
NA19087	JPT	EAS	381
NA19088	JPT	EAS	367
NA19089	JPT	EAS	378
NA19090	JPT	EAS	377
NA19091	JPT	EAS	366
NA19092	YRI	AFR	459
NA19093	YRI	AFR	457
NA19095	YRI	AFR	504
NA19096	YRI	AFR	464
NA19098	YRI	AFR	487
NA19099	YRI	AFR	484
NA19102	YRI	AFR	488
NA19107	YRI	AFR	498
NA19108	YRI	AFR	475
NA19113	YRI	AFR	499
NA19114	YRI	AFR	450
NA19116	YRI	AFR	461
NA19117	YRI	AFR	509
NA19118	YRI	AFR	457
NA19119	YRI	AFR	462

NA19121	YRI	AFR	454
NA19129	YRI	AFR	443
NA19130	YRI	AFR	487
NA19131	YRI	AFR	477
NA19137	YRI	AFR	494
NA19138	YRI	AFR	500
NA19141	YRI	AFR	501
NA19143	YRI	AFR	485
NA19144	YRI	AFR	473
NA19146	YRI	AFR	446
NA19147	YRI	AFR	480
NA19149	YRI	AFR	467
NA19152	YRI	AFR	496
NA19153	YRI	AFR	492
NA19159	YRI	AFR	493
NA19160	YRI	AFR	495
NA19171	YRI	AFR	455
NA19172	YRI	AFR	474
NA19175	YRI	AFR	490
NA19184	YRI	AFR	459
NA19185	YRI	AFR	473
NA19189	YRI	AFR	442
NA19190	YRI	AFR	473
NA19197	YRI	AFR	441
NA19198	YRI	AFR	466
NA19200	YRI	AFR	488
NA19201	YRI	AFR	470
NA19204	YRI	AFR	526
NA19206	YRI	AFR	495
NA19207	YRI	AFR	459
NA19209	YRI	AFR	468
NA19210	YRI	AFR	451
NA19213	YRI	AFR	450
NA19214	YRI	AFR	515
NA19222	YRI	AFR	471
NA19223	YRI	AFR	440
NA19225	YRI	AFR	481
NA19235	YRI	AFR	458
NA19236	YRI	AFR	471
NA19238	YRI	AFR	440
NA19239	YRI	AFR	445
NA19247	YRI	AFR	461
NA19248	YRI	AFR	485
NA19256	YRI	AFR	460
NA19257	YRI	AFR	496
NA19307	LWK	AFR	462
NA19308	LWK	AFR	497
NA19309	LWK	AFR	452
NA19310	LWK	AFR	482
NA19312	LWK	AFR	503

NA19314	LWK	AFR	436
NA19315	LWK	AFR	471
NA19316	LWK	AFR	494
NA19317	LWK	AFR	471
NA19318	LWK	AFR	478
NA19319	LWK	AFR	467
NA19320	LWK	AFR	520
NA19321	LWK	AFR	453
NA19323	LWK	AFR	484
NA19324	LWK	AFR	458
NA19327	LWK	AFR	498
NA19328	LWK	AFR	444
NA19331	LWK	AFR	464
NA19332	LWK	AFR	469
NA19334	LWK	AFR	443
NA19338	LWK	AFR	479
NA19346	LWK	AFR	434
NA19347	LWK	AFR	489
NA19350	LWK	AFR	494
NA19351	LWK	AFR	448
NA19355	LWK	AFR	486
NA19360	LWK	AFR	515
NA19372	LWK	AFR	461
NA19374	LWK	AFR	472
NA19375	LWK	AFR	469
NA19376	LWK	AFR	447
NA19377	LWK	AFR	472
NA19378	LWK	AFR	488
NA19379	LWK	AFR	465
NA19380	LWK	AFR	461
NA19383	LWK	AFR	488
NA19384	LWK	AFR	434
NA19385	LWK	AFR	427
NA19390	LWK	AFR	477
NA19391	LWK	AFR	426
NA19393	LWK	AFR	485
NA19394	LWK	AFR	486
NA19395	LWK	AFR	483
NA19397	LWK	AFR	507
NA19399	LWK	AFR	452
NA19401	LWK	AFR	407
NA19403	LWK	AFR	489
NA19404	LWK	AFR	470
NA19428	LWK	AFR	470
NA19429	LWK	AFR	460
NA19430	LWK	AFR	456
NA19431	LWK	AFR	483
NA19434	LWK	AFR	455
NA19435	LWK	AFR	462
NA19436	LWK	AFR	447

NA19437	LWK	AFR	461
NA19438	LWK	AFR	451
NA19439	LWK	AFR	488
NA19440	LWK	AFR	493
NA19443	LWK	AFR	467
NA19445	LWK	AFR	447
NA19446	LWK	AFR	495
NA19448	LWK	AFR	467
NA19449	LWK	AFR	465
NA19451	LWK	AFR	467
NA19452	LWK	AFR	457
NA19454	LWK	AFR	496
NA19455	LWK	AFR	489
NA19456	LWK	AFR	470
NA19457	LWK	AFR	471
NA19461	LWK	AFR	484
NA19462	LWK	AFR	477
NA19463	LWK	AFR	477
NA19466	LWK	AFR	500
NA19467	LWK	AFR	477
NA19468	LWK	AFR	470
NA19471	LWK	AFR	426
NA19472	LWK	AFR	435
NA19473	LWK	AFR	471
NA19474	LWK	AFR	466
NA19475	LWK	AFR	480
NA19625	ASW	AFR	422
NA19648	MXL	AMR	339
NA19649	MXL	AMR	373
NA19651	MXL	AMR	316
NA19652	MXL	AMR	378
NA19654	MXL	AMR	346
NA19655	MXL	AMR	346
NA19657	MXL	AMR	366
NA19658	MXL	AMR	370
NA19661	MXL	AMR	398
NA19663	MXL	AMR	365
NA19664	MXL	AMR	370
NA19669	MXL	AMR	390
NA19670	MXL	AMR	355
NA19676	MXL	AMR	362
NA19678	MXL	AMR	373
NA19679	MXL	AMR	387
NA19681	MXL	AMR	351
NA19682	MXL	AMR	365
NA19684	MXL	AMR	355
NA19700	ASW	AFR	479
NA19701	ASW	AFR	442
NA19703	ASW	AFR	482
NA19704	ASW	AFR	465

NA19707	ASW	AFR	442
NA19711	ASW	AFR	494
NA19712	ASW	AFR	470
NA19713	ASW	AFR	419
NA19716	MXL	AMR	369
NA19717	MXL	AMR	370
NA19719	MXL	AMR	352
NA19720	MXL	AMR	414
NA19722	MXL	AMR	342
NA19723	MXL	AMR	391
NA19725	MXL	AMR	378
NA19726	MXL	AMR	361
NA19728	MXL	AMR	385
NA19729	MXL	AMR	364
NA19731	MXL	AMR	357
NA19732	MXL	AMR	335
NA19734	MXL	AMR	383
NA19735	MXL	AMR	379
NA19740	MXL	AMR	382
NA19741	MXL	AMR	362
NA19746	MXL	AMR	361
NA19747	MXL	AMR	337
NA19749	MXL	AMR	326
NA19750	MXL	AMR	355
NA19752	MXL	AMR	362
NA19755	MXL	AMR	345
NA19756	MXL	AMR	381
NA19758	MXL	AMR	368
NA19759	MXL	AMR	348
NA19761	MXL	AMR	356
NA19762	MXL	AMR	385
NA19764	MXL	AMR	376
NA19770	MXL	AMR	332
NA19771	MXL	AMR	338
NA19773	MXL	AMR	346
NA19774	MXL	AMR	368
NA19776	MXL	AMR	418
NA19777	MXL	AMR	362
NA19779	MXL	AMR	346
NA19780	MXL	AMR	333
NA19782	MXL	AMR	378
NA19783	MXL	AMR	340
NA19785	MXL	AMR	369
NA19786	MXL	AMR	341
NA19788	MXL	AMR	355
NA19789	MXL	AMR	418
NA19792	MXL	AMR	414
NA19794	MXL	AMR	365
NA19795	MXL	AMR	376
NA19818	ASW	AFR	455

NA19819	ASW	AFR	470
NA19834	ASW	AFR	449
NA19835	ASW	AFR	487
NA19900	ASW	AFR	456
NA19901	ASW	AFR	484
NA19904	ASW	AFR	468
NA19908	ASW	AFR	431
NA19909	ASW	AFR	489
NA19913	ASW	AFR	469
NA19914	ASW	AFR	456
NA19916	ASW	AFR	493
NA19917	ASW	AFR	480
NA19920	ASW	AFR	468
NA19921	ASW	AFR	461
NA19922	ASW	AFR	463
NA19923	ASW	AFR	413
NA19982	ASW	AFR	496
NA19984	ASW	AFR	499
NA20126	ASW	AFR	424
NA20127	ASW	AFR	471
NA20274	ASW	AFR	416
NA20276	ASW	AFR	442
NA20278	ASW	AFR	413
NA20281	ASW	AFR	462
NA20282	ASW	AFR	420
NA20287	ASW	AFR	456
NA20289	ASW	AFR	445
NA20291	ASW	AFR	450
NA20294	ASW	AFR	480
NA20296	ASW	AFR	443
NA20298	ASW	AFR	485
NA20299	ASW	AFR	442
NA20314	ASW	AFR	345
NA20317	ASW	AFR	410
NA20318	ASW	AFR	460
NA20320	ASW	AFR	473
NA20321	ASW	AFR	466
NA20332	ASW	AFR	453
NA20334	ASW	AFR	474
NA20339	ASW	AFR	453
NA20340	ASW	AFR	416
NA20342	ASW	AFR	440
NA20346	ASW	AFR	434
NA20348	ASW	AFR	486
NA20351	ASW	AFR	424
NA20355	ASW	AFR	442
NA20356	ASW	AFR	494
NA20357	ASW	AFR	473
NA20359	ASW	AFR	458
NA20362	ASW	AFR	453

NA20412	ASW	AFR	444
NA20502	TSI	EUR	360
NA20503	TSI	EUR	370
NA20504	TSI	EUR	347
NA20505	TSI	EUR	341
NA20506	TSI	EUR	325
NA20507	TSI	EUR	317
NA20508	TSI	EUR	348
NA20509	TSI	EUR	340
NA20510	TSI	EUR	328
NA20511	TSI	EUR	373
NA20512	TSI	EUR	358
NA20513	TSI	EUR	352
NA20514	TSI	EUR	344
NA20515	TSI	EUR	340
NA20516	TSI	EUR	373
NA20517	TSI	EUR	348
NA20518	TSI	EUR	346
NA20519	TSI	EUR	340
NA20520	TSI	EUR	345
NA20521	TSI	EUR	336
NA20522	TSI	EUR	325
NA20524	TSI	EUR	362
NA20525	TSI	EUR	318
NA20527	TSI	EUR	313
NA20528	TSI	EUR	380
NA20529	TSI	EUR	321
NA20530	TSI	EUR	339
NA20531	TSI	EUR	341
NA20532	TSI	EUR	355
NA20533	TSI	EUR	360
NA20534	TSI	EUR	377
NA20535	TSI	EUR	363
NA20536	TSI	EUR	391
NA20538	TSI	EUR	361
NA20539	TSI	EUR	335
NA20540	TSI	EUR	356
NA20541	TSI	EUR	379
NA20542	TSI	EUR	373
NA20543	TSI	EUR	369
NA20544	TSI	EUR	360
NA20581	TSI	EUR	363
NA20582	TSI	EUR	374
NA20585	TSI	EUR	324
NA20586	TSI	EUR	326
NA20587	TSI	EUR	350
NA20588	TSI	EUR	359
NA20589	TSI	EUR	332
NA20752	TSI	EUR	370
NA20753	TSI	EUR	368

NA20754	TSI	EUR	330
NA20755	TSI	EUR	369
NA20756	TSI	EUR	343
NA20757	TSI	EUR	359
NA20758	TSI	EUR	354
NA20759	TSI	EUR	367
NA20760	TSI	EUR	357
NA20761	TSI	EUR	350
NA20762	TSI	EUR	380
NA20763	TSI	EUR	351
NA20764	TSI	EUR	319
NA20765	TSI	EUR	364
NA20766	TSI	EUR	351
NA20767	TSI	EUR	346
NA20768	TSI	EUR	359
NA20769	TSI	EUR	347
NA20770	TSI	EUR	333
NA20771	TSI	EUR	357
NA20772	TSI	EUR	377
NA20773	TSI	EUR	369
NA20774	TSI	EUR	363
NA20775	TSI	EUR	342
NA20778	TSI	EUR	345
NA20783	TSI	EUR	333
NA20785	TSI	EUR	334
NA20786	TSI	EUR	366
NA20787	TSI	EUR	394
NA20790	TSI	EUR	351
NA20792	TSI	EUR	369
NA20795	TSI	EUR	337
NA20796	TSI	EUR	331
NA20797	TSI	EUR	365
NA20798	TSI	EUR	367
NA20799	TSI	EUR	366
NA20800	TSI	EUR	322
NA20801	TSI	EUR	366
NA20802	TSI	EUR	329
NA20803	TSI	EUR	360
NA20804	TSI	EUR	366
NA20805	TSI	EUR	323
NA20806	TSI	EUR	383
NA20807	TSI	EUR	359
NA20808	TSI	EUR	346
NA20809	TSI	EUR	351
NA20810	TSI	EUR	349
NA20811	TSI	EUR	343
NA20812	TSI	EUR	360
NA20813	TSI	EUR	367
NA20814	TSI	EUR	374
NA20815	TSI	EUR	349

NA20818	TSI	EUR	350
NA20819	TSI	EUR	365
NA20821	TSI	EUR	337
NA20822	TSI	EUR	344
NA20826	TSI	EUR	351
NA20827	TSI	EUR	300
NA20828	TSI	EUR	342
NA20832	TSI	EUR	333
NA20845	GIH	SAS	352
NA20846	GIH	SAS	332
NA20847	GIH	SAS	350
NA20849	GIH	SAS	360
NA20850	GIH	SAS	335
NA20851	GIH	SAS	318
NA20852	GIH	SAS	375
NA20853	GIH	SAS	327
NA20854	GIH	SAS	316
NA20856	GIH	SAS	342
NA20858	GIH	SAS	341
NA20859	GIH	SAS	350
NA20861	GIH	SAS	361
NA20862	GIH	SAS	345
NA20863	GIH	SAS	337
NA20864	GIH	SAS	341
NA20866	GIH	SAS	334
NA20867	GIH	SAS	367
NA20868	GIH	SAS	355
NA20869	GIH	SAS	333
NA20870	GIH	SAS	324
NA20872	GIH	SAS	378
NA20874	GIH	SAS	324
NA20875	GIH	SAS	334
NA20876	GIH	SAS	344
NA20877	GIH	SAS	367
NA20878	GIH	SAS	351
NA20881	GIH	SAS	368
NA20882	GIH	SAS	375
NA20884	GIH	SAS	365
NA20885	GIH	SAS	337
NA20886	GIH	SAS	352
NA20887	GIH	SAS	335
NA20888	GIH	SAS	334
NA20889	GIH	SAS	395
NA20890	GIH	SAS	325
NA20891	GIH	SAS	349
NA20892	GIH	SAS	340
NA20894	GIH	SAS	350
NA20895	GIH	SAS	349
NA20896	GIH	SAS	381
NA20897	GIH	SAS	366

NA20899	GIH	SAS	349
NA20900	GIH	SAS	387
NA20901	GIH	SAS	368
NA20902	GIH	SAS	376
NA20903	GIH	SAS	360
NA20904	GIH	SAS	352
NA20905	GIH	SAS	343
NA20906	GIH	SAS	365
NA20908	GIH	SAS	374
NA20910	GIH	SAS	353
NA20911	GIH	SAS	339
NA21086	GIH	SAS	350
NA21087	GIH	SAS	368
NA21088	GIH	SAS	364
NA21089	GIH	SAS	362
NA21090	GIH	SAS	358
NA21091	GIH	SAS	403
NA21092	GIH	SAS	385
NA21093	GIH	SAS	359
NA21094	GIH	SAS	358
NA21095	GIH	SAS	411
NA21097	GIH	SAS	353
NA21098	GIH	SAS	356
NA21099	GIH	SAS	357
NA21100	GIH	SAS	368
NA21101	GIH	SAS	343
NA21102	GIH	SAS	369
NA21103	GIH	SAS	362
NA21104	GIH	SAS	347
NA21105	GIH	SAS	398
NA21106	GIH	SAS	344
NA21107	GIH	SAS	354
NA21108	GIH	SAS	346
NA21109	GIH	SAS	357
NA21110	GIH	SAS	367
NA21111	GIH	SAS	352
NA21112	GIH	SAS	372
NA21113	GIH	SAS	351
NA21114	GIH	SAS	357
NA21115	GIH	SAS	380
NA21116	GIH	SAS	352
NA21117	GIH	SAS	340
NA21118	GIH	SAS	361
NA21119	GIH	SAS	358
NA21120	GIH	SAS	331
NA21122	GIH	SAS	349
NA21123	GIH	SAS	343
NA21124	GIH	SAS	333
NA21125	GIH	SAS	357
NA21126	GIH	SAS	344

NA21127	GIH	SAS	333
NA21128	GIH	SAS	397
NA21129	GIH	SAS	390
NA21130	GIH	SAS	359
NA21133	GIH	SAS	361
NA21135	GIH	SAS	326
NA21137	GIH	SAS	331
NA21141	GIH	SAS	342
NA21142	GIH	SAS	374
NA21143	GIH	SAS	331
NA21144	GIH	SAS	382
