# Supplementary material for: Nucleotide Composition of Ultra-Conserved Elements Shows Excess of GpC and Depletion of GG and CC Dinucleotides
Source: Genes (Basel). 2022 Nov 7;13(11):2053. doi: 10.3390/genes13112053 (PMC9690913; doi:10.3390/genes13112053)
Supplement: Supplementary file 1 [file genes-13-02053-s001.zip › File S1/NTcomposition.tar.gz!/NTcomposition/UCE_4mer_sorted.docx]

```
CGCG	0.000140	196
CGAC	0.000404	568
GACG	0.000438	615
GTCG	0.000447	628
GGCG	0.000453	636
CCCG	0.000456	641
CGTC	0.000456	641
CCGG	0.000458	644
CGCC	0.000467	656
GCGC	0.000483	678
CGGG	0.000487	684
CGTA	0.000488	685
ACCG	0.000499	701
TACG	0.000517	726
ACGC	0.000518	728
TCCG	0.000525	737
CGGA	0.000527	740
CCGC	0.000532	748
GCGT	0.000534	750
CGGT	0.000537	754
CCGA	0.000538	756
GCGG	0.000547	768
GCGA	0.000559	785
TCGG	0.000562	790
ACGA	0.000563	791
TCGC	0.000571	802
CGAG	0.000580	815
CTCG	0.000591	830
TCGA	0.000592	831
CCGT	0.000609	855
CGAA	0.000613	861
ACGG	0.000626	879
TTCG	0.000642	902
CGGC	0.000643	903
TCGT	0.000643	903
CACG	0.000651	915
CGAT	0.000651	915
ACGT	0.000658	924
GCCG	0.000658	924
TGCG	0.000690	969
CGCA	0.000693	973
ATCG	0.000694	975
CGTG	0.000694	975
AACG	0.000759	1066
CGCT	0.000804	1129
CGTT	0.000812	1140
AGCG	0.000842	1183
GGTC	0.001435	2016
GACC	0.001446	2031
GGAC	0.001477	2075
GTCC	0.001536	2158
GCCC	0.001650	2318
GGGG	0.001696	2382
GGGC	0.001703	2393
GGGT	0.001728	2428
GGCC	0.001740	2444
CCCC	0.001748	2455
ACCC	0.001753	2462
GATC	0.001966	2762
GTAC	0.001992	2798
TACC	0.002033	2856
CTAG	0.002035	2859
GTAG	0.002064	2899
GGTA	0.002078	2919
CACC	0.002144	3012
CTAC	0.002145	3014
GGTG	0.002268	3186
TAGG	0.002286	3212
CCTA	0.002290	3217
CCAC	0.002355	3308
GTGG	0.002440	3428
ACTC	0.002528	3552
ATCC	0.002551	3583
GAGT	0.002578	3622
GGAT	0.002607	3663
CCTC	0.002614	3672
TCCC	0.002640	3709
GAAC	0.002668	3748
GTTC	0.002669	3750
GACT	0.002686	3774
AGTC	0.002687	3775
GAGC	0.002691	3780
GGGA	0.002700	3793
GAGG	0.002717	3817
CCCA	0.002719	3819
GCTC	0.002763	3882
TGGG	0.002773	3895
GCAC	0.002783	3909
CTCC	0.002784	3911
GGTT	0.002797	3929
AACC	0.002808	3945
GGAG	0.002829	3974
CAAC	0.002835	3982
AGGG	0.002854	4010
CCCT	0.002860	4018
GTGC	0.002860	4018
ACCT	0.002898	4071
GTTG	0.002916	4097
AGAC	0.002996	4209
GTCT	0.003032	4259
AGGT	0.003036	4265
GCTA	0.003083	4331
ATAC	0.003103	4359
GTAT	0.003139	4410
ACTA	0.003161	4441
AGCC	0.003163	4443
TAGC	0.003170	4453
TAGT	0.003190	4481
AGGC	0.003207	4505
GGCT	0.003225	4531
GCCT	0.003230	4538
GCCA	0.003239	4550
TGGT	0.003271	4595
ACCA	0.003280	4608
TGGC	0.003349	4705
CCAG	0.003379	4747
CTGG	0.003391	4763
TATC	0.003425	4811
TGCC	0.003425	4811
CTAT	0.003433	4822
CCAA	0.003447	4843
GATA	0.003455	4854
ACAC	0.003485	4896
ATAG	0.003492	4906
GGCA	0.003492	4906
TTGG	0.003555	4994
CAGG	0.003625	5093
CCTG	0.003662	5144
CATC	0.003677	5165
CTTA	0.003684	5175
TAGA	0.003700	5198
TCAC	0.003777	5306
GTGT	0.003802	5341
TACT	0.003813	5357
GATG	0.003815	5359
TAAC	0.003817	5362
TCTA	0.003818	5364
GTGA	0.003836	5389
TAAG	0.003865	5429
AGTA	0.003878	5448
CTCA	0.003904	5484
AGGA	0.003924	5513
TGAC	0.003941	5536
GAGA	0.003941	5537
TCTC	0.003944	5541
GTTA	0.003961	5565
TGAG	0.003964	5569
CTTC	0.003970	5577
TCCA	0.003982	5594
TCCT	0.003986	5599
GAAG	0.004033	5665
CCAT	0.004033	5666
TGGA	0.004065	5710
ATGG	0.004074	5723
GTCA	0.004094	5751
CAAG	0.004143	5820
CTTG	0.004156	5838
GACA	0.004239	5955
TTCC	0.004266	5993
CATG	0.004302	6044
GGAA	0.004326	6077
AAGG	0.004399	6180
CACT	0.004402	6184
CCTT	0.004408	6193
CATA	0.004507	6331
AGTG	0.004515	6342
TGTC	0.004533	6368
CTCT	0.004534	6369
ATGC	0.004535	6371
AGAG	0.004539	6377
TATG	0.004547	6388
GCAT	0.004574	6425
AAGC	0.004668	6557
TTAC	0.004681	6576
GCTT	0.004705	6609
CTAA	0.004756	6681
TTAG	0.004762	6690
AACT	0.004803	6747
ACTT	0.004821	6772
TTGC	0.004859	6826
GTAA	0.004860	6828
AGTT	0.004868	6838
GCAA	0.004936	6934
CTGC	0.004947	6949
GCAG	0.004994	7015
GAAT	0.005000	7024
AAGT	0.005006	7032
TATA	0.005018	7049
AATC	0.005026	7060
GATT	0.005031	7067
ATCT	0.005040	7080
AGAT	0.005082	7139
ATTC	0.005107	7175
CAGT	0.005156	7243
CACA	0.005191	7293
CAGC	0.005194	7297
ACTG	0.005196	7299
GCTG	0.005196	7300
CTGA	0.005335	7495
TCAG	0.005382	7561
TGTG	0.005461	7672
AGCT	0.005508	7738
TACA	0.005547	7792
TGTA	0.005609	7879
CAGA	0.005753	8082
TCAA	0.005786	8128
ATCA	0.005806	8156
TCTG	0.005828	8187
ACAT	0.005873	8251
CAAT	0.005913	8307
TGAT	0.005918	8313
TTGA	0.005937	8340
ATGT	0.005994	8420
AAAC	0.005994	8421
AAGA	0.006003	8433
ACAG	0.006005	8436
ATTG	0.006010	8443
TCTT	0.006012	8446
TGCT	0.006194	8702
GTTT	0.006199	8708
AGCA	0.006202	8713
CTGT	0.006214	8730
ATAT	0.006528	9170
ACAA	0.006540	9187
TCAT	0.006690	9398
ATGA	0.006706	9421
AGAA	0.006790	9539
TTGT	0.006834	9601
TGCA	0.006890	9679
AACA	0.006937	9745
TTCT	0.007010	9848
TGTT	0.007257	10194
CAAA	0.007818	10983
TTCA	0.007853	11032
TGAA	0.007998	11235
AAAG	0.008032	11284
GAAA	0.008042	11298
TTTC	0.008045	11302
TTTG	0.008120	11407
CTTT	0.008134	11427
ATAA	0.008621	12111
AATG	0.008653	12156
CATT	0.008698	12219
TTAT	0.008714	12241
AATA	0.008771	12322
TATT	0.008820	12390
ATTA	0.010635	14940
TAAT	0.010724	15065
TAAA	0.010900	15313
TTTA	0.010930	15355
TTAA	0.011036	15503
AATT	0.011945	16781
ATTT	0.012765	17932
AAAT	0.012767	17935
AAAA	0.014041	19725
TTTT	0.014334	20137
```
