# Supplementary material for: Nucleotide Composition of Ultra-Conserved Elements Shows Excess of GpC and Depletion of GG and CC Dinucleotides
Source: Genes (Basel). 2022 Nov 7;13(11):2053. doi: 10.3390/genes13112053 (PMC9690913; doi:10.3390/genes13112053)
Supplement: Supplementary file 1 [file genes-13-02053-s001.zip › File S1/NTcomposition.tar.gz!/NTcomposition/Table3.docx]

| **Oligonucleotides** | **UCE sequences** | | **Chromosome #1** | |
| --- | --- | --- | --- | --- |
|  | **Relative**  **Frequency** | **Number**  **occurrences** | **Relative**  **Frequency** | **Number**  **occurrences** |
| 1-mer  (nucleotides) |  |  |  |  |
| A | 0.314 | 445884 | 0.291 | 67070277 |
| T | 0.317 | 449114 | 0.292 | 67244164 |
| C | 0.183 | 260157 | 0.208 | 48055043 |
| G | 0.185 | 262642 | 0.209 | 48111528 |
|  |  |  |  |  |
| 2-mer  (dinucleotides) |  |  |  |  |
| AA | 0.1105 | 156199 | 0.0950 | 21901540 |
| AT | 0.0915 | 129314 | 0.0743 | 17121783 |
| AC | 0.0484 | 68447 | 0.0503 | 11598278 |
| AG | 0.0642 | 90715 | 0.0714 | 16448644 |
| TA | 0.0753 | 106391 | 0.0631 | 14554789 |
| TT | 0.1115 | 157604 | 0.0957 | 22048241 |
| TC | 0.0548 | 77483 | 0.0601 | 13844699 |
| TG | 0.0753 | 106452 | 0.0729 | 16796378 |
| CA | 0.0740 | 104633 | 0.0728 | 16768284 |
| CT | 0.0641 | 90607 | 0.0713 | 16444797 |
| CC | 0.0362 | 51183 | 0.0541 | 12466763 |
| CG | 0.0090 | 12748 | 0.0103 | 2375159 |
| GA | 0.0547 | 77358 | 0.0601 | 13845615 |
| GT | 0.0498 | 70436 | 0.0505 | 11629291 |
| GC | 0.0440 | 62159 | 0.0440 | 10145272 |
| GG | 0.0366 | 51737 | 0.0542 | 12491312 |
|  |  |  |  |  |
| 3-mer  (triplets) |  |  |  |  |
| AAA | 0.04083 | 57532 | 0.03695 | 8516543 |
| AAT | 0.03436 | 48424 | 0.02374 | 5470905 |
| AAC | 0.01531 | 21576 | 0.01446 | 3332435 |
| AAG | 0.02008 | 28302 | 0.01988 | 4581648 |
| ATA | 0.02173 | 30622 | 0.01942 | 4475100 |
| ATT | 0.03446 | 48554 | 0.02387 | 5500468 |
| ATC | 0.01408 | 19839 | 0.01317 | 3035996 |
| ATG | 0.02130 | 30019 | 0.01783 | 4110209 |
| ACA | 0.02191 | 30869 | 0.01976 | 4553751 |
| ACT | 0.01571 | 22142 | 0.01620 | 3732934 |
| ACC | 0.00843 | 11883 | 0.01182 | 2725309 |
| ACG | 0.00237 | 3335 | 0.00254 | 586276 |
| AGA | 0.01941 | 27353 | 0.02235 | 5150760 |
| AGT | 0.01595 | 22472 | 0.01614 | 3719675 |
| AGC | 0.01573 | 22161 | 0.01439 | 3317232 |
| AGG | 0.01304 | 18369 | 0.01849 | 4260968 |
| TAA | 0.02926 | 41225 | 0.01986 | 4577976 |
| TAT | 0.02179 | 30705 | 0.01941 | 4472951 |
| TAC | 0.01191 | 16780 | 0.01103 | 2542958 |
| TAG | 0.01235 | 17407 | 0.01285 | 2960898 |
| TTA | 0.02917 | 41106 | 0.01983 | 4571528 |
| TTT | 0.04143 | 58385 | 0.03724 | 8583142 |
| TTC | 0.01978 | 27875 | 0.01974 | 4548877 |
| TTG | 0.02117 | 29834 | 0.01885 | 4344678 |
| TCA | 0.02163 | 30484 | 0.01962 | 4522569 |
| TCT | 0.01962 | 27643 | 0.02226 | 5129424 |
| TCC | 0.01114 | 15703 | 0.01587 | 3657040 |
| TCG | 0.00237 | 3345 | 0.00232 | 535651 |
| TGA | 0.02180 | 30724 | 0.01947 | 4486632 |
| TGT | 0.02284 | 32190 | 0.01989 | 4584113 |
| TGC | 0.01720 | 24240 | 0.01457 | 3357313 |
| TGG | 0.01346 | 18970 | 0.01895 | 4368306 |
| CAA | 0.02069 | 29151 | 0.01861 | 4288540 |
| CAT | 0.02117 | 29829 | 0.01788 | 4120946 |
| CAC | 0.01239 | 17466 | 0.01521 | 3506405 |
| CAG | 0.01976 | 27845 | 0.02105 | 4852390 |
| CTA | 0.01237 | 17425 | 0.01276 | 2941433 |
| CTT | 0.01995 | 28116 | 0.02011 | 4634644 |
| CTC | 0.01186 | 16714 | 0.01760 | 4057534 |
| CTG | 0.01990 | 28042 | 0.02087 | 4811169 |
| CCA | 0.01322 | 18634 | 0.01879 | 4330820 |
| CCT | 0.01298 | 18288 | 0.01854 | 4273302 |
| CCC | 0.00781 | 11006 | 0.01385 | 3193020 |
| CCG | 0.00214 | 3016 | 0.00291 | 669612 |
| CGA | 0.00225 | 3173 | 0.00227 | 523798 |
| CGT | 0.00245 | 3453 | 0.00259 | 597422 |
| CGC | 0.00211 | 2967 | 0.00251 | 579316 |
| CGG | 0.00220 | 3096 | 0.00293 | 674618 |
| GAA | 0.01974 | 27818 | 0.01960 | 4518460 |
| GAT | 0.01425 | 20080 | 0.01326 | 3056974 |
| GAC | 0.00881 | 12409 | 0.00962 | 2216474 |
| GAG | 0.01194 | 16823 | 0.01759 | 4053693 |
| GTA | 0.01206 | 16988 | 0.01114 | 2566721 |
| GTT | 0.01574 | 22177 | 0.01445 | 3329970 |
| GTC | 0.00911 | 12839 | 0.00956 | 2202280 |
| GTG | 0.01295 | 18242 | 0.01532 | 3530308 |
| GCA | 0.01729 | 24365 | 0.01458 | 3361131 |
| GCT | 0.01575 | 22187 | 0.01436 | 3309131 |
| GCC | 0.00879 | 12391 | 0.01255 | 2891387 |
| GCG | 0.00213 | 2995 | 0.00253 | 583618 |
| GGA | 0.01124 | 15843 | 0.01599 | 3684403 |
| GGT | 0.00857 | 12082 | 0.01184 | 2728078 |
| GGC | 0.00891 | 12562 | 0.01255 | 2891408 |
| GGG | 0.00784 | 11045 | 0.01383 | 3187415 |
