# Supplementary material for: Nucleotide Composition of Ultra-Conserved Elements Shows Excess of GpC and Depletion of GG and CC Dinucleotides
Source: Genes (Basel). 2022 Nov 7;13(11):2053. doi: 10.3390/genes13112053 (PMC9690913; doi:10.3390/genes13112053)
Supplement: Supplementary file 1 [file genes-13-02053-s001.zip › File S1/UCNEperlPrograms.tar!/UCNEperlPrograms/UCEperl_instructions.docx]

**PERL PROGRAMS INSTRUCTION MANUAL**

All SNPs inside UCEdb are stored inside ***SNPsUCEvcf2_$chr*** files, created by *UCEsnps.pl* program, which command line is the following:

perl UCEsnps.pl X

perl UCEsnps.pl 22

In these two examples the arguments (X or 22) are chromosome numbers.

Distribution of SNPs by frequency inside UCEs created by UCEsnpINDapr18.pl program, for which command line is the following:

perl UCEsnpINDapr18.pl

This program takes as input files the ***SNPsUCEvcf2_$chr*** (from previous step) and also two tables from 1000G database ("2504indsApr18" and ”igsr_samples.tsv" ), that have information about individual identifiers, population names and regions/continents.

Finally, program 1000GfreqSNPsUCE.pl is the modification of UCEsnpINDapr18.pl

Distribution of meiotic recombination rates inside UCE created by *GeneticMap_AF.pl* program using command line:

perl GeneticMap_AF.pl >RecRateDistr.txt

Random expectation for UCE command line:

perl RandomPositionsForRecombination.pl >RandTable

This initiates random positions for UCE has been initiated. Then, by the following commands they have been finished taking into processing RandTable:

perl RandomUCE.pl >test2

sort -n -k1 -k2 test2 >RandUCEcoord2.txt

Finally the program calculates distributions of RR inside random-UCEs

perl GeneticMap_AFrand.pl >RecRateDistrRand2.txt

For computation of oligonucleotide composition of fasta-formated nucleotide sequences ust the following(command line:

perl NTcomposition.pl Database.fasta.txt output_filename 8

The first argument is your database of nucleotide sequences, the second argument is the name the output results will be saved in, and the third argument (8) is the size of the largest oligonucleotides to be computed (in this example 8-mers).

**Protocol for Bootstrap statistics:**

STEP-1 creates 1000 Bootstrap.$n files ($n = 1..1000)

perl BootstrapUCNE.pl UCNEfasta_sorted.txt

STEP-2: creates 1000 NT-composition files NTcompRAND$n (e.g. NTcompRAND83). A user should also have ***NTcomp.pl*** program in the same folder

perl startNTcompRAND.pl

STEP-3: calculates Genomic Signatures for 1000 NTcompRAND$n files and sort them. Prints out the Genomic Signature for 250, 500, and 750 sorted values. Standard error will be value for 750^th^ – value for 250^th^ divided by 2.

perl GenomicSignature.pl

**PROTOCOLS FOR UCE PROJECT (original journal records for internal usage)**

February 26, 2022

```
cd
  506  cd Documents/
  507  ls
  508   cd UCEs2022Ali/
  509  ls
  510  ls -l
  511  cp  NTcomposition.pl UCE_database
  512  cd UCE_database
  513  ls
  514  perl NTcomposition.pl hg19_UCNEs.fasta.txt COMP_UCEdbNAR 8
  515  ls
  516  more COMP_UCEdbNAR.tbl
  517  more COMP_UCEdbNAR.tbl
  518  ls -l
  519  more COMP_UCEdbNAR.tbl
  520  more COMP_UCEdbNAR
```

I got table of 1- to 8-mer distirubtion table inside UCEdatabase (file COMP_UCEdbNAR)

List of over-abundant 8-mer oligos with GC-comp >=50%

```
GCTGCTGT	0.0000511719	71
TGCTGCAG	0.0000511719	71
TTGCAGCT	0.0000511719	71
ACACAGCA	0.0000518926	72
ACTGCTGA	0.0000518926	72
AGCTGCAA	0.0000518926	72
AGCTGCTT	0.0000518926	72
CAGCTTCA	0.0000518926	72
CTGCTGTG	0.0000518926	72
TGCAGCTT	0.0000518926	72
TGCTGTGT	0.0000518926	72
TGTCAGAG	0.0000518926	72
AGCAGCAT	0.0000526134	73
CTCTCTCT	0.0000526134	73
GCAGCAGC	0.0000526134	73
GGCAGCTG	0.0000526134	73
TGGCAGCT	0.0000526134	73
TGTCAGCA	0.0000526134	73
CACTGCTG	0.0000533341	74
CAGCAGAA	0.0000533341	74
CTGCTGCT	0.0000533341	74
CTGTCAGA	0.0000533341	74
CTGTCAGT	0.0000533341	74
TGCTGACA	0.0000533341	74
TTGTCAGC	0.0000533341	74
ACTGACAG	0.0000540548	75
ATGCAGCT	0.0000540548	75
GGGGAAAA	0.0000540548	75
TGTGACAG	0.0000540548	75
AATGCAGC	0.0000547756	76
AGCTGCAG	0.0000547756	76
CAGAGCTG	0.0000547756	76
CTTGTCAG	0.0000547756	76
GCTGTCAT	0.0000547756	76
TTCTGCAG	0.0000547756	76
AACTGCTG	0.0000554963	77
ACAGCTGC	0.0000554963	77
CTGCTGTT	0.0000554963	77
CTGTCACT	0.0000554963	77
GCAGCTGC	0.0000554963	77 palindrom
TGTCACTG	0.0000554963	77
AGCTGTGA	0.0000562170	78
TCTCTCTG	0.0000562170	78
TGCAGATG	0.0000562170	78
TGCAGTGA	0.0000562170	78
TTCAGCAG	0.0000562170	78
TTCAGCTG	0.0000562170	78
TTGCTGCA	0.0000562170	78
CTGCAGAA	0.0000569378	79
TACAGCTG	0.0000569378	79
TGCAGCTG	0.0000569378	79
TTTGCAGC	0.0000569378	79
ACAGCAGC	0.0000576585	80
AGCAGATG	0.0000576585	80
CTGCAGTT	0.0000576585	80
CTGCAGTG	0.0000583792	81
TCAGCAGA	0.0000583792	81
AGCTGCTG	0.0000591000	82
CAGCAGCT	0.0000591000	82
ACAGATGG	0.0000598207	83
CATCTGCT	0.0000598207	83
TGACAGCA	0.0000605414	84
AACAGCAG	0.0000612621	85
AGCAGCTG	0.0000612621	85
CTGCTGAA	0.0000612621	85
GCTGTCAG	0.0000612621	85
TTCTGCTG	0.0000612621	85
AAAGGCAG	0.0000619829	86
AAGCAGCA	0.0000619829	86
GCAGCTGT	0.0000627036	87
AAAGCAGC	0.0000641451	89
CACAGCAG	0.0000641451	89
CAGCAGCA	0.0000641451	89
CAGCTGCA	0.0000641451	89
CAGCTGTG	0.0000648658	90
TGTCAGCT	0.0000663073	92
TTTTCCCC	0.0000663073	92
CAGCTGCT	0.0000670280	93
CAGCTGTT	0.0000677487	94
TGACAGCT	0.0000699109	97
AACAGCTG	0.0000706316	98
AGCAGCAG	0.0000706316	98
AGCTGTCA	0.0000720731	100
ACAGCTGT	0.0000749560	104
TGCTGTCA	0.0000749560	104
```

History:

```
  537  grep CAGCTG  UCE_7merSORTED
  538  ls -l
  539  grep ACAGCTGT COMP_UCEdbNAR
  540  grep TCAGCTGA COMP_UCEdbNAR
  541  vi get7mer.pl
  542  perl get7mer.pl >UCE_8mer
  543  more UCE_8mer
  544  ls
  545  cat -vent UCE_8mer
  546  ls
  547  freqOLIGO.pl
  548  vi freqOLIGO.pl
  549  vi freqOLIGO.pl
  550  perl freqOLIGO.pl
  551  perl freqOLIGO.pl >test
  552  sort -k3 -n test
```

**CONCLUSIONS:**

1) I found enrichment with GCA and GCT repeats (CTG CAG the same triplets)
2) Genome signatures of UCE: GC=1.30; CpG = 0.27; (CpG is average for humans and avoids CpG-islands)

CA =1.29 (higher) CT = 1.10 (lower). GT = 0.85 (average); AG= 1.11; TG= 1.28

CONCLUSION: GC is overabundant at the expense of CC and GG depletion

Below is computation of genomic signatures for UCE and human genome

```
Alexeis-iMac:UCE_database afedorov$ more COMP_UCEdbNAR
A       0.314   445884
T       0.317   449114
C       0.183   260157
G       0.185   262642
AA      0.1105  156199   AAsign =1.12
AT      0.0915  129314	  ATsign= 0.92
AC      0.0484  68447	  ACsign= 0.84
AG      0.0642  90715	  AGsign= 1.11
TA      0.0753  106391	  TAsign= 0.76
TT      0.1115  157604	  TTsign= 1.11
TC      0.0548  77483	  TCsign= 0.94
TG      0.0753  106452	  TGsign= 1.28
CA      0.0740  104633	  CAsign= 1.29
CT      0.0641  90607	  CTsing= 1.10
CC      0.0362  51183	  CCsign= 1.08
CG      0.0090  12748	  CGsign= 0.27
GA      0.0547  77358	  GAsign= 0.94
GT      0.0498  70436	  GTsign= 0.85
GC      0.0440  62159	  GCsign= 1.30
GG      0.0366  51737	  GGsign= 1.07

-bash-4.1$ more NTcompositionCHR16. (calculated on BPGN for entire chr16)
A	0.276	22558319
T	0.278	22774906
C	0.222	18172742
G	0.224	18299976
AA	0.0860	7033382	AAsign= 1.13
AT	0.0667	5457447	ATsign= 0.88
AC	0.0505	4134271	ACsign= 0.82
AG	0.0725	5933209  AGsign= 1.17
TA	0.0539	4406479	TAsign= 0.70
TT	0.0879	7191170	TTsign= 1.14
TC	0.0609	4985535  TCsign= 0.99
TG	0.0757	6191722  TGsign= 1.22
CA	0.0748	6119536	CA = 1.22
CT	0.0724	5923550	CTsign= 1.17
CC	0.0609	4978757	CCsign= 1.24
CG	0.0141	1150891  CGsign= 0.284
GA	0.0611	4998916	GAsign= 0.99
GT	0.0514	4202733	GTsign= 0.83
GC	0.0498	4074175   GCsign=1.00
GG	0.0614	5024149	GGsign= 1.22
```

Genomic signatures in human ( from L7_abpg2015 my old calculations)

AA 1.16	GA 0.98

AC 0.80	GC 0.98

AG 1.19	GG 1.26

AT 0.85	GT 0.79

CA 1.15	TA 0.72

CC 1.27	TC 0.97

CG 0.36	TG 1.17

CT 1.19	TT 1.16

Table for slide

**Dinucleotide	genome	UCEs**

```
CG			0.28		0.27
GC			1.00		1.30
TA			0.70		0.76
AT			0.88		0.92
CC/GG		1.23		1.08
TT/AA		1.14		1.12
TG/CA		1.22		1.28
AG/CT		1.17		1.10
AC/GT		0.82		0.84
GA/TC		0.99		0.94
```

**NEXT TASK:**

IS THERE ANY DIFFERENCES BETWEEN UCE DNA STRANDS? For example, one strand may be Pu-rich, while other will be Pu-poor. I will generate the new program CompareStrands.pl, which counts NTs difference between UCE seqs and their complementary strands. The differences will be in nts and di-nts. Then I will some the modules for the differences for all UCEs in the DB.

```
#!/usr/local/perl
@NT=('A', 'T', 'G', 'C');
$A=$T=$C=$G=0; $Pu=$Ket=0;  $L=0;
@diNT =();
for $x (0..3) {
        for $y (0..3) {
                $di = $NT[$x] . $NT[$y];
                push(@diNT, $di);
        }
}
$c=0;
$/='>';
open(IN, "hg19_UCNEs.fasta.txt");
while(<IN>) {
        $end =chop; chomp;
        $c++;
        $seq =$comp ='';
        @line=split(/\n/, $_);
        for $x (1..$#line) { $seq .= $line[$x];}
        $comp= reverse($seq);
        $L+=length($seq);
        $comp =~tr/AGTC/TCAG/;
        if ($c ==2) {print "$#line \n$seq \n\n$comp \n";}
        @S=split(//,$seq);
        @C=split(//,$comp);
        %ntS =%ntC=();
        for $x (0..$#S) {
                $ntS{$S[$x]}++;
                $ntC{$C[$x]}++;
        }
        foreach $n (@NT) {
                ${$n} +=abs($ntS{$n} - $ntC{$n});
        }
        $Pu +=abs($ntS{'A'} +$ntS{'G'} -$ntC{'A'} -$ntC{'G'});
        $Ket +=abs($ntS{'A'} +$ntS{'C'} -$ntC{'A'} -$ntC{'C'});
        print "$seq\n$comp\n$A \t$C \t $Pu \t$Ket\n\n";
}
print "total length is $L \n";
```

**CONCLUSION:**

I do not see significant difference between the strands (T vs A, G vs C, or Pu vs Py, or Keto vs Amino)

**March 1, 2022**

HISTORY from yesterday:

```
  589  more hg19_UCNEs.fasta.txt
  590  cat -vent  hg19_UCNEs.fasta.txt |head
  591  vi CompareStrands.pl
  592  perl CompareStrands.pl
  593  vi CompareStrands.pl
  594  perl CompareStrands.pl
  595  cat -vent  hg19_UCNEs.fasta.txt |head
  596  vi CompareStrands.pl
  597  perl CompareStrands.pl
  598  vi CompareStrands.pl
  599  perl CompareStrands.pl
  600  vi CompareStrands.pl
  601  perl CompareStrands.pl
  602  vi CompareStrands.pl
  603  perl CompareStrands.pl
  604  vi CompareStrands.pl
  605  perl CompareStrands.pl
  606  vi CompareStrands.pl
  607  perl CompareStrands.pl
  608  vi CompareStrands.pl
  609  perl CompareStrands.pl
  610  vi CompareStrands.pl
  611  perl CompareStrands.pl
  612  ls
  613  wc hg19_UCNE_coord.bed
  614  vi CompareStrands.pl
  615  perl CompareStrands.pl
  616  vi CompareStrands.pl
  617  vi CompareStrands.pl
  618  cp CompareStrands.pl CompareStrandsTRIPLETS.pl
  619  vi CompareStrandsTRIPLETS.pl
  620  vi CompareStrandsTRIPLETS.pl
  621  perl CompareStrandsTRIPLETS.pl
  622  vi CompareStrandsTRIPLETS.pl
  623  perl CompareStrandsTRIPLETS.pl
  624  vi CompareStrandsTRIPLETS.pl
  625  perl CompareStrandsTRIPLETS.pl
  626  history
```

UCE_4mer_sorted:

```
AAGC	0.004668	6557
TTAC	0.004681	6576
GCTT	0.004705	6609
CTAA	0.004756	6681
TTAG	0.004762	6690
AACT	0.004803	6747
ACTT	0.004821	6772
TTGC	0.004859	6826
GTAA	0.004860	6828
AGTT	0.004868	6838
GCAA	0.004936	6934
CTGC	0.004947	6949
GCAG	0.004994	7015
GAAT	0.005000	7024
AAGT	0.005006	7032
TATA	0.005018	7049
AATC	0.005026	7060
GATT	0.005031	7067
ATCT	0.005040	7080
AGAT	0.005082	7139
ATTC	0.005107	7175
CAGT	0.005156	7243
CACA	0.005191	7293
CAGC	0.005194	7297
ACTG	0.005196	7299
GCTG	0.005196	7300
CTGA	0.005335	7495
TCAG	0.005382	7561
TGTG	0.005461	7672
AGCT	0.005508	7738
TACA	0.005547	7792
TGTA	0.005609	7879
CAGA	0.005753	8082
TCAA	0.005786	8128
ATCA	0.005806	8156
TCTG	0.005828	8187
ACAT	0.005873	8251
CAAT	0.005913	8307
TGAT	0.005918	8313
TTGA	0.005937	8340
ATGT	0.005994	8420
AAAC	0.005994	8421
AAGA	0.006003	8433
ACAG	0.006005	8436
ATTG	0.006010	8443
TCTT	0.006012	8446
TGCT	0.006194	8702
GTTT	0.006199	8708
AGCA	0.006202	8713
CTGT	0.006214	8730
ATAT	0.006528	9170
ACAA	0.006540	9187
TCAT	0.006690	9398
ATGA	0.006706	9421
AGAA	0.006790	9539
TTGT	0.006834	9601
TGCA	0.006890	9679 Most frequent 4mer with 2 G/C nucleotides
AACA	0.006937	9745
TTCT	0.007010	9848
TGTT	0.007257	10194
CAAA	0.007818	10983
TTCA	0.007853	11032
TGAA	0.007998	11235
AAAG	0.008032	11284
GAAA	0.008042	11298
TTTC	0.008045	11302
TTTG	0.008120	11407
CTTT	0.008134	11427
ATAA	0.008621	12111
AATG	0.008653	12156
CATT	0.008698	12219
TTAT	0.008714	12241
AATA	0.008771	12322
TATT	0.008820	12390
ATTA	0.010635	14940
TAAT	0.010724	15065
TAAA	0.010900	15313
TTTA	0.010930	15355
TTAA	0.011036	15503
AATT	0.011945	16781
ATTT	0.012765	17932
AAAT	0.012767	17935
AAAA	0.014041	19725
TTTT	0.014334	20137
Alexeis-iMac:UCE_database afedorov$ sort -k3 -n UCE_4mer >UCE_4mer_sorted
```

**Next Idea:** study multiple alignments for association of mutations with GpC dinucleotides.

**April 21, 2022**

This project has been continued in the IBC2022 class during March and April.

HISTORY:

```
  255  mkdir UCE2022
  256  cd UCE2022/
  257  ls
  258  vi coordinates.pl
  259  perl coordinates.pl
  260  perl coordinates.pl |sort -n -k1 -k2
  261  ls
  262  perl coordinates.pl >hg19_UCNE_coord2.bed
  263  ls -l
  264  more hg19_UCNEs.fasta.txt
  265  ls
  266  more hg19_UCNE_coord.bed
  267  ls
  268  cd
  269  ls
  270  cd UCE2022/
  271  ls -l
  272  more hg19_UCNEs.fasta.txt
  273  ls
  274  grep >  hg19_UCNEs.fasta.txt|wc
  275  grep '>'  hg19_UCNEs.fasta.txt|wc
  276  ls
  277  grep '>'  hg19_UCNEs.fasta.txt
  278  more  hg19_UCNEs.fasta.txt
  279  history
  280  ls -l
  281  cd
  282  ls
  283  chmod 755 -Rf UCE2022/
  284  ls
  285  cd UCE2022/
  286  ls -l
  287  grep '>' hg19_UCNEs.fasta.txt
  288  grep '>' hg19_UCNEs.fasta.txt |wc
  289  ls
  290  more hg19_UCNEs.fasta.txt
  291  ls
  292  more hg19_UCNE_coord.bed
  293  ls
  294  more hg19_UCNE_coord2.bed
  295  ls
  296  sort -k1 -n -k2 hg19_UCNE_coord2.bed
  297  sort -k1 -n -k2 hg19_UCNE_coord2.bed >hg19_UCNE_coord2.bed_sorted
  298  ls
  299  more hg19_UCNE_coord2.bed_sorted
  300  ls
  301  vi UCEprog1.pl
  302  perl UCEprog1.pl
  303  vi UCEprog1.pl
  304  perl UCEprog1.pl
  305  ls -l
  306  wc hg19_UCNE_coord2.bed_sorted
  307  grep '>' hg19_UCNEs.fasta.txt
  308  grep '>' hg19_UCNEs.fasta.txt |wc
  309  vi UCEprog1.pl
  310  ls
  311  vi UCEprog1.pl
  312  perl UCEprog1.pl
  313  vi UCEprog1.pl
  314  perl UCEprog1.pl
  315  vi UCEprog1.pl
  316  perl UCEprog1.pl
  317  vi UCEprog1.pl
  318  perl UCEprog1.pl
  319  ls
  320  more hg19_UCNEs.fasta.txt
  321  vi UCEprog1.pl
  322  perl UCEprog1.pl
  323  vi UCEprog1.pl
  324  ls
  325  cat -vent hg19_UCNEs.fasta.txt
  326  vi UCEprog1.pl
  327  perl UCEprog1.pl
  328  vi UCEprog1.pl
  329  perl UCEprog1.pl
  330  vi UCEprog1.pl
  331  perl UCEprog1.pl
  332  vi UCEprog1.pl
  333  perl UCEprog1.pl
  334  vi UCEprog1.pl
  335  perl UCEprog1.pl
  336  ls
  337  vi UCEprog1.pl
  338  perl UCEprog1.pl
  339  vi UCEprog1.pl
  340  perl UCEprog1.pl
  341  perl UCEprog1.pl > UCEfasta_sorted.txt
  342  ls
  343  more UCEfasta_sorted.txt
  344  vi UCEprog1.pl
  345  perl UCEprog1.pl > UCEfasta_sorted.txt
  346  ls
  347  more  UCEfasta_sorted.txt
  348  ls
  349   exit
  350  ls
  351  ls -l *gz
  352  ls
  353   cd UCE2022/
  354  ls
  355  tar -xvf genetic_map_HapMapII_GRCh37.tar
  356  ls
  357  mkdir GeneticMap
  358  ls
  359  mv genetic* GeneticMap/
  360  ls
  361  cd GeneticMap/
  362  ls
  363  more genetic_map_GRCh37_chr10.txt
  364  cat -vent genetic_map_GRCh37_chr10.txt
  365  more genetic_map_GRCh37_chr10.txt
  366  ls
  367  vi GeneticMap.pl
  368  perl GeneticMap.pl
  369  vi GeneticMap.pl
  370  perl GeneticMap.pl
  371  vi GeneticMap.pl
  372  exit
  373  ls
  374  exit
  375  ls
  376  cd UCE2022/
  377  ls
  378  cd GeneticMap/
  379  ls
  380  more genetic_map_GRCh37_chr1.txt
  381  more genetic_map_GRCh37_chr16.txt
  382  ls -l
  383  vi GeneticMap.pl
  384  perl GeneticMap.pl
  385  head genetic_map_GRCh37_chr16.txt
  386  vi GeneticMap.pl
  387  perl GeneticMap.pl
  388  vi GeneticMap.pl
  389  perl GeneticMap.pl
  390  vi genetic_map_GRCh37_chr16.txt
  391  vi GeneticMap.pl
  392  perl GeneticMap.pl
  393  more genetic_map_GRCh37_chr16.txt
  394  history
  395  exit
  396  ls
  397  cd UCE2022/
  398  ls
  399  vi UCEprog1.pl
  400  ls -l *pl
  401  vi coordinates.pl
  402  cd GeneticMap/
  403  ls
  404  vi GeneticMap.pl
  405  cd ..
  406  ls
  407  ls -l
  408  more hg19_UCNE_coord2.bed_sorted
  409  ls
  410  pwd
  411  cd GeneticMap/
  412  ls
  413  vi GeneticMap.pl
  414  perl GeneticMap.pl
  415  cd ..
  416  ls -l
  417  vi coordinates.pl
  418  vi UCEprog1.pl
  419  more hg19_UCNE_coord2.bed_sorted
  420  ls
  421  cd GeneticMap/
  422  ls
  423  perl GeneticMap.pl
  424  vi GeneticMap.pl
  425  perl GeneticMap.pl
  426  vi GeneticMap.pl
  427  exit
  428  ls
  429  cd UCE2022/
  430  ls
  431  cd GeneticMap/
  432  ls
  433  vi GeneticMap.pl
  434  cd
  435  ls
  436  cd IBC1_PROGRAMS
  437  ls
  438  exit
  439  ls
  440  cd UCE2022/
  441  ls
  442  cd GeneticMap/
  443  ls
  444  vi GeneticMap.pl
  445  ls
  446  cd UCE2022/
  447  ls
  448  cd GeneticMap/
  449  ls
  450  vi GeneticMap.pl
  451  pwd
  452  vi GeneticMap.pl
  453  perl GeneticMap.pl
  454  vi GeneticMap.pl
  455  perl GeneticMap.pl
  456  ls
  457  tail genetic_map_GRCh37_chr16.txt
  458  grep 80424178 genetic_map_GRCh37_chr16.txt
  459  vi GeneticMap.pl
  460  perl GeneticMap.pl
  461  vi GeneticMap.pl
  462  perl GeneticMap.pl
  463  vi GeneticMap.pl
  464  perl GeneticMap.pl
  465  vi GeneticMap.pl
  466  perl GeneticMap.pl
  467  exit
  468  ls
  469  cd UCE2022/
  470  cd GeneticMap/
  471  ls
  472  cp GeneticMap.pl GeneticMap_AF.pl
  473  vi GeneticMap_AF.pl
  474  perl GeneticMap_AF.pl
  475  vi GeneticMap_AF.pl
  476  perl GeneticMap_AF.pl
  477  vi GeneticMap_AF.pl
  478  perl GeneticMap_AF.pl
  479  vi GeneticMap_AF.pl
  480  perl GeneticMap_AF.pl
  481  vi GeneticMap_AF.pl
  482  less ../hg19_UCNE_coord2.bed_sorted
  483  tail ../hg19_UCNE_coord2.bed_sorted
  484  vi GeneticMap_AF.pl
  485  perl GeneticMap_AF.pl
  486  ls
  487  grep 27213199 genetic_map_GRCh37_chr22.txt
  488  grep  16377650  genetic_map_GRCh37_chr21.txt
  489  grep 52105972 genetic_map_GRCh37_chr20.txt
  490  vi GeneticMap_AF.pl
  491  perl GeneticMap_AF.pl
  492  vi GeneticMap_AF.pl
  493  perl GeneticMap_AF.pl
  494  vi GeneticMap_AF.pl
  495  perl GeneticMap_AF.pl
  496  vi GeneticMap_AF.pl
  497  perl GeneticMap_AF.pl
  498  vi GeneticMap_AF.pl
  499  perl GeneticMap_AF.pl
  500  ls
  501  cd UCE2022/
  502  ls
  503  cd GeneticMap/
  504  ls
  505  ls -l book*
  506  more book.txt
  507  more book2.txt
  508  ls
  509  cd ..
  510  ls
  511  cd cd
  512  cd
  513  ls -l *pl
  514  cd ATLAS2
  515  ls
  516  ls -l *pl
  517  vi vrGVdatabaseATLAS2_A2.pl
  518  cd
  519  cd ../patrick/
  520  ls
  521  cd 2500GENOMES
  522  ls
  523  less -S ALL.chr19.phase3_shapeit2_mvncall_integrated_v5a.20130502.genotypes.vcf
  524  exit
  525  ls
  526  cd ..
  527  ls
  528  cd patrick/
  529  ls
  530  cd 2500GENOMES/
  531  ls
  532  less -S sub average {
  533  my @array = @_; # save the array passed to this function
  534  my $sum; # create a variable to hold the sum of the array's values
  535  foreach (@array) { $sum += $_; } # add each element of the array
  536  # to the sum
  537  return $sum/@array; # divide sum by the number of elements in the
  538  # array to find the mean
  539  }
  540  @dataArray = (1, 2, 3, 4, 5, 6, 7, 8, 9);
  541  less -S ALL.chr19.phase3_shapeit2_mvncall_integrated_v5a.20130502.genotypes.vcf
  542  ls
  543  cd
  544  ls
  545  ls -l boo*
  546  more book.txt
  547  more book2.txt
  548  ls
  549  cd IBC2022
  550  ls
  551  vi RegEx1.pl
  552  perl RegEx1.pl
  553  vi RegEx1.pl
  554  perl RegEx1.pl
  555  vi RegEx1.pl
  556  perl RegEx1.pl
  557  vi RegEx1.pl
  558  perl RegEx1.pl
  559  vi RegEx1.pl
  560  perl RegEx1.pl
  561  vi RegEx1.pl
  562  perl RegEx1.pl
  563  vi RegEx1.pl
  564  perl RegEx1.pl
  565  ls
  566  vi RegEx1.pl
  567  perl RegEx1.pl
  568  ls
  569  vi RegEx1.pl
  570  perl RegEx1.pl
  571  vi RegEx1.pl
  572  perl RegEx1.pl
  573  vi RegEx1.pl
  574  perl RegEx1.pl
  575  vi RegEx1.pl
  576  perl RegEx1.pl
  577  vi RegEx1.pl
  578  perl RegEx1.pl
  579  vi RegEx1.pl
  580  perl RegEx1.pl
  581  vi RegEx1.pl
  582  perl RegEx1.pl
  583  vi RegEx1.p
  584  vi RegEx1.pl
  585  perl RegEx1.pl
  586  vi RegEx1.pl
  587  perl RegEx1.pl
  588  vi RegEx1.pl
  589  perl RegEx1.pl
  590  vi RegEx1.pl
  591  perl RegEx1.pl
  592  vi RegEx1.pl
  593  perl RegEx1.pl
  594  vi RegEx1.pl
  595  perl RegEx1.pl
  596  vi RegEx1.pl
  597  perl RegEx1.pl
  598  vi RegEx1.pl
  599  perl RegEx1.pl
  600  vi RegEx1.pl
  601  perl RegEx1.pl
  602  vi RegEx1.pl
  603  perl RegEx1.pl
  604  vi RegEx1.pl
  605  perl RegEx1.pl
  606  exit
  607  ls
  608  vi 1000GfreqSNPs.pl
  609  cat > 1000GfreqSNPs.pl
  610  vi 1000GfreqSNPs.pl
  611  ls ../patrick/
  612  ls ../patrick/2500GENOMES/
  613  vi 1000GfreqSNPs.pl
  614  less -S ../patrick/2500GENOMES/ALL.chr19.phase3_shapeit2_mvncall_integrated_v5a.20130502.genotypes.vcf
  615  vi 1000GfreqSNPs.pl
  616  perl 1000GfreqSNPs.pl
  617  ls
  618  cat >2504ids
  619  perl 1000GfreqSNPs.pl
  620  vi 1000GfreqSNPs.pl
  621  cat >igsr_samples.tsv
  622  ls
  623  perl 1000GfreqSNPs.pl
  624  perl 1000GfreqSNPs.pl 19
  625  vi 1000GfreqSNPs.pl
  626  less -S ../patrick/2500GENOMES/ALL.chr19.phase3_shapeit2_mvncall_integrated_v5a.20130502.genotypes.vcf
  627  vi 1000GfreqSNPs.pl
  628  perl 1000GfreqSNPs.pl 19
  629  vi 1000GfreqSNPs.pl
  630  perl 1000GfreqSNPs.pl 19
  631  vi 1000GfreqSNPs.pl
  632  perl 1000GfreqSNPs.pl 19
  633  vi 1000GfreqSNPs.pl
  634  perl 1000GfreqSNPs.pl 19
  635  vi 1000GfreqSNPs.pl
  636  perl 1000GfreqSNPs.pl 19
  637  vi 1000GfreqSNPs.pl
  638  perl 1000GfreqSNPs.pl 19
  639  vi 1000GfreqSNPs.pl
  640  perl 1000GfreqSNPs.pl 19
  641  vi 1000GfreqSNPs.pl
  642  perl 1000GfreqSNPs.pl 19
  643  vi 1000GfreqSNPs.pl
  644  perl 1000GfreqSNPs.pl 19
  645  vi 1000GfreqSNPs.pl
  646  perl 1000GfreqSNPs.pl 19
  647  vi 1000GfreqSNPs.pl
  648  perl 1000GfreqSNPs.pl 19
  649  vi 1000GfreqSNPs.pl
  650  perl 1000GfreqSNPs.pl 19
  651  vi 1000GfreqSNPs.pl
  652  perl 1000GfreqSNPs.pl 19
  653  vi 1000GfreqSNPs.pl
  654  perl 1000GfreqSNPs.pl 19
  655  vi 1000GfreqSNPs.pl
  656  ls
  657  rm nohup.out
  658  ls ../patrick/2500GENOMES/
  659  ls -l *pl
  660  vi 1000GfreqSNPs.pl
  661  nohup perl 1000GfreqSNPs.pl &
  662  more nohup.out
  663  vi 1000GfreqSNPs.pl
  664  nohup perl 1000GfreqSNPs.pl &
  665  more nohup.out
  666  vi 1000GfreqSNPs.pl
  667  rm nohup.out
  668  nohup perl 1000GfreqSNPs.pl &
  669  more nohup.out
  670  ls
  671  more nohup.out
  672   ls ../patrick/2500GENOMES/
  673   ls -l ../patrick/2500GENOMES/
  674  ls
  675  vi 1000GfreqSNPs.pl
  676  nohup perl 1000GfreqSNPs.pl &
  677  ls
  678  more nohup.out
  679  ls
  680  ls -l *pl
  681  vi 1000GfreqSNPs.pl
  682  cd UCE2022/
  683  ls
  684  ls -l
  685  more hg19_UCNE_coord2.bed_sorted
  686  ls -l
  687  vi UCEprog1.pl
  688  ls
  689  cd GeneticMap/
  690  ls
  691  vi GeneticMap
  692  vi GeneticMap.pl
  693  pwd
  694  ls /home/patrick/
  695  ls /home/patrick/2500GENOMES/
  696  less -S ALL.chr19.phase3_shapeit2_mvncall_integrated_v5a.20130502.genotypes.vcf
  697  less -S /home/patrick/2500GENOMES/ALL.chr19.phase3_shapeit2_mvncall_integrated_v5a.20130502.genotypes.vcf
  698  pwd
  699  ls
  700  pwd
  701  vi   GeneticMap.pl
  702  cd
  703  ls -l *pl
  704  vi 1000GfreqSNPs.pl
  705  cd UCE2022/GeneticMap/
  706  ls
  707  cp GeneticMap.pl  UCEsnps.pl
  708  vi UCEsnps.pl
  709  more genetic_map_GRCh37_chr15.txt
  710  ls
  711  ls ..
  712  more ../hg19_UCNE_coord2.bed_sorted
  713  vi UCEsnps.pl
  714  perl UCEsnps.pl
  715  ls
  716  history 40
  717  more ../../1000GfreqSNPs.pl
  718  ls
  719  vi UCEsnps.pl
  720  perl UCEsnps.pl
  721  ls
  722  vi UCEsnps.pl
  723  perl UCEsnps.pl
  724  ls
  725  ls -l *pl
  726  vi 1000GfreqSNPs.pl
  727  nohup perl 1000GfreqSNPs.pl &
  728  cd UCE2022/GeneticMap/
  729  ls
  730  vi UCEsnps.pl
  731  perl UCEsnps.pl
  732  ls
  733  cd
  734  more nohup.out
  735  ls
  736  more nohup.out
  737  history
  738  history |grep perl
  739  ls -l *pl
  740  vi  1000GfreqSNPs.pl
  741   nohup perl 1000GfreqSNPs.pl &
  742  ls
  743  cd UCE2022/GeneticMap/
  744  ls
  745  vi UCEsnps.pl
  746  perl UCEsnps.pl
  747  vi UCEsnps.pl
  748  perl UCEsnps.pl
  749  vi UCEsnps.pl
  750  history
  751  less -S /home/patrick/2500GENOMES/ALL.chr19.phase3_shapeit2_mvncall_integrated_v5a.20130502.genotypes.vcf
  752  vi UCEsnps.pl
  753  perl UCEsnps.pl
  754  ls
  755  ls ..
  756  more ../hg19_UCNE_coord2.bed_sorted
  757  vi UCEsnps.pl
  758  perl UCEsnps.pl
  759  tail -30  ../hg19_UCNE_coord2.bed_sorted
  760  vi UCEsnps.pl
  761  perl UCEsnps.pl
  762  vi UCEsnps.pl
  763  perl UCEsnps.pl
  764  tail -30  ../hg19_UCNE_coord2.bed_sorted
  765  1637583516376234NRIP1_Joshua22575
  766  211637773816378047NRIP1_Virginia22577
  767  211642401016424348NRIP1_Zhong22578
  768  211790677817907195chr21_Aaron22607
  769  211795195417952219chr21_Abraham22609
  770  213429113334291434OLIG2_Eduardo22673
  771  213432748734327783OLIG2_Joshua22675
  772  213435050734350710OLIG2_Laura22678
  773  213438078734380996OLIG2_Midori22681
  774  213446803834468399OLIG2_Ryan22682
  775  213819023738190464chr21_Akiko22730
  776  213888558438886338chr21_Eden22741
  777  213888721738887502chr21_Faust22743
  778  214003388140034120chr21_Frodo22751
  779  214452135344521629chr21_Gabriel22783
  780  vi UCEsnps.pl
  781  perl UCEsnps.pl
  782  perl UCEsnps.pl >chr21_SNPs
  783  tail -30  ../hg19_UCNE_coord2.bed_sorted
  784  more chr21_SNPs
  785  tail -30  ../hg19_UCNE_coord2.bed_sorted
  786  vi UCEsnps.pl
  787  perl UCEsnps.pl
  788  history
  789  tail -30  ../hg19_UCNE_coord2.bed_sorted |cat -vent
  790  exit
  791  ls
  792  cd UCE2022/GeneticMap/
  793  ls
  794  vi UCEsnps.pl
  795  cd UCE2022/
  796  ls
  797  cd GeneticMap/
  798  ls
  799  vi UCEsnps.pl
  800  perl UCEsnps.pl
  801  ls
  802  cat -vent new_coordinates
  803  vi UCEsnps.pl
  804  perl UCEsnps.pl
  805  vi UCEsnps.pl
  806  perl UCEsnps.pl
  807  cd
  808  more nohup.out
  809  exit
  810  ls nohup
  811  more nohup.out
  812  ls
  813  ls -l *pl
  814  cd UCE2022/
  815  ls
  816  ls -l *pl
  817  cd GeneticMap/
  818  ls -l *pl
  819  ls
  820  cd UCE2022/
  821  cd GeneticMap/
  822  ls
  823  vi UCEsnps.pl
  824  ls
  825  pwd
  826  ls -l *pl
  827  vi UCEsnps.pl
  828  more ../hg19_UCNE_coord2.bed_sorted
  829  cat -vent  ../hg19_UCNE_coord2.bed_sorted |more
  830  ls
  831  vi UCEsnps.pl
  832  more new_coordinates
  833  ls
  834  cat -vent new_coordinates |more
  835  vi UCEsnps.pl
  836  perl UCEsnps.pl
  837  ls -l
  838  vi UCEsnps.pl
  839  perl UCEsnps.pl
  840  ls -l
  841  less -S SNPsUCEvcf_22
  842  vi UCEsnps.pl
  843  perl UCEsnps.pl
  844  less -S SNPsUCEvcf_22
  845  vi UCEsnps.pl
  846  perl UCEsnps.pl 21
  847  ls
  848  less -S SNPsUCEvcf_21
  849  tail -30 new_coordinates
  850  ls
  851  vi startUCEcoordinates.pl
  852  perl startUCEcoordinates.pl
  853  ls
  854  top
  855  exit
  856  ls
  857  cd UCE2022/
  858  cd GeneticMap/
  859  ls
  860  ls -l
  861  wc SNPsUCEvcf_10
  862  wc SNPsUCEvcf_11
  863  vi UCEsnps.pl
  864  perl UCEsnps.pl 20
  865  ls -l
  866  vi startUCEcoordinates.pl
  867  perl startUCEcoordinates.pl
  868  top
  869  ls -l
  870  top
  871  history
  872  ls
  873  wc SNPsUCEvcf_12
  874  vi UCEsnps.pl
  875  perl UCEsnps.pl 20
  876  ls -l
  877  vi startUCEcoordinates.pl
  878  perl startUCEcoordinates.pl
  879  top
  880  ls -l
  881  top
  882  ls
  883  cd ..
  884  ls -l
  885  cd
  886  ls
  887  ls -l *pl
  888  vi 1000GfreqSNPs.pl
  889  cp 1000GfreqSNPs.pl UCE2022/GeneticMap/
  890  cd UCE2022/GeneticMap/
  891  ls
  892  mv 1000GfreqSNPs.pl 1000GfreqSNPsUCE.pl
  893  vi 1000GfreqSNPsUCE.pl
  894  top
  895  ls -l
  896  top
  897  ls -l
  898  top
  899  ls -l
  900  top
  901  ls -l
  902  top
  903  ls
  904  ls -l *pl
  905  vi  1000GfreqSNPsUCE.pl
  906  perl  1000GfreqSNPsUCE.pl
  907  pwd
  908  ls
  909  ls -l
  910  vi UCEsnps.pl
  911  less -S SNPsUCEvcf_1
  912  ls
  913  vi 1000GfreqSNPsUCE.pl
  914  perl 1000GfreqSNPsUCE.pl
  915  ls
  916  vi 1000GfreqSNPsUCE.pl
  917  less -S SNPsUCEvcf_1
  918  vi 1000GfreqSNPsUCE.pl
  919  less -S SNPsUCEvcf_1
  920  vi 1000GfreqSNPsUCE.pl
  921  less -S SNPsUCEvcf_1
  922  vi 1000GfreqSNPsUCE.pl
  923  less -S SNPsUCEvcf_1
  924  vi 1000GfreqSNPsUCE.pl
  925  exit
  926  ls
  927  cd UCE2022/
  928  cd GeneticMap/
  929  ls
  930  ls -l *pl
  931  perl 1000GfreqSNPsUCE.pl
  932  ls
  933  cd 1000GENOMES/
  934  ls
  935  ls -l
  936  more chr3_4columns
  937  ls -l
  938  ls
  939  history
  940  cd UCE2022/GeneticMap/
  941  vi 1000GfreqSNPsUCE.pl
  942  pwd
  943  ls
  944  ls -l *pl
  945   vi 1000GfreqSNPsUCE.pl
  946   perl 1000GfreqSNPsUCE.pl
  947   vi 1000GfreqSNPsUCE.pl
  948  more ../../2504ids
  949  less -S ../../2504ids
  950  cat -vent ../../2504ids
  951   vi 1000GfreqSNPsUCE.pl
  952  more ../../igsr_samples.tsv
  953   vi 1000GfreqSNPsUCE.pl
  954  ls
  955  cat -vent  SNPsUCEvcf_18  |more
  956  ls
  957  head -253  SNPsUCEvcf_18 |tail -1
  958  head -253  SNPsUCEvcf_18 |tail -1 >2504indsApr18
  959  ls
  960  less -S 2504indsApr18
  961   cp  1000GfreqSNPsUCE.pl UCEsnpINDapr18.pl
  962  vi UCEsnpINDapr18.pl
  963  perl UCEsnpINDapr18.pl
  964  less -S SNPsUCEvcf_19
  965  vi UCEsnpINDapr18.pl
  966  perl UCEsnpINDapr18.pl
  967  less -S SNPsUCEvcf_19
  968  vi UCEsnpINDapr18.pl
  969  perl UCEsnpINDapr18.pl
  970  vi UCEsnpINDapr18.pl
  971  perl UCEsnpINDapr18.pl
  972  vi UCEsnpINDapr18.pl
  973  perl UCEsnpINDapr18.pl
  974  exit
  975  ls
  976  pwd
  977  cd UCE2022/GeneticMap/
  978  ls
  979  ls -l *pl
  980  vi  UCEsnpINDapr18.pl
  981  perl  UCEsnpINDapr18.pl
  982  ls
  983  less -S SNPsUCEvcf_11
  984  vi  UCEsnpINDapr18.pl
  985  less -S SNPsUCEvcf_11
  986  vi  UCEsnpINDapr18.pl
  987  perl  UCEsnpINDapr18.pl
  988  vi  UCEsnpINDapr18.pl
  989  perl  UCEsnpINDapr18.pl
  990  vi  UCEsnpINDapr18.pl
  991  perl  UCEsnpINDapr18.pl
  992  eixt
  993  exit
  994  ls
  995  ls -l *pl
  996  vi 1000GfreqSNPs.pl
  997  cd UCE2022/GeneticMap/
  998  ls
  999  ls -l *pl
 1000  vi UCEsnpINDapr18.pl
 1001  cd UCE2022/GeneticMap/
 1002  ls -l *pl
 1003  vi  UCEsnpINDapr18.pl
 1004  perl  UCEsnpINDapr18.pl
 1005  vi  UCEsnpINDapr18.pl
 1006  perl  UCEsnpINDapr18.pl
 1007  vi  UCEsnpINDapr18.pl
 1008  perl  UCEsnpINDapr18.pl
 1009  vi  UCEsnpINDapr18.pl
 1010  perl  UCEsnpINDapr18.pl
 1011  vi  UCEsnpINDapr18.pl
 1012  perl  UCEsnpINDapr18.pl
 1013  vi  UCEsnpINDapr18.pl
 1014  perl  UCEsnpINDapr18.pl >test
 1015  more test
 1016  sort -k5 -n test
 1017  history
[afedorov@intron2 GeneticMap]$
```

Conclusions:

I calculated numbers of alternative SNP alleles with frequencies <2% inside all individuals from 2504 1000G project. The least number was 22 and the highest was 157:

```
[afedorov@intron2 GeneticMap]$ sort -k5 -n test |head -20
2382	NA20796	TSI	EUR	22
1795	NA18539	CHB	EAS	23
2224	NA19749	MXL	AMR	24
1459	HG03785	ITU	SAS	25
1820	NA18570	CHB	EAS	25
2026	NA19081	JPT	EAS	25
2036	NA19091	JPT	EAS	25
2217	NA19732	MXL	AMR	25
244	HG00566	CHS	EAS	25
520	HG01515	IBS	EUR	25
704	HG01927	PEL	AMR	25
726	HG01968	PEL	AMR	25
800	HG02105	PEL	AMR	25
911	HG02360	CDX	EAS	25
1943	NA18969	JPT	EAS	26
194	HG00403	CHS	EAS	26
2326	NA20527	TSI	EUR	26
139	HG00318	FIN	EUR	27
141	HG00320	FIN	EUR	27
1850	NA18619	CHB	EAS	27


[afedorov@intron2 GeneticMap]$ sort -k5 -n test |tail -20
2060	NA19144	YRI	AFR	146
2158	NA19446	LWK	AFR	146
1097	HG02804	GWD	AFR	147
1118	HG02861	GWD	AFR	147
1182	HG03066	MSL	AFR	147
1199	HG03097	MSL	AFR	147
2058	NA19141	YRI	AFR	147
2152	NA19437	LWK	AFR	147
771	HG02052	ACB	AFR	147
1003	HG02585	GWD	AFR	148
1181	HG03064	MSL	AFR	149
1186	HG03074	MSL	AFR	150
1988	NA19023	LWK	AFR	150
1077	HG02760	GWD	AFR	151
1354	HG03583	MSL	AFR	151
1911	NA18923	YRI	AFR	151
2140	NA19397	LWK	AFR	151
995	HG02570	GWD	AFR	152
2123	NA19360	LWK	AFR	156
1184	HG03072	MSL	AFR	157
```

The highest numbers all from Africa. The lowest from EAS (51%), EUR (22%), AMR (13%), SAS (13%), and AFR (1% from ASW).

Inside the file 2504indsSNPs_insideUCE_MAF2percent

The cutoff was MAF alternative alleles <2%. If we considered frequent SNPs alleles which frequency is >50%, then somebody may criticize us that the frequent allele is not a mutation inside UCE, but its complementary allele. This should force the overestimation of mutations inside UCEs.

I made archive:

tar -cvf UCE2022april21.tar UCE2022

gzip UCE2022april21.tar

sftp 79.120.70.98

The file UCE2022april21.tar.gz is also on CRI MAC:

Alexeis-iMac:UCEs2022Ali afedorov$ pwd

/Users/afedorov/Documents/UCEs2022Ali

**May 31, 2022**

Analysis of presence of UCE sequences in non-coding RNA databases.

1) Downloaded the latest version of NONCODE v6 human RNAs from the web. Secondly, used UCSC noncoding RNA database of human from Rajib’s old folders in BPGN. Transferred these two databases in Moscow BIO. /home/afedorov/LincRNA folder as files: NONECODElincRNAnew and lncRNA.fa (which was ‘>’ info line truncated version of UCSC_lncRNAs_SEQ) (/home/rajib/LINC_RNA/UCSC_lnc_RNA_files)
2) In Moscow BIO installed BLAST by a command line:

sudo apt install ncbi-blast+

after this run blast by the following commands:

```
makeblastdb -in   lncRNA.fa  -dbtype nucl
blastn -query UCEfasta_sorted.txt -db lncRNA.fa  -evalue 0.0001 -num_alignments 1 -out blast_UCSC
blastn -query UCEfasta_sorted.txt -db NONECODElincRNAnew  -evalue 0.0001 -num_alignments 1 -out blast_nonecode
```

the output files contained NON-matched UCE entries:

afedorov@bio:~/LincRNA$ grep 'No hits' blast_nonecode |wc

**3730**	18650	96980

afedorov@bio:~/LincRNA$. grep 'No hits' blast_UCSC |wc

**3557**	17785	92482

the input query contained 4273 UCE sequences:

afedorov@bio:~/LincRNA$ grep '>' UCEfasta_sorted.txt |wc

**4273**	12819 216858

Therefore, 87.3% of UCEs do not match NONECODE and 83.5% UCEs do not match UCSC lncRNAs. NONCODE database represents about 11% of the human genome, while UCSC represents about 19% of the human genome (see below the number of characters in the databases, while human genome of sequenced portion is about 2,7 GB. Therefore, the match of small portion of UCE sequences with ncRNA DB are random!

```
wc NONECODElincRNAnew
  5947903   5947903 293,191,046 NONECODElincRNAnew
afedorov@bio:~/LincRNA$ wc lncRNA.fa
 10142516  10142516 516,497,407 lncRNA.fa

afedorov@bio:~/LincRNA$ grep -v '>' lncRNA.fa  |wc
10127460 10127460 516,136,063
afedorov@bio:~/LincRNA$ grep -v '>'  NONECODElincRNAnew |wc
5774791 5774791 290,248,142
```

**June 22, 2022**

**Paper Results Figures/Tables. IN: UCEs2022Ali/UCEmaterialsPAPER**

1) Supplementary Figure S1: BLAST alignment FTO human-chicken a) mRNA, b) UCE intronic

Files: AnalysisUCE1vicinities.docx and FTOmRNAhumanChicken.docx.

2) Table 1 distribution SNPs by MAF frequency in whole Genome and UCE. File: Table1_UCE_SNPfreq2022.xlsx
3) Figure 1 distribution SNPs by MAF frequency in whole Genome and UCE. File: Table1_UCE_SNPfreq2022.xlsx
4) Figure 2 distribution of number of UCE mutations inside human individuals from 1000G. File: UCEmutationsPOPULATIONSxlsx.
5) Table 2. Statistics of UCE mutations inside human individuals from 1000G. File: PPT slides. The table is in file: UCEmutationsPOPULATIONSxlsx.
6) Table 3. Number of UCE inside non-coding RNAs (See Protocols Page 20-21 May 31.)
7) Table 4. Meiotic Recombination rates inside UCEs
8) Supplementary Table S2: Oligonucleotide distributions inside UCEs

File: COMP_UCEdbNAR (supporting files/programs are in UCEs2022Ali/UCEdatabase). See also protocols Page 1: February 26, 2022.

Genomic Signature inside UCEs vs Whole Human Genome (For whole-genome NT composition see Protocols on Page 3 chr16. )

**August 25, 2022**

HISTORY:

```
cd UCE2022
  848  ls
  849  more  hg19_UCNE_coord2.bed_sorted
  850  head  hg19_UCNE_coord2.bed_sorted
  851  wc  hg19_UCNE_coord2.bed_sorted
  852  ls
  853  more  UCEfasta_sorted.txt
  854  ls
  855  grep '>'  UCEfasta_sorted.txt
  856  grep '>'  UCEfasta_sorted.txt |wc
  857  wc  hg19_UCNE_coord2.bed_sorted
  858  head -30  hg19_UCNE_coord2.bed_sorted
  859  grep '>'  UCEfasta_sorted.txt |head -30
  860  tail -30  hg19_UCNE_coord2.bed_sorted
  861  grep '>'  UCEfasta_sorted.txt |tail -30
  862  grep '>'  UCEfasta_sorted.txt |wc
  863  wc  hg19_UCNE_coord2.bed_sorted
  864  ls
  865  more UCEfasta_sorted.txt
  866  ls
  867  more hg19_UCNE_coord2.bed_sorted
  868      ls
  869  more README.txt
  870  vi  UCEprog1.pl
  871  perl  UCEprog1.pl
  872  vi  UCEprog1.pl
  873  perl  UCEprog1.pl
  874  ls
  875  grep '>' hg19_UCNEs.fasta.txt |wc
  876  wc hg19_UCNE_coord.bed
  877  perl  UCEprog1.pl >missingUCEfasta78
  878  clear
  879  ls
  880  ls -l
  881  head hg19_UCNE_coord.bed
  882  head hg19_UCNE_coord2.bed
  883  tail hg19_UCNE_coord.bed
  884  tail hg19_UCNE_coord2.bed
  885  ls -l
  886  cat -vent hg19_UCNE_coord.bed |head
  887  cat -vent hg19_UCNE_coord2.bed |head
  888  tail hg19_UCNE_coord.bed
  889  ls -l
  890  grep '>'  hg19_UCNEs.fasta.txt |wc
  891  wc  hg19_UCNE_coord.bed
  892  vi  coordinates.pl
  893  more missingUCEfasta78
  894  ls
  895  cd GeneticMap/
  896  ls
  897  ls -l *pl
  898  vi UCEsnpINDapr18.pl
  899  ls
  900  cd UCE2022
  901  ls
  902  cd GeneticMap/
  903  ls
  904  ls -l *pl
  905  vi UCEsnpINDapr18.pl
  906  ls -l
  907  more SNPsUCEvcf_11
  908  less -S  SNPsUCEvcf_11
  909  ls -l *pl
  910  vi UCEsnps.pl
  911  ls -l
  912  vi UCEsnps.pl
  913  vi  1000GfreqSNPsUCE.pl
  914  cd ..
  915  ls
  916  vi UCEprog1.pl
  917  ls -l
  918  wc  hg19_UCNE_coord2.bed_sorted
  919  grep '>' UCEfasta_sorted.txt |wc
  920  vi UCEprog1.pl
  921  perl UCEprog1.pl
  922  ls
  923  wc  hg19_UCNE_coord2.bed_sorted4273
  924  more   hg19_UCNE_coord2.bed_sorted4273
  925  ls
  926  grep '>' UCEfasta_sorted.txt
  927  grep '>' UCEfasta_sorted.txt |wc
  928  ls
  929  cp hg19_UCNE_coord2.bed_sorted4273 GeneticMap/
  930  cd GeneticMap/
  931  ls
  932  ls -l *pl
  933  vi UCEsnps.pl
  934  perl UCEsnps.pl 22
  935  ls -l
  936  vi startUCEcoordinates.pl
  937  perl startUCEcoordinates.pl
  938  ls
  939  cd UCE2022
  940  ls
  941  cd GeneticMap/
  942  ls -l
  943  top
  944  ls -l
  945  ls
  946  history -50
  947  history 50
  948   vi UCEsnps.pl
  949   perl UCEsnps.pl 22
  950   vi UCEsnps.pl
  951   perl startUCEcoordinates.pl
  952  ls -l
  953  top
  954  ls -l
  955  top
  956  ls -l
  957  ls
  958  ls -l *X*
  959  ls -l
  960  ls
  961  history 50
  962   perl UCEsnps.pl X
  963  ls  /home/patrick/2500GENOMES
  964  vi  UCEsnps.pl
  965   perl UCEsnps.pl X
  966  top
  967  ls -l
  968  vi  UCEsnps.pl
  969  ls -l *pl
  970  vi 1000GfreqSNPsUCE.pl
  971  perl 1000GfreqSNPsUCE.pl
  972  vi 1000GfreqSNPsUCE.pl
  973  perl 1000GfreqSNPsUCE.pl
  974  ls
  975  ls -l *pl
  976  vi GeneticMap_AF.pl
  977  perl GeneticMap_AF.pl
  978  vi GeneticMap_AF.pl
  979  perl GeneticMap_AF.pl
  980  vi GeneticMap_AF.pl
  981  perl GeneticMap_AF.pl
  982  ls -l *pl
  983  vi  UCEsnpINDapr18.pl
  984  perl  UCEsnpINDapr18.pl
  985  vi  UCEsnpINDapr18.pl
  986  perl  UCEsnpINDapr18.pl >resultsAug24_UCEsnpINDs
  987  cd UCE2022
  988  cd GeneticMap/
  989  ls
  990  ls -l *pl
  991  vi  UCEsnpINDapr18.pl
  992  perl  UCEsnpINDapr18.pl  resultsAug24_UCEsnpINDs50percent
  993  ls
  994  perl  UCEsnpINDapr18.pl > resultsAug24_UCEsnpINDs50percent
  995  vi  UCEsnpINDapr18.pl
  996  ls
  997  more resultsAug24_UCEsnpINDs50percent
  998  perl  UCEsnpINDapr18.pl > resultsAug24_UCEsnpINDs50percent
  999  more resultsAug24_UCEsnpINDs50percent
 1000  vi  UCEsnpINDapr18.pl
```

**August 28, 2022**

Genomic signature for Chr 1 calculated on BPGN:

```
T	0.292	67244164
C	0.208	48055043
G	0.209	48111528
[N]	0.926	230481012
[!N]	0.074	18475410


AA	0.0950	21901540
AT	0.0743	17121783
AC	0.0503	11598278
AG	0.0714	16448644
TA	0.0631	14554789
TT	0.0957	22048241
TC	0.0601	13844699
TG	0.0729	16796378
CA	0.0728	16768284
CT	0.0713	16444797
CC	0.0541	12466763
CG	0.0103	2375159
GA	0.0601	13845615
GT	0.0505	11629291
GC	0.0440	10145272
GG	0.0542	12491312
```

**September 11, 2022**

```
Counting Genomic signatures in BPGN

-bash-4.1$ history 50
  985  ls /home/shuhao/HumanGenome_Jan2015
  986  perl NTcomposition.pl /home/shuhao/HumanGenome_Jan2015/chr1 NTcompositionCHR1 8
  987  ls
  988  history 20
  989  nohup  perl NTcomposition.pl /home/shuhao/HumanGenome_Jan2015/chr1 NTcompositionCHR1 8 &
  990  ls
  991  top
  992  ls
  993  top
  994  q
  995  ls
  996  top
  997  ls
  998  ls -l
  999  more  NTcompositionCHR1
 1000  ls
 1001  history
 1002  ls
 1003  ls -l NT*
 1004  more NTcompositionCHR1
 1005  ls -l NT*
 1006  vi NTcomposition.pl
 1007  ls
 1008  history
 1009  ls -l NT*
 1010  nohup  perl NTcomposition.pl /home/shuhao/HumanGenome_Jan2015/chr2 NTcompositionCHR2 8 &
 1011  ls -l *pl
 1012  ls  get_UCEseq.pl
 1013  vi  get_UCEseq.pl
 1014  vi GenomicSignature.pl
 1015  perl  GenomicSignature.pl
 1016  vi GenomicSignature.pl
 1017  perl  GenomicSignature.pl
 1018  vi GenomicSignature.pl
 1019  perl  GenomicSignature.pl
 1020  vi GenomicSignature.pl
 1021  perl  GenomicSignature.pl
 1022  vi GenomicSignature.pl
 1023  perl  GenomicSignature.pl
 1024  vi GenomicSignature.pl
 1025  perl  GenomicSignature.pl
 1026  ls -l *pl
 1027  vi  NTcomposition.pl
 1028  vi GenomicSignature.pl
 1029  perl  GenomicSignature.pl
 1030  vi GenomicSignature.pl
 1031  perl  GenomicSignature.pl
1080  vi startGenSignature.pl
 1081  perl startGenSignature.pl
1082  perl startGenSignature.pl >GenomicSignaturesAllChromosomes
```

**September 12, 2022**

```
879  ls  get_UCEseq.pl
  880  vi  get_UCEseq.pl
  881  vi GenomicSignature.pl
  882  perl  GenomicSignature.pl
  883  vi GenomicSignature.pl
  884  perl  GenomicSignature.pl
  885  vi GenomicSignature.pl
  886  perl  GenomicSignature.pl
  887  vi GenomicSignature.pl
  888  perl  GenomicSignature.pl
  889  vi GenomicSignature.pl
  890  perl  GenomicSignature.pl
  891  vi GenomicSignature.pl
  892  perl  GenomicSignature.pl
  893  ls -l *pl
  894  vi  NTcomposition.pl
  895  vi GenomicSignature.pl
  896  perl  GenomicSignature.pl
  897  vi GenomicSignature.pl
  898  perl  GenomicSignature.pl
  899  top
  900  ls
  901  history 50
  902  nohup  perl NTcomposition.pl /home/shuhao/HumanGenome_Jan2015/chr3 NTcompositionCHR3 8 &
  903  nohup  perl NTcomposition.pl /home/shuhao/HumanGenome_Jan2015/chr4 NTcompositionCHR4 8 &
  904  nohup  perl NTcomposition.pl /home/shuhao/HumanGenome_Jan2015/chr5 NTcompositionCHR5 8 &
  905  nohup  perl NTcomposition.pl /home/shuhao/HumanGenome_Jan2015/chr6 NTcompositionCHR6 8 &
  906  nohup  perl NTcomposition.pl /home/shuhao/HumanGenome_Jan2015/chr7 NTcompositionCHR7 8 &
  907  nohup  perl NTcomposition.pl /home/shuhao/HumanGenome_Jan2015/chr8 NTcompositionCHR8 8 &
  908  nohup  perl NTcomposition.pl /home/shuhao/HumanGenome_Jan2015/chr9 NTcompositionCHR9 8 &
  909  nohup  perl NTcomposition.pl /home/shuhao/HumanGenome_Jan2015/chr10 NTcompositionCHR10 8 &
  910  nohup  perl NTcomposition.pl /home/shuhao/HumanGenome_Jan2015/chr11 NTcompositionCHR11 8 &
  911  nohup  perl NTcomposition.pl /home/shuhao/HumanGenome_Jan2015/chr12 NTcompositionCHR12 8 &
  912  top
  913  nohup  perl NTcomposition.pl /home/shuhao/HumanGenome_Jan2015/chr13 NTcompositionCHR13 8 &
  914  nohup  perl NTcomposition.pl /home/shuhao/HumanGenome_Jan2015/chr14 NTcompositionCHR14 8 &
  915  nohup  perl NTcomposition.pl /home/shuhao/HumanGenome_Jan2015/chr15 NTcompositionCHR15 8 &
  916  nohup  perl NTcomposition.pl /home/shuhao/HumanGenome_Jan2015/chr17 NTcompositionCHR17 8 &
  917  nohup  perl NTcomposition.pl /home/shuhao/HumanGenome_Jan2015/chr18 NTcompositionCHR18 8 &
  918  nohup  perl NTcomposition.pl /home/shuhao/HumanGenome_Jan2015/chr19 NTcompositionCHR19 8 &
  919  nohup  perl NTcomposition.pl /home/shuhao/HumanGenome_Jan2015/chr20 NTcompositionCHR20 8 &
  920  nohup  perl NTcomposition.pl /home/shuhao/HumanGenome_Jan2015/chr21 NTcompositionCHR21 8 &
  921  nohup  perl NTcomposition.pl /home/shuhao/HumanGenome_Jan2015/chr22 NTcompositionCHR22 8 &
  922  ls /home/shuhao/HumanGenome_Jan2015/
  923  nohup  perl NTcomposition.pl /home/shuhao/HumanGenome_Jan2015/chrX NTcompositionCHRX 8 &
  924  nohup  perl NTcomposition.pl /home/shuhao/HumanGenome_Jan2015/chrY NTcompositionCHRY 8 &
  925  ls -l NT*
  926  ls
  927  history 50
  928  vi GenomicSignature.pl
  929  perl GenomicSignature.pl 1
  930  vi GenomicSignature.pl
  931  perl GenomicSignature.pl 1
  932  perl GenomicSignature.pl 2
  933  perl GenomicSignature.pl 3
  934  top
  935  ls -l NT*
  936  vi GenomicSignature.pl
  937  perl GenomicSignature.pl 2
  938  perl GenomicSignature.pl X
  939  perl GenomicSignature.pl Y
  940  vi startGenSignature.pl
  941  perl startGenSignature.pl
  942  vi startGenSignature.pl
  943  perl startGenSignature.pl
  944  ls -l *pl
  945  grep system  MAGE_Jan2017_expD.pl
  946  grep system
  947  vi startGenSignature.pl
  948  perl startGenSignature.pl
  949  perl startGenSignature.pl >GenomicSignaturesAllChromosomes
  950  ls
  951  history
  952  ls
  953  more GenomicSignaturesAllChromosomes
  954  grep CG GenomicSignaturesAllChromosomes
  955  ls
  956  ls -l *pl
  957  more GenomicSignature.pl
  958  vi GenomicSignature.pl
  959  cp GenomicSignature.pl GenomicSignatureALL.pl
  960  vi  GenomicSignatureALL.pl
  961  perl  GenomicSignatureALL.pl 1
  962  vi  GenomicSignatureALL.pl
  963  vi startGenSignature.pl
  964  vi  GenomicSignatureALL.pl
  965  perl  GenomicSignatureALL.pl
  966  vi  GenomicSignatureALL.pl
  967  perl  GenomicSignatureALL.pl
  968  vi  GenomicSignatureALL.pl
  969  perl  GenomicSignatureALL.pl
  970  vi  GenomicSignatureALL.pl
  971  perl  GenomicSignatureALL.pl
  972  vi  GenomicSignatureALL.pl
  973  perl  GenomicSignatureALL.pl
  974  vi  GenomicSignatureALL.pl
  975  perl  GenomicSignatureALL.pl
  976  vi  GenomicSignatureALL.pl
  977  perl  GenomicSignatureALL.pl
  978  vi  GenomicSignatureALL.pl
  979  perl  GenomicSignatureALL.pl
  980  vi  GenomicSignatureALL.pl
  981  perl  GenomicSignatureALL.pl
  982  vi  GenomicSignatureALL.pl
  983  perl  GenomicSignatureALL.pl
  984  vi  GenomicSignatureALL.pl
  985  perl  GenomicSignatureALL.pl
  986  vi  GenomicSignatureALL.pl
  987  perl  GenomicSignatureALL.pl
  988  vi  GenomicSignatureALL.pl
  989  perl  GenomicSignatureALL.pl
  990  ls
  991  perl  GenomicSignatureALL.pl > NTcompositionCHRall
  992  ls -l *pl
  993  vi GenomicSignature.pl
  994  perl GenomicSignature.pl all
  995  vi  GenomicSignatureALL.pl
  996  vi startGenSignature.pl
  997  perl startGenSignature.pl
  998  ls
  999  perl startGenSignature.pl >GenomicSignaturesAllChromosomes
 1000  more GenomicSignaturesAllChromosomes
 1001  ls
 1002  ls *ll
 1003  more NTcompositionCHRall
 1004  more  NTcompositionCHR1
 1005  ls
 1006  ls -l NT*
 1007  more  NTcompositionCHR10.tbl
 1008  history
```
